# Ketone Oximes as NO_2_ Radical Precursors for Regioselective Iodo–Nitrosylative Cyclization of Unactivated 1,6-Enynes

**DOI:** 10.1021/acs.joc.5c02201

**Published:** 2026-01-02

**Authors:** Mohana Reddy Mutra, T. L. Chandana, Yu-Syuan Chu, Chien-Hung Li, Jeh-Jeng Wang

**Affiliations:** † Department of Medicinal and Applied Chemistry, 38023 Kaohsiung Medical University, No. 100, Shiquan first Rd, San-min District, Kaohsiung City 807, Taiwan; ‡ Department of Medical Research, Kaohsiung Medical University Hospital, No. 100, Tzyou first Rd, Sanmin District, Kaohsiung City 807, Taiwan

## Abstract

A metal-free three-component radical cascade cyclization of unactivated 1,6-enynes has been developed using 1-phenylethan-1-one oxime as a novel in situ nitrogen dioxide (NO_2_) radical source under I_2_/aqueous TBHP conditions. This transformation proceeds through a chemo- and regioselective sequence involving NO_2_· addition to the terminal alkene, 5-exo-dig cyclization onto the alkyne, and iodine trapping of the resulting carbon-centered radical, yielding 4-(iodo­(phenyl)­methylene)-3-(nitromethyl)-1-tosylpyrrolidines. This strategy avoids transition metals, photocatalysts, or prefunctionalized nitrogen reagents and highlights the previously unexplored potential of oximes as bench-stable NO_2_-radical donors for cascade transformations.

## Graphical abstract

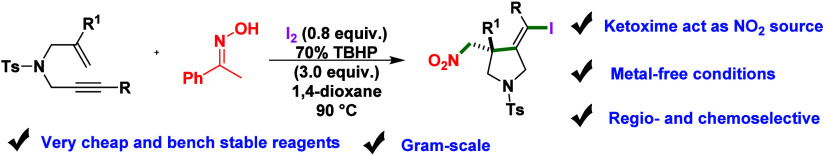

## Introduction

Nitrogen dioxide (NO_2_) radicals are highly reactive electrophilic species that play important roles in synthetic radical chemistry.^1^ Their use has enabled diverse transformations such as direct C–H nitration, alkene difunctionalization, and heterocycle construction.^1,2^ Despite their synthetic potential, controlled and safe generation of NO_2_· radicals under practical conditions remains a persistent challenge due to the hazardous and oxidative nature of gaseous NO_2_ and nitric oxide (NO).^1c^ To circumvent these issues, a variety of in situ NO_2_· sources have been developed (Scheme 1, eq A). These include inorganic nitrites such as NaNO_2_,^1b^ KNO_2_,^3a^ AgNO_2_,^3b^ and LiNO_2_ ^3c^ in combination with oxidants like K_2_S_2_O_8_, PhI­(OAc)_2_, TBHP, and oxidants.^1–3^ Organic nitrites including *tert*-butyl nitrite (tBuONO)^1c,1d^ and isoamyl nitrite (iAmONO) have also been employed under oxidative conditions to liberate NO_2_·.^1c,1d,4^ In addition, N-nitrosamines,^5a^ N-nitrosoureas,^5b^ and aryldiazonium salts^5c^ with nitrite additives have been utilized to generate nitrogen-centered radicals, though often with limited generality or stability.^5^ In addition to the nitrating reagents discussed above, numerous other methods have been developed in organic synthesis. These include classical mineral acid–based systems,^6a^ metal nitrate/acid combinations,^6b^ and various organic nitrites,^1g,2b,6c,6d^ which, despite their widespread use, often suffer from harsh reaction conditions, limited substrate scope, or insufficient selectivity. Moreover, many of these reagents are expensive, require storage under specific low- temperature conditions, and involve multiple steps to prepare the desired nitrating agents for use.^1–6^ Despite this diversity, a general, safe, and bench-stable NO_2_ radical precursor that functions under mild, additive-free conditions remains elusive. Even enzymatic systems, such as cytochrome P450-mediated oxidation of oximes, have been shown to produce NO_2_ radicals; however, such biological strategies are difficult to translate into general synthetic protocols.^7^ Importantly, to the best of our knowledge, no chemical methods have yet demonstrated the direct use of oximes—simple and readily available compounds derived from ketones—as NO_2_· radical precursors for synthetic radical functionalization.^8^ In particular, their application in radical cascade cyclizations of 1,6-enynes has remained unexplored, despite the fact that such transformations provide a powerful platform for the rapid construction of densely functionalized heterocycles with high regio- and stereoselectivity.

**Scheme 1:**
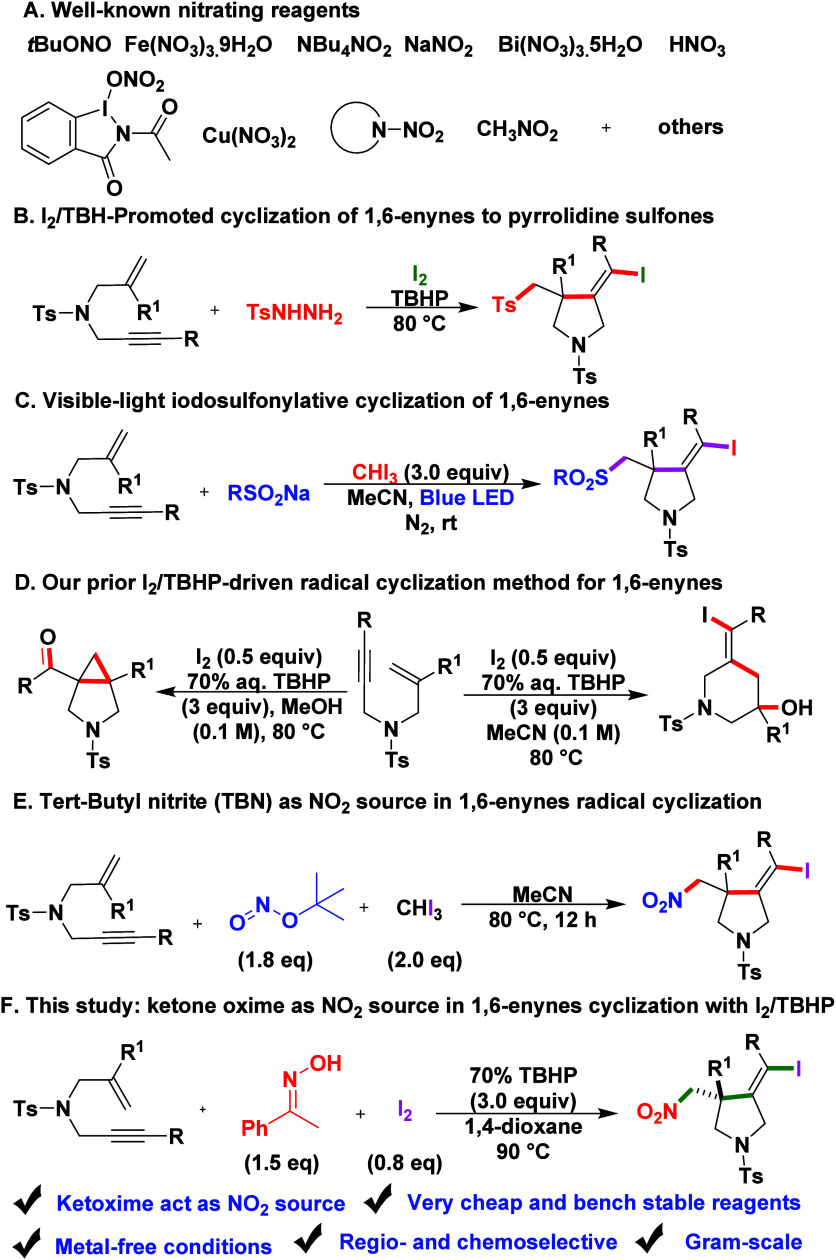
(A) Well-Known Nitrating Reagents Organic Synthesis^a^

a (A) Well-known nitrating reagents. (B) I_2_/TBHP-promoted cyclization of 1,6-enynes to pyrrolidine sulfones. (C) Visible-Light iodosulfonylative cyclization of 1,6-enynes. (D) Our prior I_2_/TBHP-driven radical cyclization method for 1,6-enynes. (E) Tert-butyl nitrite (TBN) as NO_2_ source in 1,6-enynes radical cyclization. (F) This study: ketone oxime as NO_2_ Source in unactivated 1,6-enynes cyclization with I_2_/TBHP.

Nitrogen-tethered 1,6-enynes combine an alkene and alkyne linked through a nitrogen-containing chain, making them ideal for radical cascade cyclizations.^9^ This structure directs selective radical addition and intramolecular cyclization steps, enabling efficient, regio- and chemoselective formation of nitrogenous heterocycles—such as pyrrolidines—under mild, metal and metal-free conditions. Using I_2_/TBHP, these enynes undergo one-pot radical cyclization with sulfonyl hydrazides to give five-membered sulfonylated pyrrolidines with C–S and C–I bonds in a single step, providing access to functional pyrrolidine sulfones (Scheme 1, eq B).^10^ Separately, visible-light-driven iodosulfonylative cyclization of enynes with iodoform and sulfinates enables sulfonyl radical generation under catalyst- and oxidant-free conditions, giving vinyl iodide containing sulfones (Scheme 1, eq C).^11^ In our previous studies, we extensively explored the reactivity of 1,6-enynes^12^ /1,6-diyne^13^ or as versatile substrates for the construction of densely substituted nitrogen-containing heterocycles under metal-free conditions. We showed that under I_2_/TBHP-mediated radical cascade cyclization, solvent choice critically influences the reaction pathway and product outcome, enabling selective access to diverse nitrogenous heterocycles without metal catalysts (Scheme 1, eq D).^12a^ Zhu and co-workers reported a three-component, metal-free iodonitrosylative cyclization of 1,6-enynes, in which *tert*-butyl nitrite (TBN) generates nitrogen-centered radicals that either undergo a radical cascade as ·NO followed by final oxidation to NO_2_, or are first oxidized to NO_2_ radical before initiating the cascade, leading to alkene addition, 5-*exo*-dig cyclization, and iodine trapping (Scheme 1, eq E).^14^ Herein, we report a three-component, metal- and photocatalyst-free radical cascade cyclization of nitrogen-tethered unactivated 1,6-enynes, utilizing ketoximes as in situ sources of nitrogen dioxide (NO_2_) radicals in the presence of I_2_ and TBHP (Scheme 1, eq F). This strategy enables efficient construction of iodo–nitromethylated pyrrolidines through a sequential NO_2_-radical addition, 5-*exo*-dig cyclization, iodination, and oxidation sequence. The ketoxime-mediated pathway provides a distinct and practical approach for nitrogen-centered radical generation, allowing selective access to highly functionalized nitrogenous heterocycles under mild, metal-free conditions.

## Results and Discussion

The initial investigation of this methodology was conducted using 4-methyl-N-(2-phenylallyl)-N-(3-phenylprop-2-yn-1-yl)­benzenesulfonamide (**1a**) and 1-phenylethan-1-one oxime (**1b**) as the model substrates for synthesizing (*Z*)-4-(iodo­(phenyl)­methylene)-3-(nitromethyl)-3-phenyl-1-tosylpyrrolidine (**3a**), as summarized in Table 1. Under optimized conditions employing molecular iodine (I_2_, 0.8 equiv) and aqueous *tert*-butyl hydroperoxide (aq TBHP, 3.0 equiv) in 1,4-dioxane at 90 °C for 6 h, the desired product **3a** was obtained in 79% isolated yield (Table 1, entry 1). Alternative iodine sources, including N-iodosuccinimide (NIS), sodium iodide (NaI), potassium iodide (KI), tetrabutylammonium iodide (TBAI), and phenyliodine­(III) diacetate (PIDA), were evaluated but resulted in significantly diminished or negligible product formation (Table 1, entries 2–6). Subsequently, a range of oxidants such as *tert*-butyl hydroperoxide in decane (TBHP, 5.5 M), di*tert*-butyl peroxide (DTBP), cumene hydroperoxide (CHP), hydrogen peroxide (H_2_O_2_), and potassium persulfate (K_2_S_2_O_8_) were screened; however, none of these improved the yield of product **3a** compared to the optimized condition (Table 1, entries 7–11). Further solvent screening with various media failed to enhance the reaction efficiency (Table 1, entries 12–16). Modifying the stoichiometry of iodine, aqueous TBHP, and oxime also led to yields comparable to the initial optimized conditions, ranging between 75 and 78% (Table 1, entries 17–19). Notably, conducting the reaction at room temperature was ineffective, and no desired product was observed (Table 1, entry 20). Control experiments demonstrated that in the absence of either iodine or aqueous TBHP, the reaction did not proceed, confirming the essential role of both reagents in the transformation (Table 1, entries 21 and 22). Based on these results, the conditions outlined in entry 1 of Table 1 were selected as the standard protocol for further substrate scope exploration and product derivatization.

**Table 1:**
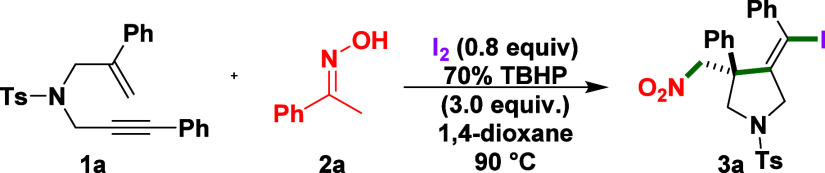
Screening of the Reaction Conditions^a^,^b^

entry	I source (*y* equiv)	oxidant (*x* equiv)	solvent	yield^c^
1	I_2_	aq TBHP	dioxane	79
2^d^	NaI	aq TBHP	dioxane	48
3^d^	KI	aq TBHP	dioxane	40
4^d^	TBAI	aq TBHP	dioxane	28
5^d^	PIDA	aq TBHP	dioxane	NR
6^d^	NIS	aq TBHP	dioxane	72
7	I_2_	TBHP^e^	dioxane	73
8	I_2_	DTBP	dioxane	15
9	I_2_	CHP	dioxane	20
10	I_2_	H_2_O_2_ ^f^	dioxane	trace
11	I_2_	K_2_S_2_O_8_	dioxane	NR
12	I_2_	aq TBHP	THF	60
13	I_2_	aq TBHP	EtOH	10
14	I_2_	aq TBHP	toluene	50
15	I_2_	aq TBHP	DMSO	NR
16	I_2_	aq TBHP	DMF	NR
17^g^	I_2_	aq TBHP	dioxane	75
18^h^	I_2_	aq TBHP	dioxane	73
19^i^	I_2_	aq TBHP	dioxane	78
20^j^	I_2_	aq TBHP	dioxane	NR
21	I_2_		dioxane	0
22	I_2_	aq TBHP	dioxane	0

a Reaction conditions: **1a** (0.1 mmol, 1.0 equiv), **2a** (1.5 equiv), iodine source (0.8 equiv), aq TBHP (70% in H_2_O; 3.0 equiv) in 1,4-dioxane (0.1 M) at 90 °C (oil bath) for 6 h unless otherwise noted.

b Note: TBHP; tertiary butyl hydroperoxide. DTBP; ditertbutyl hydroperoxide. CHP; cumene hydroperoxide. H_2_O_2_; dihydrogen peroxide. K_2_S_2_O_8_; potassium persulfate.

c Isolated yields.

d 1.6 equiv of iodine source was used.

e 5.5 M TBHP in decane was used.

f H_2_O_2_; 30 wt % in H_2_O.

g 1.5 equiv of iodine source was used.

h 5.0 equiv of aq TBHP was used.

i 2.0 equiv of compound was used.

j The reaction performed at RT.

With the optimized reaction conditions established, we next explored the substrate scope to evaluate the generality and functional group tolerance of the radical iodo–nitrosylative cyclization (Table 2). A variety of unactivated 1,6-enynes bearing different substituents on both the alkyne and alkene termini were subjected to the standard conditions. The substituent at the alkyne terminus (denoted as R) could be varied widely. Aryl groups bearing electron-donating substituents such as phenyl (**1a**), *ortho*-methylphenyl (**1b**), *ortho*-methoxyphenyl (**1c**), *meta*-methylphenyl (**1d**), *para*-methylphenyl (**1e**), *para*-methoxyphenyl (**1f**), were well tolerated, affording the corresponding pyrrolidine products (**3a**–**3f**) in good to excellent yields (59–79%). Notably, the ortho-substituted substrates **1b** (o-Me) and **1c** (o-OMe) afforded nearly racemic diastereomeric mixtures (**3b**: dr = 50:50; **3c**: dr = 45:50), likely due to steric congestion and, in the case of o-OMe, additional electronic effects, which perturb the radical addition and 5-*exo*-trig cyclization steps.^14^ The structure of compound **3a** (CCDC; 2475514) was unambiguously confirmed by single-crystal X-ray diffraction. Electron-withdrawing groups, such as m-Br–Ph (**1g**), m-Cl–Ph (**1h**), m-F–Ph (**1i**), and m-CF_3_–Ph (**1j**) were also compatible, affording the corresponding halogen-substituted products **3g**–**3j** in good yields (64–73%). Furthermore, other strong electron-withdrawing groups, including *m*-NO_2_–Ph (**1k**), *p*-COMe–Ph (**1l**), *p*-CO_2_Me–Ph (**1m**), and *p*-CN–Ph (**1n**), were well tolerated, delivering the corresponding products **3k**–**3n** in good yields (50–62%). Notably, a heteroaromatic substituent, such as thiophene (**1o**), was also compatible under the reaction conditions, affording the single *Z*-isomer product **3o** in excellent yield (81%). Replacement of the R^1^ phenyl group with a methyl group (**1p**) afforded product **3p** in 45% yield with a 10:90 *E/Z* ratio; similarly, when R^1^ was hydrogen (**1q**), the reaction gave the corresponding product **3q** in 41% yield with a 5:95 *E/Z* ratio. A terminal alkyne substrate (R = H, **1r**) afforded product **3r** in 55% yield, but with lower stereoselectivity (*E*/*Z* = 29:71), likely due to reduced steric hindrance and increased rotational freedom around the C–C triple bond. Similarly, an aliphatic-substituted alkyne (R = Me, **1s**) delivered the corresponding pyrrolidine **3s** in 50% yield with moderate stereoselectivity (*E*/*Z* = 43:57). Overall, the method exhibits excellent functional group tolerance, scalability, and stereoselectivity with control over *E*/*Z* isomer formation, making it a broadly applicable strategy for the construction of highly functionalized nitrogen heterocycles.

**Table 2:**
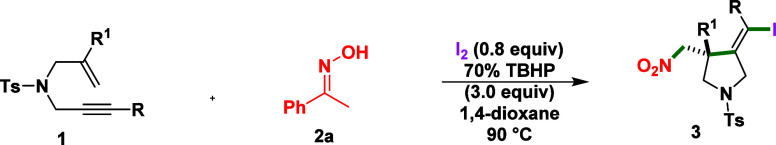

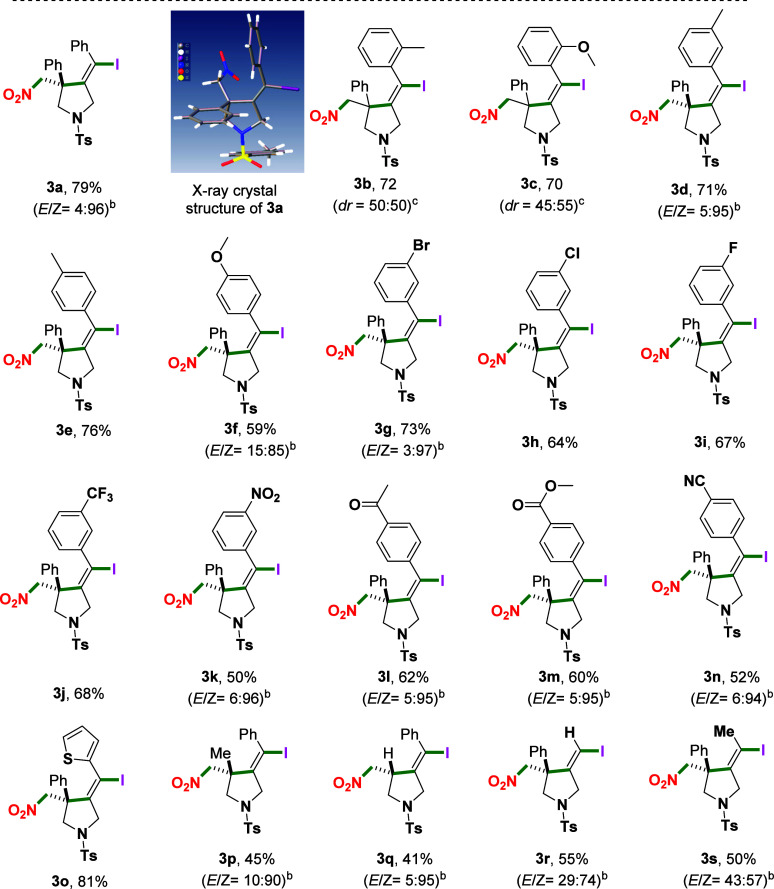
Scope of 1,6-Enynes^a^

a Reaction conditions: **1** (0.1 mmol), 1-phenylethan-1-one oxime (**2a**; 0.15 mmol), I_2_ (0.08 mmol), aq TBHP (70% in H_2_O; 0.3 mmol), 1,4-Dioxane (0.1 M) at 90 °C (oil bath); *Z* isomer is formed; isolated yields.

b Mixtures of *E*/*Z* isomers were formed (The *E*/*Z* ratios were determined based on ^1^H NMR).

c Mixtures of diastereomers were formed (The dr ratios were determined based on ^1^H NMR).

As illustrated in Scheme 2A, the scalability of the radical iodo–nitrosylative cyclization was demonstrated by performing the reaction on a 2.5 mmol scale using substrate **1a** under the optimized conditions. The transformation proceeded efficiently, affording product **3a** in 70% isolated yield with an excellent *E*/*Z* ratio of 5:95, without any alteration to the standard procedure. This gram-scale reaction underscores the robustness and practicality of the method for the synthesis of functionalized pyrrolidines.

**Scheme 2:**
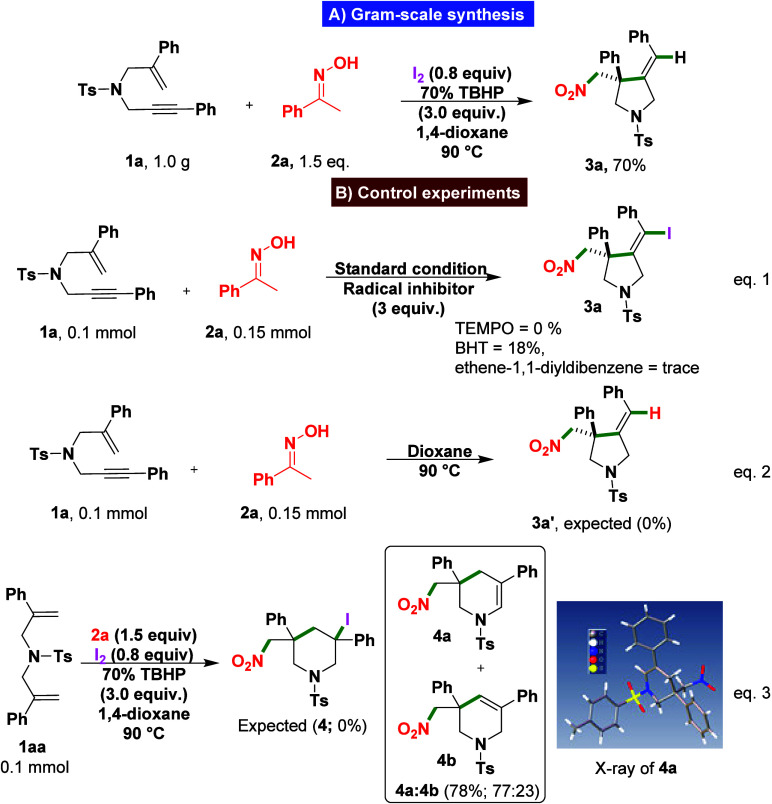
(A) Gram-Scale Synthesis. (B) Control experiments to investigate the reaction mechanism

To elucidate the reaction mechanism, a series of control experiments were performed, as illustrated in Scheme 2B. The addition of the radical scavenger 2,2,6,6-tetramethylpiperidine-1-oxyl (TEMPO) completely suppressed the formation of product **3a** (Scheme 2B, eq 1). Similarly, the presence of butylated hydroxytoluene (BHT) significantly inhibited the reaction, affording only 18% yield of product **3a** (Scheme 2B, eq 1). When ethene-1,1-diyldibenzene was employed as a radical trapping agent, merely trace amounts of product **3a** were detected (Scheme 2B, eq 1). These results provide strong evidence for the involvement of radical intermediates in the catalytic cycle. The reaction between 1,6-enyne and the ketone oxime did not yield the expected product under thermal conditions in the absence of iodine and oxidant, demonstrating that both are essential for the transformation (Scheme 2B, eq 2). Furthermore, when 4-methyl-*N*,*N*-bis­(2-phenylallyl)­benzenesulfonamide (**1aa**) was subjected to the standard reaction conditions, the expected radical cyclization product 3-iodo-5-(nitromethyl)-3,5-diphenyl-1-tosylpiperidine (**4**) was not observed. Instead, stabilization of the intermediate tertiary carbon-centered radical promoted β-elimination over iodine trapping, affording a mixture of 3-(nitromethyl)-3,5-diphenyl-1-tosyl-1,2,3,4-tetrahydropyridine (**4a**) and 3-(nitromethyl)-3,5-diphenyl-1-tosyl-1,2,3,6-tetrahydropyridine (**4b**) in 78% combined yield and a 77:23 ratio (**4a**/**4b**) (Scheme 2B, eq 3). The structure of major isomer **4a** (CCDC; 2504228) was unambiguously confirmed by single-crystal X-ray diffraction.

Based on literature precedent and control experiments^1–12,14–16^ a mechanistic scenario for the radical iodo–nitrosylative cyclization leading to product **3a** is proposed (Scheme 3). In Path A, the ketone oxime undergoes oxidation under I_2_/TBHP conditions to generate a nitric oxide (NO·) radical,^15,16a^ which is subsequently oxidized in situ by TBHP to the highly electrophilic nitrogen dioxide (NO_2_·) radical,^14,15^ initiating the cascade. The NO_2_· radical undergoes regioselective addition to the terminal alkene of the 1,6-enyne, forming a β-nitroalkyl carbon-centered radical (**Int-I**).^14^ This intermediate undergoes a 5-*exo*-dig cyclization onto the adjacent alkyne, generating a vinyl radical (**Int-II**), which is trapped by molecular iodine to form the C­(sp^2^)–I bond, delivering the (*Z*)-configured iodo­(nitromethyl)­methylene pyrrolidine, **3a**. In Path B, the NO· radical may directly add to the terminal alkene of the 1,6-enyne, forming a β-nitrosoalkyl radical intermediate (**Int-III**). Cyclization via a 5-*exo*-dig mode generates a vinyl radical (**Int-IV**), which is subsequently trapped by iodine to afford a vinyl iodide bearing a nitroso substituent (**Int-V**). In situ oxidation of the nitroso group by TBHP then furnishes the corresponding nitro group, converging on product **3a**.^14^ While Path A emphasizes early NO_2_· radical involvement, Path B highlights sequential NO· addition followed by oxidation, with both pathways leading to the observed product selectivity.^14,15^

**Scheme 3:**
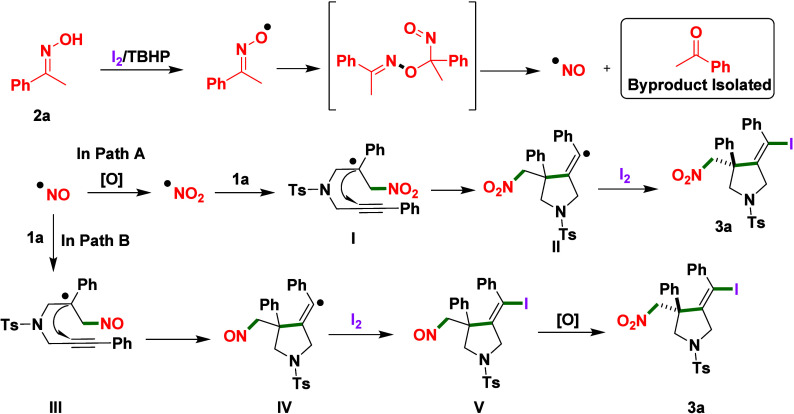
Proposed Reaction Mechanism Involving a Radical Cascade Pathway

## Conclusions

In summary, we report a metal-free, three-component radical iodo–nitrosylative cyclization of unactivated 1,6-enynes using readily available ketone oximes as nitrogen-centered radical precursors. Under I_2_/TBHP oxidative conditions, the reaction proceeds via a highly regio- and chemoselective radical cascade, delivering structurally complex pyrrolidines bearing both nitro and vinyl iodide functionalities in good to excellent yields. Mechanistically, the transformation may occur through either direct initiation by nitrogen dioxide (NO_2_·) radicals generated from the oxime, or via initial addition of nitric oxide (NO·) radicals followed by in situ oxidation of the intermediate nitroso species to the nitro derivative. The operational simplicity, broad substrate scope, and scalability underscore the synthetic utility of this approach and establish ketone oximes as versatile, bench-stable nitrogen-centered radical precursors. This strategy offers a sustainable platform for the rapid construction of densely functionalized nitrogen heterocycles.

## Supplementary Material



## Data Availability

The data underlying this study are available in the published article and its Supporting Information.

## Notes

Experimental procedures; spectral characterizations; copies of ^1^H, ^13^C {^1^H} NMR spectra for all products (PDF)
