# Supplementary material for: Ketone Oximes as NO2 Radical Precursors for Regioselective Iodo–Nitrosylative Cyclization of Unactivated 1,6-Enynes
Source: J Org Chem. 2026 Jan 2;91(2):966–72. doi: 10.1021/acs.joc.5c02201 (PMC12814559; doi:10.1021/acs.joc.5c02201)
Supplement: Supplementary file 1 [file jo5c02201_si_001.pdf]

Supporting Information

For

Ketone Oximes as NO_2 Radical Precursors for Regioselective Iodo–Nitrosylative Cyclization of Unactivated 1,6-Enynes

Mohana Reddy Mutra,[†] T. L. Chandana,[†] Yu-Syuan Chu,[†] Chien-Hung Li,[†] Jeh-Jeng Wang*[,†,‡]

[†]Department of Medicinal and Applied Chemistry, Kaohsiung Medical University, No. 100, Shih-Chuan 1st Rd, Sanmin District, Kaohsiung City, 807 (Taiwan).

[‡]Department of Medical Research, Kaohsiung Medical University Hospital, No. 100, Tzyou 1st Rd, Sanmin District, Kaohsiung City, 807 (Taiwan).

E-mail: jjwang@kmu.edu.tw

Table of Contents

1. General information…………………………………………………………………………S3

2. Preparation of starting materials……………………………………………………………….S4

2.1. General procedure for the synthesis of 4-methyl-*N*-(2-phenylallyl)-*N*-(3-phenylprop-2-yn-1-yl)benzenesulfonamidederivatives (**1a**) ………………………………………………………………S4-S5

2.2. Alternative route for the synthesis of 1,6-enyne derivatives (**1a**) ………………………………..S4-S5

2.3. Synthesis of 1-phenylethan-1-one oxime (**2a**)……………………………………………………S6

3. Experimental procedures……………………………………………………………………………S7

3.1. General procedure for the synthesis of pyrrolidine derivatives **3a**–**3s** ……………………………S7

3.2. Gram-scale synthesis of **3a**…………………………………………………………………..S7

3.3. Synthesis of mixture compounds **4a** and **4b**…..…………………………………………………..S8

4. Characterization data…………………………………………………………………..............S8-S14

5. References……………………………………………………………………………………..S15

6. Solvent system and crystallization method for compounds **3a** and **4a**..S15-S36

6.1. X-ray crystallographic analysis data for compound **3a**……………….…………………………...S16-S20

F**igure S1**. ORTEP view of X-crystal structure of **3a** (the ellipsoid contour 50% probability levels)..S20

6.2. X-ray crystallographic analysis data for compound **4a**……………………………………………S21-S36

F**igure S2**. ORTEP view of X-crystal structure of **4a** (the ellipsoid contour 50% probability levels)..S21

7. Copies of ^{1}H and ^{13}C spectra of the compounds……………………………………………………S37-S76

1. General Information

^{1}H and ^{13}C NMR spectra were recorded on a 400 MHz Varian Unity Plus or Varian Mercury plus spectrometer. The chemical shift (δ) values are reported in parts per million (ppm), and the coupling constants (J) are given in Hz. The spectra were recorded using $CDCl_3$ as a solvent. ^{1}H NMR chemical shifts are referenced to tetramethylsilane (TMS) (0 ppm). ^{13}CNMR was referenced to $CDCl_3$ (77.0 ppm). The abbreviations used are as follows: s, singlet; d, doublet; t, triplet; q, quartet; dd, doublet of doublet; ddd, doublet of doublet of doublet; dt, doublet of triplets; td, triplet of doublet; m, multiplet. Mass spectra and High-Resolution Mass spectral (HRMS) data was carried out using an Agilent 6890N GC (JEOL JMS-700) TOF instrument, and the ion source is electrospray ionization (ESI), ESI-TOF (FT-MS solariX) at National Sun Yat-Sen University, Kaohsiung, Taiwan. The crystallographic measurements were performed on a Rigaku Oxford XtaLab Pro II single-crystal X-ray diffractometer equipped with a microfocus sealed X-ray source and a HyPix detector. Melting points were determined on an EZ-Melt (Automated melting point apparatus). All products reported showed 1H NMR spectra in agreement with the assigned structures. Reaction progress and product mixtures were routinely monitored by TLC using Merck TLC aluminum sheets (silica gel 60 F254). Column chromatography was carried out with 230–400 mesh silica gel 60 (Merck)/neutral alumina and a mixture of hexane/ethyl acetate or hexane as an eluent.

2. Preparation of Starting Materials

2.1. General procedure for the synthesis of 4-methyl-*N*-(2-phenylallyl)-*N*-(3-phenylprop-2-yn-1-yl)benzenesulfonamidederivatives (1a)[1a-b]

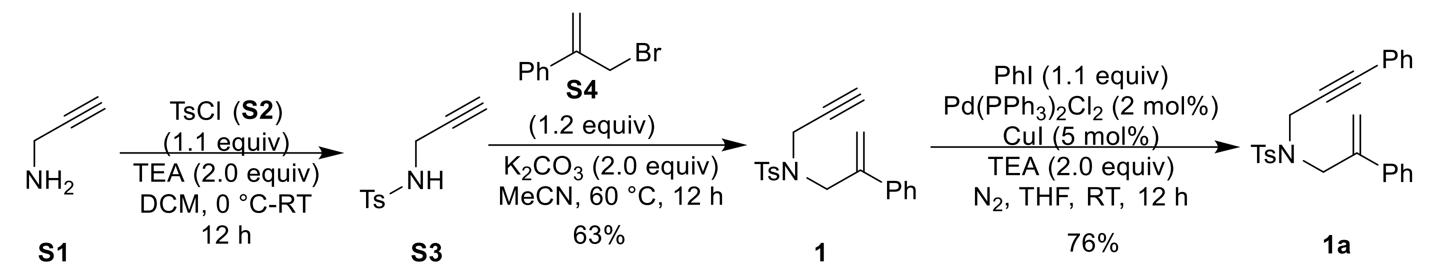

2.1.1. Procedure for synthesis of 4-methyl-*N*-(prop-2-yn-1-yl)benzenesulfonamide (S3)

To a solution of prop-2-yn-1-amine (500 mg, 9.09 mmol, 1.0 equiv) in dichloromethane (DCM) at 0 °C, triethylamine (TEA) (1.83 g, 18.80 mmol, 2.0 equiv) and 4-toluenesulfonyl chloride (1.90 g, 10 mmol, 1.1 equiv) were added. The resulting mixture was allowed to warm to room temperature and stirred for 12 hours. After the reaction was deemed complete, as monitored by thin-layer chromatography (TLC), the reaction mixture was diluted with water and extracted with DCM. The combined organic layers were washed with saturated sodium chloride solution and dried over anhydrous sodium sulfate (Na_2SO_4). The mixture was filtered, and the solvent was removed under reduced pressure to give the crude material. The crude product was washed with pentane and was used in the next step without further purification. The crude material weighed 1.9 g.

2.1.2. Procedure for synthesis of 4-methyl-*N*-(2-phenylallyl)-*N*-(prop-2-yn-1-yl) benzenesulfonamide (1)

To a solution of 4-methyl-N-(prop-2-yn-1-yl)benzenesulfonamide (1.9 g, 8.61 mmol, 1.0 equiv) in acetonitrile (MeCN) at 0 °C, potassium carbonate (K_2CO_3) (2.37 g, 17.22 mmol, 2.0 equiv) and (3-bromoprop-1-en-2-yl)benzene (2.03 g, 10.33 mmol, 1.2 equiv) were added. The resulting mixture was heated to 60 °C (oil bath) and stirred for 12 hours. After the reaction was complete, as monitored by thin-layer chromatography (TLC), the mixture was cooled to room temperature, and the solvent was removed under reduced pressure. The resulting solid was dissolved in ethyl acetate, washed with water and brine, and dried over anhydrous sodium sulfate (Na_2SO_4). The crude product was purified by column chromatography using a hexane/ethyl acetate (80:20) mixture as the eluent. The desired product, 4-methyl-N-(2-phenylallyl)-N-(prop-2-yn-1-yl)benzenesulfonamide, was obtained as a white solid (1.75 g, 63% yield).

2.1.3. Procedure for synthesis of 4-methyl-*N*-(2-phenylallyl)-*N*-(3-phenylprop-2-yn-1-yl)benzenesulfonamide (1a)

To a dried Schlenk flask was added 4-methyl-N-(2-phenylallyl)-N-(prop-2-yn-1-yl)benzenesulfonamide (1.75 g, 5.38 mmol, 1.0 equiv) and iodobenzene (1.20 g, 5.92 mmol, 1.1 equiv) in dry tetrahydrofuran (THF). The reaction mixture was then charged with $Pd(PPh_3)_2Cl_2$ (76 mg, 0.10 mmol, 2 mol%), CuI (51 mg, 0.26 mmol, 5 mol%), and triethylamine (Et_3N) (1.08 g, 10.76 mmol, 2.0 equiv) under a nitrogen atmosphere. The resulting mixture was stirred at room temperature (RT) for 12 hours under nitrogen. After the reaction was complete, as determined by thin-layer chromatography (TLC), the mixture was cooled to RT, diluted with water, and extracted with ethyl acetate. The combined organic layers were dried over anhydrous sodium sulfate (Na_2SO_4), filtered, and concentrated under reduced pressure to afford the crude product. The crude material was purified by column chromatography using a hexane/ethyl acetate (90:10) mixture as the eluent. The desired product, 4-methyl-N-(2-phenylallyl)-N-(3-phenylprop-2-yn-1-yl)benzenesulfonamide, was obtained as a white solid (1.6 g, 76% yield).

2.2. Alternative route for the synthesis of 1,6-enyne derivatives (1a)[1a-b]

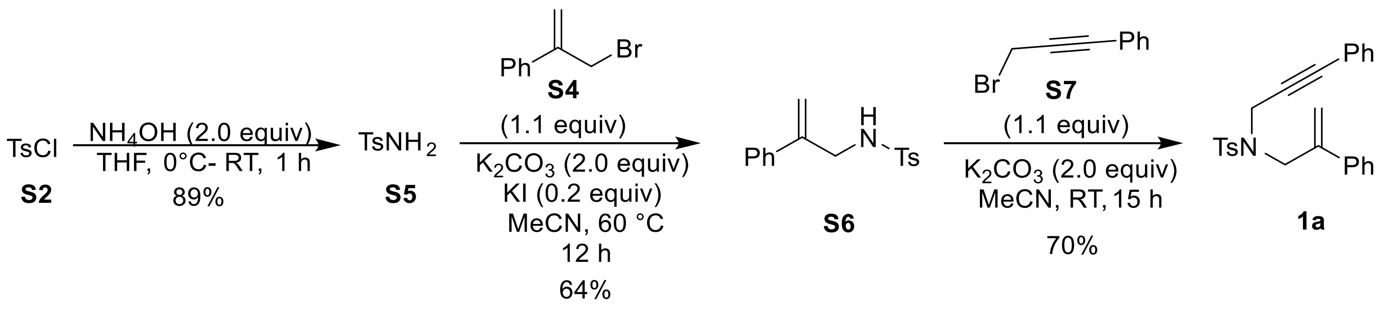

2.2.1. Procedure for synthesis of 4-methylbenzenesulfonamide (S5)

To a solution of 4-toluenesulfonyl chloride (1.5 g, 7.89 mmol, 1.0 equiv) in tetrahydrofuran (THF) at 0 °C, ammonium hydroxide (NH_4OH) (553 mg, 15.78 mmol, 2.0 equiv) was added. The resulting mixture was stirred at room temperature for 1 hour. After the reaction was complete, as monitored by thin-layer chromatography (TLC), the reaction mixture was cooled to 0 °C, then cold water was added. The mixture was quenched with hydrochloric acid (HCl) to adjust the pH to 7 and then extracted with dichloromethane (DCM). The combined organic layers were dried over anhydrous sodium sulfate (Na_2SO_4), filtered, and concentrated under reduced pressure to yield the crude product. The crude material was washed with pentane and carried forward to the next step without further purification, yielding 1.2 g (89%) of the product.

2.2.2. Procedure for synthesis of 4-methyl-*N*-(2-phenylallyl)benzenesulfonamide (S6)

To a solution of 4-methylbenzenesulfonamide (1.2 g, 7.01 mmol, 1.0 equiv) in acetonitrile (MeCN) at 0 °C, potassium carbonate (K_2CO_3) (1.93 g, 14.03 mmol, 2.0 equiv), potassium iodide (KI) (232 mg, 1.40 mmol, 0.2 equiv), and (3-bromoprop-1-en-2-yl)benzene (1.52 g, 7.71 mmol, 1.1 equiv) were added. The resulting mixture was heated at 60 °C (oil bath) for 12 hours. After the reaction was complete, as monitored by thin-layer

chromatography (TLC), the mixture was cooled to room temperature (RT), and the solvent was removed under reduced pressure. The resulting solid was dissolved in ethyl acetate, washed with water and brine, and dried over magnesium sulfate ($MgSO_4$). The solvent was evaporated under reduced pressure, and the crude product was purified by column chromatography to yield 4-methyl-N-(2-phenylallyl)benzenesulfonamide as a white solid (1.3 g, 64% yield). Note: Minor di-alkylation product was also observed.

2.2.3. Procedure for synthesis of 4-methyl-*N*-(2-phenylallyl)-*N*-(3-phenylprop-2-yn-1-yl)benzenesulfonamide (1a)

To a solution of 4-methyl-N-(prop-2-yn-1-yl)benzenesulfonamide (1.3 g, 4.52 mmol, 1.0 equiv) in acetonitrile (MeCN) at room temperature, potassium carbonate (K_2CO_3) (1.25 g, 9.05 mmol, 2.0 equiv) and (3-bromoprop-1-yn-1-yl)benzene (0.966 g, 4.98 mmol, 1.1 equiv) were added. The resulting mixture was stirred at room temperature for 15 hours. After the reaction was complete, as monitored by thin-layer chromatography (TLC), the mixture was diluted with water and extracted with ethyl acetate. The combined organic layers were dried over anhydrous sodium sulfate (Na_2SO_4), filtered, and concentrated under reduced pressure to yield the crude product. The crude material was purified by column chromatography using a hexane/ethyl acetate (90:10) mixture as the eluent to afford 4-methyl-N-(2-phenylallyl)-N-(3-phenylprop-2-yn-1-yl)benzenesulfonamide as a white solid (1.25 g, 70% yield).

Note: Compounds **1b-1s** were synthesized following the above procedure. These compounds are known, and their analytical data match the reported values.[1]

2.3. Synthesis of 1-phenylethan-1-one oxime (2a)[2]

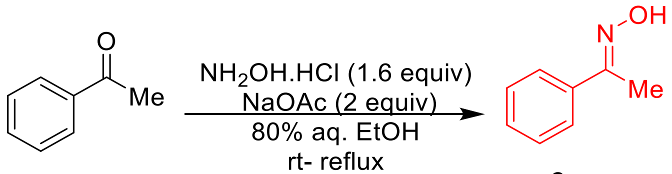

1 **2a**

A suspension of hydroxylamine hydrochloride (1.6 equiv, 1.38 g, 20 mmol) and sodium acetate (2.0 equiv, 1.64 g, 40 mmol) in 80% aqueous ethanol (20 mL) was stirred at room temperature for 30 min. To this mixture, acetophenone (1.0 equiv, 1.20 g, 10 mmol) was added, and the reaction mixture was heated gently to reflux for 3 h (oil bath). After completion of the reaction (monitored by TLC), the reaction mixture was cooled to room temperature, and excess ethanol was removed under reduced pressure. The resulting residue was purified by column chromatography on silica gel using hexane–ethyl acetate (9:1) as eluent to afford 1-phenylethan-1-one oxime as white crystalline solid (2.0 g, quantitative yield).

3. Experimental procedures

3.1. General Procedure for the Synthesis of Pyrrolidine Derivatives 3a–3s

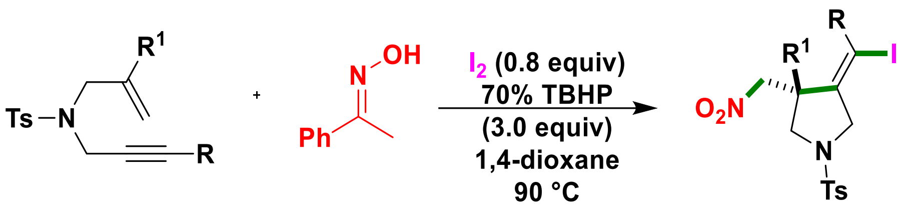

In a dry, sealed reaction tube equipped with a magnetic stir bar, 1,6-enyne (0.10 mmol) was dissolved in 1,4-dioxane (0.1 M). 1-Phenylethan-1-one oxime (0.15 mmol) and iodine (I_2, 0.08 mmol) were added, followed by dropwise addition of tert-butyl hydroperoxide (TBHP, 70% in water, 0.30 mmol). The reaction tube was sealed and heated at 90 °C (oil bath) for 6–15 h, with the reaction progress monitored by thin-layer chromatography (TLC). After cooling to room temperature, the mixture was diluted with ethyl ether (3 × 10 mL). The combined organic layers were washed with saturated brine (10 mL), dried over anhydrous Na_2SO_4, and concentrated under reduced pressure. The crude residue was purified by silica gel column chromatography (hexane/ethyl acetate) to afford the desired product **3** as a solid.

3.2. Gram-Scale Synthesis of (*Z*)-4-(Iodo(phenyl)methylene)-3-(Nitromethyl)-3-phenyl-1-tosylpyrrolidine

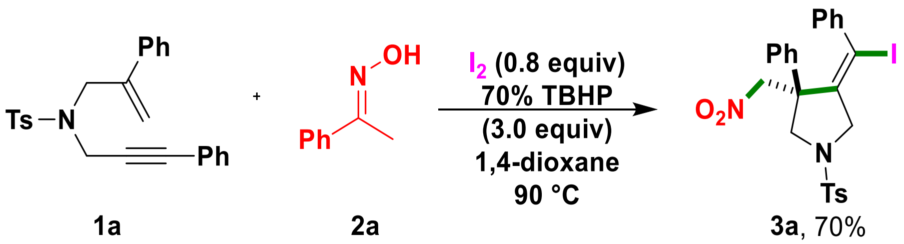

In a dry, sealed reaction tube, 4-methyl-N-(2-phenylallyl)-N-(3-phenylprop-2-yn-1-yl)benzenesulfonamide (1.0 g, 2.5 mmol) was added, followed by the addition of 1,4-dioxane (1.0 mL). The tube was then charged with 1-phenylethan-1-one oxime (3.73 mmol) and I_2 (2.0 mmol). After thoroughly mixing the reagents, tert-butyl hydroperoxide (TBHP, 70% in water, 7.5 mmol) was added dropwise to the reaction mixture. The resulting solution was stirred at 90 °C (oil bath) for 24 hours. The reaction progress was monitored by thin-layer chromatography (TLC). Upon completion, the reaction mixture was cooled to room temperature and extracted with ethyl ether (3 × 50 mL). The combined organic phases were washed with saturated brine (50 mL), dried over anhydrous sodium sulfate (Na_2SO_4), and concentrated under reduced pressure. The crude product was purified by silica gel column chromatography using an 84:16 mixture of hexane/ethyl acetate, as the eluent to afford the desired product **3a** as a solid (1.0 g) in 70% yield.

3.3. Synthesis of mixture of 3-(nitromethyl)-3,5-diphenyl-1-tosyl-1,2,3,4-tetrahydropyridine1-phenylethan-1-one oxime (4a) and 3-(nitromethyl)-3,5-diphenyl-1-tosyl-1,2,3,6-tetrahydropyridine (4b)

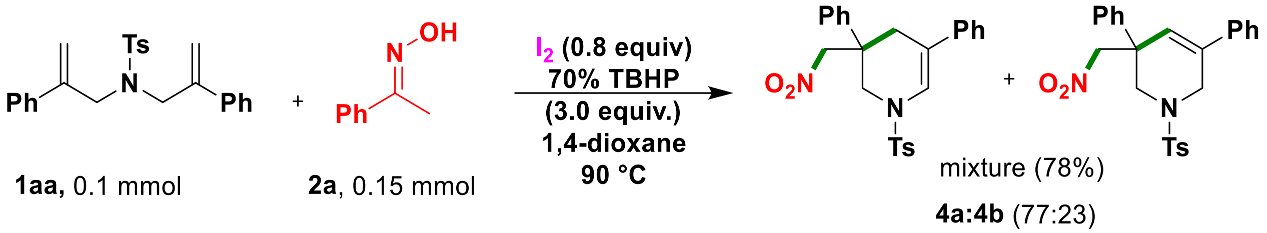

In a dry, sealed reaction tube equipped with a magnetic stir bar, 4-methyl-N,N-bis(2-phenylallyl)benzenesulfonamide (**1aa**) (41 mg, 0.1 mmol) was dissolved in 1,4-dioxane (0.1 M). 1-Phenylethan-1-one oxime (21 mg, 0.15 mmol) and iodine (I_2) (21 mg, 0.08 mmol) were added, followed by dropwise addition of tert-butyl hydroperoxide (TBHP, 70% in water, 0.30 mmol). The reaction tube was sealed and heated at 90 °C (oil bath) for 6 h, with the reaction progress monitored by thin-layer chromatography (TLC). After cooling to room temperature, the mixture was diluted with ethyl ether (3 × 10 mL). The combined organic layers were washed with saturated brine (10 mL), dried over anhydrous Na_2SO_4, and concentrated under reduced pressure. The crude residue was purified by silica gel column chromatography (hexane/ethyl acetate) to afford the mixture of products **4a:4b** (77:23) in 78% (36 mg) as a solid.

4. Characterization data

4-(iodo(phenyl)methylene)-3-(nitromethyl)-3-phenyl-1-tosylpyrrolidine (3a):[3] The title compound was prepared according to the general procedure and purified by silica gel column chromatography (eluent: hexane/ethyl acetate, 85:15) to afford a mixture of E/Z isomers (4:96) as a white solid (47 mg, 79% yield); Mp. 179-181 °C; ^{1}**H NMR** (400 MHz, $CDCl_3$) δ 7.75 (d, *J* = 8.2 Hz, 2H), 7.40 (d, *J* = 8.1 Hz, 2H), 7.26 – 7.18 (m, 3H), 7.12 (t, *J* = 7.4 Hz, 1H), 7.03 (t, *J* = 7.3 Hz, 2H), 6.95 (d, *J* = 6.7 Hz, 2H), 6.63 (brs, 2H), 4.65 (d, *J* = 14.5 Hz, 1H), 4.40 – 4.30 (m, 2H), 4.02 (d, *J* = 14.9 Hz, 1H), 3.94 (d, *J* = 9.9 Hz, 1H), 3.87 (d, *J* = 9.9 Hz, 1H), 2.49 (s, 3H). ^{13}**C{1H} NMR** (101 MHz, $CDCl_3$) δ 145.4, 144.4, 141.4, 141.2, 131.7, 129.9, 128.8, 128.5, 128.1, 128.0, 127.8, 127.4, 125.6, 96.0, 76.7, 61.8, 61.5, 52.5, 21.7. HRMS (ESI) calcd for $C_{25}H_{23}O_4N_2INaS$ [M+Na]+ 597.0315; found: 597.0309.

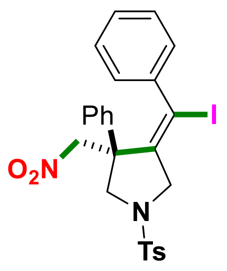

3a (*E*/*Z*= 4:96)

4-(iodo(o-tolyl)methylene)-3-(nitromethyl)-3-phenyl-1-tosylpyrrolidine (3b): The title compound was prepared according to the general procedure and purified by silica gel column chromatography (eluent: hexane/ethyl acetate, 86:14) to afford a mixture of diastereomers isomers (50:50) as a white solid (43 mg, 72% yield); Mp. 183-185 °C; ^{1}**H NMR** (400 MHz, $CDCl_3$) δ 7.79 (dd, J = 17.7, 8.2 Hz, 3.92H), 7.42 (dd, J = 15.5, 8.0 Hz, 4.0H), 7.26 – 7.05 (m, 10.29H), 6.99 (dd, J = 8.1, 3.3 Hz, 3.05H), 6.82 (dd, J = 12.3, 7.1 Hz, 2.97H), 6.67 (t, J = 7.3 Hz, 1.05H), 6.00 (d, J = 7.3 Hz, 1.03H), 4.96 (d, J = 14.6 Hz, 1.02H), 4.62 (d, J = 14.6 Hz, 1.02H), 4.52 (d, J = 14.8 Hz, 1.05H), 4.41 (dd, J = 14.8, 4.3 Hz, 2.01H), 4.26 (d, J = 14.8 Hz, 1.05H), 4.20 (d, J = 10.5 Hz, 1.01H), 4.08 (dd, J = 16.9, 12.6 Hz, 2.07H), 3.96 (d, J = 14.6 Hz, 1.07H), 3.77 (dd, J = 12.6, 10.4 Hz, 2.08H), 2.51 (s, 2.90H), 2.49 (s, 3.00H), 2.15 (s, 3.02H), 1.21 (s, 3.02H). ^{13}**C{1H} NMR** (101 MHz, $CDCl_3$) δ 145.2, 144.5, 144.3, 143.8, 141.0, 139.6, 139.4, 138.5, 136.8, 135.0, 131.8, 131.2, 130.8, 130.7, 130.0, 130.0, 129.3, 129.1, 128.8, 128.6, 128.2, 128.0, 127.8, 127.7, 127.6, 127.2, 125.6, 125.3, 97.8, 96.6, 78.0, 76.3, 62.2, 61.6, 61.6, 61.5, 52.3, 51.7, 21.7, 19.6, 18.5. HRMS (ESI) calcd for $C_{26}H_{25}O_4N_2INaS$ [M+Na]+ 611.0471; found: 611.0471.

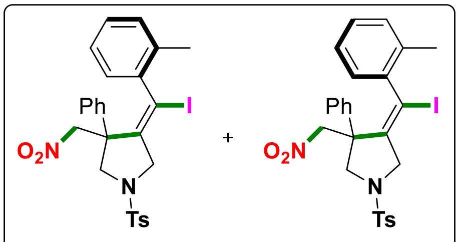

3b (*dr* = 50:50 or 50:50)

4-(iodo(2-methoxyphenyl)methylene)-3-(nitromethyl)-3-phenyl-1-tosylpyrrolidine (3c): The title compound was prepared according to the general procedure and purified by silica gel column chromatography (eluent: hexane/ethyl acetate, 83:17) to afford a mixture of diastereomers isomers (45:55 or 55:45) as a white solid (44 mg, 70% yield); Mp. 208-210 °C; ^{1}**H NMR** (400 MHz, $CDCl_3$) δ 7.77 (d, J = 8.3 Hz, 2.93H), 7.41 (d, J = 8.0 Hz, 3.03H), 7.24 – 7.08 (m, 5.02H), 7.07 – 6.98 (m, 1.53H), 6.98 – 6.92 (m, 2.05H), 6.86 – 6.76 (m, 2.50H), 6.43 – 6.34 (m, 1.53H), 5.96 (dd, J = 7.6, 1.7 Hz, 1.01H), 4.89 (d, J = 14.0 Hz, 0.51H), 4.68 (dd, J = 14.8, 10.0 Hz, 1.53H), 4.43 (d, J = 15.6 Hz, 1.03H), 4.37 (d, J = 14.8 Hz, 1H), 4.21 (d, J = 14.7 Hz, 0.51H), 4.10 – 3.99 (m, 3.08H), 3.90 (dd, J = 23.0, 10.2 Hz, 1.59H), 3.83 (s, 3H), 3.29 (s, 1.37H), 2.50 (s, 1.35H), 2.49 (s, 3H). ^{13}**C{1H} NMR** (101 MHz, $CDCl_3$) δ 155.1, 145.9, 144.6, 144.3, 144.3, 141.1, 138.0, 131.8, 130.6, 130.3, 130.0, 129.5, 129.4, 129.0, 128.6, 128.2, 128.1, 128.0, 127.9, 127.5, 126.9, 125.8, 125.5, 120.2, 120.0, 110.8, 110.6, 94.6, 92.6, 76.7, 75.7, 61.5, 61.3, 61.3, 61.1, 55.3, 54.4, 52.9, 52.1, 21.7. HRMS (ESI) calcd for $C_{26}H_{25}O_5N_2INaS$ [M+Na]+ 627.0421; found: 627. 04215.

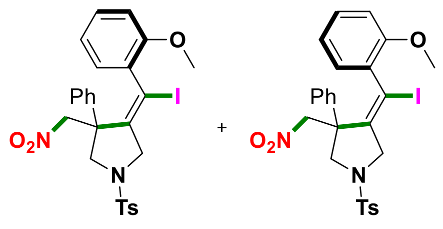

3c (*dr* = 45:55 or 55:45)

4-(iodo(m-tolyl)methylene)-3-(nitromethyl)-3-phenyl-1-tosylpyrrolidine (3d): The title compound was prepared according to the general procedure and purified by silica gel column chromatography (eluent: hexane/ethyl acetate, 86:14) to afford a mixture of E/Z isomers (5:95) as a white solid (42 mg, 71% yield); Mp. 164-166 °C; ^{1}**H NMR** (400 MHz, $CDCl_3$) δ 7.75 (d, J = 8.3 Hz, 2H), 7.41 – 7.37 (m, 2H), 7.25 – 7.18 (m, 3H), 7.00 – 6.90 (m, 4H), 6.55 (brs, 1H), 6.20 (brs, 1H), 4.65 (d, J = 14.6 Hz, 1H), 4.36 (d, J = 8.0 Hz, 1H), 4.32 (d, J = 7.7 Hz, 1H), 4.00 (d, J = 14.9 Hz, 1H), 3.94 (d, J = 10.0 Hz, 1H), 3.86 (d, J = 10.0 Hz, 1H), 2.48 (s, 3H), 2.02 (s, 3H). ^{13}**C{1H} NMR** (101 MHz, $CDCl_3$) δ 145.2, 144.3, 141.5,

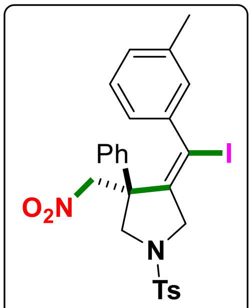

3d (*E*/*Z*= 5:95)

141.2, 137.8, 131.6, 129.9, 129.3, 128.7, 128.0, 127.9, 127.6, 125.5, 124.3, 96.2, 76.7, 61.8, 61.5, 52.3, 21.6, 21.2. HRMS (ESI) calcd for $C_{26}H_{25}O_4N_2INaS$ [M+Na]+ 611.0471; found: 611.0466.

(Z)-4-(iodo(p-tolyl)methylene)-3-(nitromethyl)-3-phenyl-1-tosylpyrrolidine (3e): The title compound was

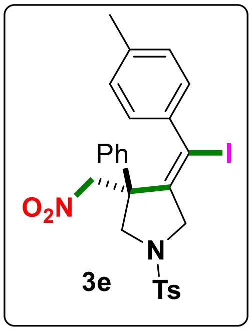

prepared according to the general procedure and purified by silica gel column chromatography (eluent: hexane/ethyl acetate, 85:15) to afford the *Z* isomer as a white solid (46 mg, 76% yield); Mp. 176-178 °C; ^{1}**H NMR** (400 MHz, $CDCl_3$) δ 7.74 (d, *J* = 8.2 Hz, 2H), 7.39 (d, *J* = 8.0 Hz, 2H), 7.26 – 7.20 (m, 3H), 6.99 (dd, *J* = 7.9, 1.7 Hz, 2H), 6.84 (d, *J* = 7.9 Hz, 2H), 6.54 (s, 2H), 4.63 (d, *J* = 14.5 Hz, 1H), 4.36 (d, *J* = 4.8 Hz, 1H), 4.32 (d, *J* = 4.3 Hz, 1H), 3.99 (d, *J* = 13.7 Hz, 1H), 3.96 (d, *J* = 8.5 Hz, 1H), 3.83 (d, *J* = 9.9 Hz, 1H), 2.49 (s, 3H), 2.24 (s, 3H). ^{13}**C{1H} NMR** (101 MHz, $CDCl_3$) δ 145.1, 144.3, 141.3, 138.7, 131.7, 129.9, 128.8, 128.0, 127.8, 127.3, 125.6, 96.4, 76.7, 61.9, 61.5, 52.5, 21.7, 21.2, 15.6, 15.6. HRMS (ESI) calcd for $C_{26}H_{25}O_4N_2INaS$ [M+Na]+ 611.0471; found: 611.0467.

4-(iodo(4-methoxyphenyl)methylene)-3-(nitromethyl)-3-phenyl-1-tosylpyrrolidine (3f): The title compound

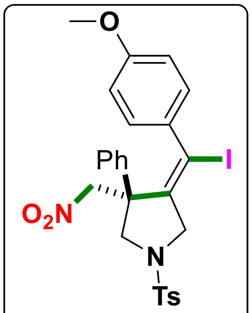

3f (*E*/*Z*= 15:85)

was prepared according to the general procedure and purified by silica gel column chromatography (eluent: hexane/ethyl acetate, 82:18) to afford a mixture of E/Z isomers (15:85) as a white solid (37 mg, 59% yield); Mp. 150-152 °C; ^{1}**H NMR** (400 MHz, $CDCl_3$) δ 7.73 (d, *J* = 8.2 Hz, 2H), 7.38 (d, *J* = 8.0 Hz, 2H), 7.27 – 7.21 (m, 3H), 7.02 – 6.97 (m, 2H), 6.55 (brs, 4H), 4.65 (d, *J* = 14.5 Hz, 1H), 4.36 (d, J = 5.1 Hz, 1H), 4.32 (d, *J* = 5.6 Hz, 1H), 4.01 – 3.92 (m, 2H), 3.84 (d, *J* = 7.7 Hz, 1H), 3.72 (s, 3H), 2.48 (s, 3H). ^{13}**C{1H} NMR** (101 MHz, $CDCl_3$) δ 159.3, 145.3, 144.3, 141.4, 133.8, 131.7, 129.9, 128.9, 128.8, 127.9, 127.8, 125.6, 113.4, 96.5, 76.7, 61.9, 61.5, 55.2, 52.5, 21.6. HRMS (ESI) calcd for $C_{26}H_{25}O_5N_2INaS$ [M+Na]+ 627.0421; found: 627.0425.

(*Z*)-4-((3-bromophenyl)iodomethylene)-3-(nitromethyl)-3-phenyl-1-tosylpyrrolidine (3g): The title compound was prepared according to the general procedure and purified by silica gel column chromatography

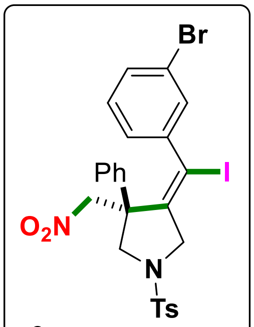

3g (*E*/*Z*= 3:97)

(eluent: hexane/ethyl acetate, 83:17) to afford a mixture of E/Z isomers (86:14) as a white solid (49 mg, 73% yield); Mp. 204-206 °C; ^{1}**H NMR** (400 MHz, $CDCl_3$) δ 7.76 (d, *J* = 8.2 Hz, 2H), 7.41 (d, *J* = 8.0 Hz, 2H), 7.30 – 7.20 (m, 5H), 6.97 (t, *J* = 8.0 Hz, 1H), 6.91 (d, *J* = 7.1 Hz, 2H), 6.70 (brs, 1H), 6.49 (brs, 1H), 4.70 (d, *J* = 14.2 Hz, 1H), 4.43 (d, *J* = 14.2 Hz, 1H), 4.31 (d, *J* = 15.0 Hz, 1H), 4.03 (d, *J* = 15.1 Hz, 1H), 3.93 (dd, *J* = 23.1, 10.1 Hz, 2H), 2.49 (s, 3H). ^{13}**C{1H} NMR** (101 MHz, $CDCl_3$) δ 146.9, 144.5, 143.0, 140.9, 131.6, 131.6, 130.3, 130.0, 129.5, 128.9, 128.1, 128.0, 126.0, 125.3, 121.8, 110.0, 93.5, 76.7, 61.8, 61.6, 52.5, 21.7. HRMS (ESI) calcd for $C_{25}H_{22}O_4N_2BrINaS$ [M+Na]+ 676.9400; found: 676.9395.

(*Z*)-4-((3-chlorophenyl)iodomethylene)-3-(nitromethyl)-3-phenyl-1-tosylpyrrolidine (3h): The title compound was prepared according to the general procedure and purified by silica gel column chromatography (eluent: hexane/ethyl acetate, 83:17) to afford the Z isomer as a white solid (39 mg, 64% yield); Mp. 197-199 °C; ^{1}**H NMR** (400 MHz, $CDCl_3$) δ 7.76 (d, *J* = 8.3 Hz, 2H), 7.41 (d, *J* = 8.1 Hz, 2H), 7.30 – 7.19 (m, 3H), 7.10 – 7.07 (m, 1H), 7.02 (t, *J* = 7.7 Hz, 1H), 6.91 (d, *J* = 7.0 Hz, 2H), 6.63 (brs, 1H), 6.34 (brs, 1H), 4.71 (d, *J* = 14.2 Hz, 1H), 4.43 (d, *J* = 14.2 Hz, 1H), 4.31 (d, *J* = 15.0 Hz, 1H), 4.02 (d, *J* = 15.0 Hz, 1H), 3.96 (d, *J* = 10.1 Hz, 1H), 3.90 (d, *J* = 10.1 Hz, 1H), 2.49 (s, 3H). ^{13}**C{1H} NMR** (101 MHz, $CDCl_3$) δ 146.8, 144.5, 142.7, 140.9, 133.7, 131.5, 130.0, 129.3, 128.9, 128.6, 128.0, 127.4, 125.5, 125.3, 93.7, 76.7, 61.8, 61.5, 52.5, 21.7. HRMS (ESI) calcd for $C_{25}H_{22}O_4N_2ClINaS$ [M+Na]+ 630.9925; found: 630.9920.

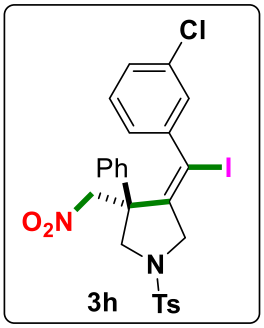

(*Z*)-4-((3-fluorophenyl)iodomethylene)-3-(nitromethyl)-3-phenyl-1-tosylpyrrolidine (3i): The title compound was prepared according to the general procedure and purified by silica gel column chromatography (eluent: hexane/ethyl acetate, 83:17) to afford the Z isomer as a white solid (40 mg, 67% yield); Mp. 172-174 °C; ^{1}**H NMR** (400 MHz, $CDCl_3$) δ 7.75 (d, *J* = 8.3 Hz, 2H), 7.40 (d, *J* = 8.0 Hz, 2H), 7.29 – 7.18 (m, 3H), 7.04 (dd, *J* = 13.9, 7.7 Hz, 1H), 6.95 – 6.90 (m, 2H), 6.81 (tdd, *J* = 8.4, 2.6, 0.8 Hz, 1H), 6.49 (brs, 1H), 6.16 (brs, 1H), 4.71 (d, *J* = 14.2 Hz, 1H), 4.43 (d, *J* = 14.2 Hz, 1H), 4.31 (d, *J* = 15.0 Hz, 1H), 4.03 (d, *J* = 15.0 Hz, 1H), 3.93 (dd, *J* = 21.4, 10.1 Hz, 2H), 2.49 (s, 3H). ^{13}**C{1H} NMR** (101 MHz, $CDCl_3$) δ 162.8, 160.3, 146.5, 144.4, 143.2, 143.1, 140.8, 131.6, 130.0, 129.8, 129.7, 128.9, 128.0, 127.9, 125.4, 123.1, 123.1, 115.7, 115.5, 114.7, 114.4, 93.8, 76.7, 61.8, 61.5, 52.6, 21.6. HRMS (ESI) calcd for $C_{25}H_{22}O_4N_2FINaS$ [M+Na]+ 615.0221; found: 615.0216.

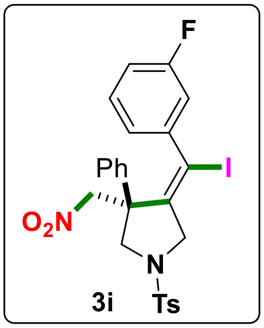

(Z)-4-(iodo(3-(trifluoromethyl)phenyl)methylene)-3-(nitromethyl)-3-phenyl-1-tosylpyrrolidine (3j): The title compound was prepared according to the general procedure and purified by silica gel column chromatography (eluent: hexane/ethyl acetate, 82:18) to afford the Z isomer as a white solid (45 mg, 68% yield); Mp. 207-209 °C; ^{1}**H NMR** (400 MHz, $CDCl_3$) δ 7.76 (d, *J* = 8.3 Hz, 2H), 7.41 (d, *J* = 7.9 Hz, 2H), 7.35 (d, *J* = 7.8 Hz, 1H), 7.26 – 7.16 (m, 4H), 6.96 (brs, 1H), 6.90 – 6.85 (m, 2H), 6.67 (brs, 1H), 4.70 (d, *J* = 14.1 Hz, 1H), 4.40 (d, *J* = 14.1 Hz, 1H), 4.34 (d, *J* = 15.1 Hz, 1H), 4.06 (d, *J* = 15.1 Hz, 1H), 4.00 (d, *J* = 10.0 Hz, 1H), 3.86 (d, *J* = 10.0 Hz, 1H), 2.50 (s, 3H). ^{13}**C{1H} NMR** (101 MHz, $CDCl_3$) δ 147.4, 144.5, 142.0, 140.8, 131.5, 130.8, 130.0, 129.0, 128.7, 128.1, 128.0, 125.2, 125.2, 125.1, 124.2, 124.1, 93.4, 76.7, 62.0, 61.6, 52.6, 21.7. HRMS (ESI) calcd for $C_{26}H_{22}O_4N_2F3INaS$ [M+Na]+ 665.0189; found: 665.0183.

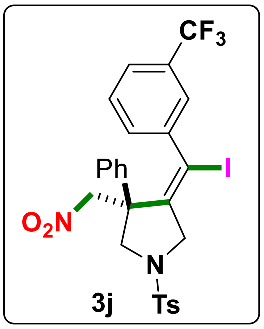

4-(iodo(3-nitrophenyl)methylene)-3-(nitromethyl)-3-phenyl-1-tosylpyrrolidine (3k): The title compound

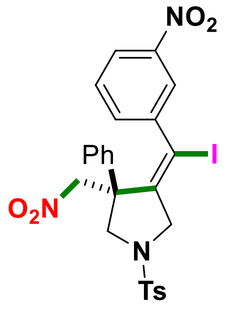
3k (*E*/*Z*= 8:92)

was prepared according to the general procedure and purified by silica gel column chromatography (eluent: hexane/ethyl acetate, 79:21) to afford a mixture of E/Z isomers (6:94) as a white solid (31 mg, 50% yield); Mp. 188-190 °C; ^{1}**H NMR** (400 MHz, $CDCl_3$) δ 7.95 – 7.91 (m, 1H), 7.78 (d, *J* = 8.3 Hz, 2H), 7.43 (d, *J* = 8.0 Hz, 2H), 7.32 – 7.28 (m, 1H), 7.22 – 7.07 (m, 5H), 6.84 (dd, *J* = 7.8, 1.7 Hz, 2H), 4.72 (d, *J* = 13.7 Hz, 1H), 4.50 (d, *J* = 13.7 Hz, 1H), 4.28 (d, *J* = 15.2 Hz, 1H), 4.11 (dd, *J* = 12.7, 2.4 Hz, 2H), 3.84 (d, *J* = 10.2 Hz, 1H), 2.51 (s, 3H). ^{13}**C{1H} NMR** (101 MHz, $CDCl_3$) δ 148.3, 144.6, 142.8, 140.2, 133.2, 131.6, 130.0, 129.2, 129.1, 128.2, 128.1, 125.4, 122.9, 122.5, 92.2, 77.2, 61.5, 53.0, 21.7. HRMS (ESI) calcd for $C_{25}H_{22}O_6N_3INaS$ [M+Na]+ 642.0166; found: 642.0160.

1-(4-(iodo(4-(nitromethyl)-4-phenyl-1-tosylpyrrolidin-3-ylidene)methyl)phenyl)ethan-1-one (3l): The title

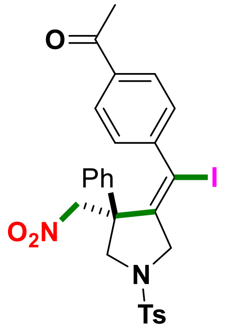
3l (*E*/*Z*= 5:95)

compound was prepared according to the general procedure and purified by silica gel column chromatography (eluent: hexane/ethyl acetate, 80:20) to afford a mixture of E/Z isomers (5:95) as a white solid (39 mg, 62% yield); Mp. 174-176 °C; ^{1}**H NMR** (400 MHz, $CDCl_3$) δ 7.75 (d, *J* = 8.3 Hz, 2H), 7.60 (d, *J* = 8.5 Hz, 2H), 7.40 (d, *J* = 8.0 Hz, 2H), 7.25 (ddd, *J* = 6.1, 2.8, 1.2 Hz, 1H), 7.23 – 7.17 (m, 2H), 6.92 (d, *J* = 7.1 Hz, 2H), 6.71 (brs, 2H), 4.68 (d, *J* = 14.3 Hz, 1H), 4.39 (d, *J* = 14.3 Hz, 1H), 4.35 (d, *J* = 15.1 Hz, 1H), 4.07 (s, 1H), 3.92 (dd, *J* = 21.2, 10.0 Hz, 2H), 2.52 (s, 3H), 2.49 (s, 3H). ^{13}**C{1H} NMR** (101 MHz, $CDCl_3$) δ 196.9, 146.5, 145.8, 144.4, 141.0, 136.4, 131.6, 130.0, 128.9, 128.0, 127.7, 125.5, 93.9, 76.7, 61.9, 61.5, 52.7, 26.5, 21.6. HRMS (ESI) calcd for $C_{27}H_{25}O_5N_2INaS$ [M+Na]+ 639.0421; found: 639.0415.

methyl-4-(iodo(4-(nitromethyl)-4-phenyl-1-tosylpyrrolidin-3-ylidene)methyl)benzoate (3m): The title

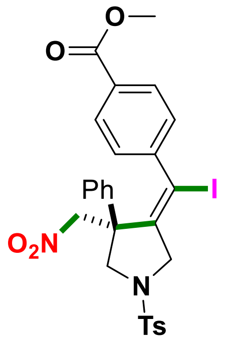
3m (*E*/*Z*= 5:95)

compound was prepared according to the general procedure and purified by silica gel column chromatography (eluent: hexane/ethyl acetate, 78:22) to afford a mixture of E/Z isomers (5:95) as a white solid (38 mg, 60% yield); Mp. 157-159 °C; ^{1}**H NMR** (400 MHz, $CDCl_3$) δ 7.75 (d, *J* = 8.1 Hz, 2H), 7.68 (d, *J* = 7.8 Hz, 2H), 7.40 (d, *J* = 8.0 Hz, 2H), 7.26 – 7.16 (m, 3H), 6.92 (d, *J* = 7.4 Hz, 2H), 6.68 (s, 2H), 4.67 (d, *J* = 14.2 Hz, 1H), 4.38 (d, *J* = 11.4 Hz, 1H), 4.34 (d, *J* = 12.2 Hz, 1H), 4.04 (d, *J* = 15.0 Hz, 1H), 3.94 (dd, *J* = 15.7, 6.7 Hz, 2H), 3.88 (s, 3H), 2.49 (s, 3H), 2.45 (s, 1H). ^{13}**C{1H} NMR** (101 MHz, $CDCl_3$) δ 166.0, 146.5, 145.7, 144.4, 141.0, 131.6, 130.0, 129.8, 129.3, 128.9, 128.0, 127.5, 125.5, 94.0, 76.7, 61.9, 61.6, 52.6, 52.3, 21.7, 21.6. HRMS (ESI) calcd for $C_{27}H_{25}O_6N_2INaS$ [M+Na]+ 655.0370; found: 655.0368.

4-(iodo(4-(nitromethyl)-4-phenyl-1-tosylpyrrolidin-3-ylidene)methyl)benzonitrile (3n): The title compound was prepared according to the general procedure and purified by silica gel column chromatography (eluent: hexane/ethyl acetate, 80:20) to afford a mixture of E/Z isomers (6:94) as a white solid (31 mg, 52% yield); Mp. 155-157 °C; ^{1}**H NMR** (400 MHz, $CDCl_3$) δ 7.76 (d, *J* = 8.2 Hz, 2H), 7.41 (d, *J* = 8.0 Hz, 2H), 7.29 (d, *J* = 8.0 Hz, 2H), 7.26 – 7.17 (m, 3H), 6.89 (d, *J* = 7.2 Hz, 2H), 6.69 (brs, 2H), 4.70 (d, *J* = 14.0 Hz, 1H), 4.42 (d, *J* = 13.9 Hz, 1H), 4.32 (d, *J* = 15.1 Hz, 1H), 4.05 (d, *J* = 15.1 Hz, 1H), 4.01 (d, *J* = 10.1 Hz, 1H), 3.86 (d, *J* = 10.1 Hz, 1H), 2.50 (s, 3H). ^{13}**C{1H} NMR** (101 MHz, $CDCl_3$) δ 147.5, 145.7, 144.6, 140.8, 131.7, 131.5, 130.0, 129.0, 128.2, 128.1, 128.0, 125.5, 117.9, 112.0, 92.6, 76.7, 61.9, 61.6, 52.7, 21.7. HRMS (ESI) calcd for $C_{26}H_{22}O_4N_3INaS$ [M+Na]+ 622.0267; found: 622.0264.

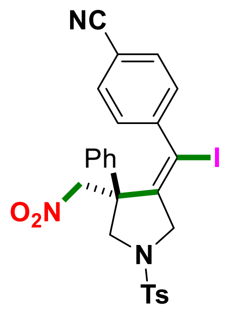

3n (*E*/*Z*= 6:94)

(*Z*)-4-(iodo(thiophen-2-yl)methylene)-3-(nitromethyl)-3-phenyl-1-tosylpyrrolidine (3o): The title compound was prepared according to the general procedure and purified by silica gel column chromatography (eluent: hexane/ethyl acetate, 85:15) to afford the Z isomer as a white solid (47 mg, 81% yield); Mp. 176-178 °C; ^{1}**H NMR** (597 MHz, $CDCl_3$) δ 7.75 (d, *J* = 8.2 Hz, 2H), 7.40 – 7.37 (m, 2H), 7.25 – 7.22 (m, 3H), 7.17 – 7.12 (m, 3H), 6.61 (dd, *J* = 5.1, 3.6 Hz, 1H), 6.33 (dd, *J* = 3.6, 1.2 Hz, 1H), 4.28 (d, *J* = 15.6 Hz, 1H), 3.94 (d, *J* = 15.6 Hz, 1H), 3.76 (q, *J* = 9.7 Hz, 2H), 3.57 (d, *J* = 10.4 Hz, 1H), 3.35 (d, *J* = 10.4 Hz, 1H), 2.48 (s, 3H). ^{13}**C{1H} NMR** (150 MHz, $CDCl_3$) δ 151.0, 144.2, 143.3, 141.9, 131.8, 129.9, 128.6, 128.1, 127.6, 127.4, 127.0, 126.3, 126.1, 86.7, 64.5, 63.0, 54.5, 21.6, 11.9. HRMS (ESI) calcd for $C_{23}H_{21}O_4NINaS_2$ [M+Na]+ 602.9987; found: 602.9985.

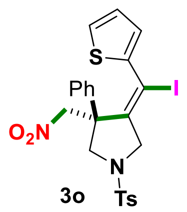

3o

4-(iodo(phenyl)methylene)-3-methyl-3-(nitromethyl)-1-tosylpyrrolidine (3p): The title compound was prepared according to the general procedure and purified by silica gel column chromatography (eluent: hexane/ethyl acetate, 86:17) to afford a mixture of E/Z isomers (10:90) as a white solid (23 mg, 45% yield); Mp. 161-163 °C; ^{1}**H NMR** (399 MHz, $CDCl_3$) δ 7.76 (d, *J* = 8.3 Hz, 2H), 7.41 (d, *J* = 7.9 Hz, 2H), 7.37 – 7.30 (m, 3H), 7.15 (d, *J* = 6.4 Hz, 2H), 4.19 (d, *J* = 12.3 Hz, 1H), 4.02 (dd, *J* = 13.7, 9.2 Hz, 2H), 3.91 (d, *J* = 15.1 Hz, 1H), 3.62 (d, *J* = 9.7 Hz, 1H), 3.23 (d, *J* = 9.7 Hz, 1H), 2.48 (s, 3H), 1.09 (s, 3H). ^{13}**C{1H} NMR** (100 MHz, $CDCl_3$) δ 144.7, 144.3, 142.0, 131.6, 129.9, 129.0, 128.7, 128.0, 128.0, 127.5, 95.1, 79.7, 61.2, 59.3, 47.0, 22.5, 21.6. HRMS (ESI) calcd for $C_{20}H_{21}O_4N_2INaS$ [M+Na]+ 535.0158; found: 535.0158.

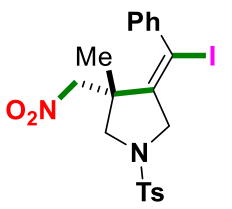

3p (*E*/*Z*= 10:90)

3-(iodo(phenyl)methylene)-4-(nitromethyl)-1-tosylpyrrolidine (3q): The title compound was prepared according to the general procedure and purified by silica gel column chromatography (eluent: hexane/ethyl acetate, 87:13) to afford a mixture of E/Z isomers (5:95**)** as a white solid (21 mg, 41% yield); Mp. 179-181 °C; ^{1}**H NMR** (400 MHz, $CDCl_3$) δ 7.77 (d, J = 8.3 Hz, 2H), 7.42 (d, *J* = 8.0 Hz, 2H), 7.37 – 7.28 (m, 3H), 7.19 – 7.15 (m, 2H), 4.18 (dd, *J* = 13.8, 10.6 Hz, 1H), 4.10 (dd, *J* = 15.4, 1.4 Hz, 1H), 3.90 (dd, *J* = 13.7, 3.3 Hz, 1H), 3.70 (d, *J* = 15.5 Hz, 1H), 3.62 (dd, *J* = 10.4, 2.3 Hz, 1H), 3.56 (brs, 1H), 3.35 (dd, *J* = 10.3, 6.1 Hz, 1H), 2.49 (s, 3H).

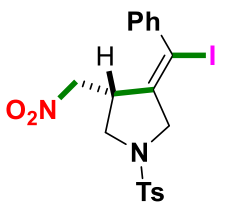

3q (*E*/*Z*= 5:95)

^{13}C{1H} NMR (101 MHz, $CDCl_3$) δ 144.5, 141.4, 141.1, 131.8, 130.0, 129.2, 129.1, 128.8, 128.0, 127.5, 127.3, 95.3, 73.9, 59.2, 52.7, 40.2, 21.6. HRMS (ESI) calcd for $C_{19}H_{19}O_4N_2INaS$ [M+Na]+ 521.0002; found: 520.9997.

4-(iodomethylene)-3-(nitromethyl)-3-phenyl-1-tosylpyrrolidine (3r): The title compound was prepared according to the general procedure and purified by silica gel column chromatography (eluent: hexane/ethyl acetate,

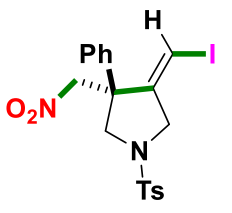

3r (*E*/*Z*= 29:71)

86:14) to afford a mixture of E/Z isomers (29:71) as a white solid (28 mg, 55% yield); Mp. 198-200 °C; **^{1}H NMR** (399 MHz, $CDCl_3$) δ 7.72 (d, *J* = 8.2 Hz, 2H), 7.38 – 7.30 (m, 7H), 6.32 (t, *J* = 2.6 Hz, 1H), 4.89 (d, *J* = 13.2 Hz, 1H), 4.68 (d, *J* = 13.2 Hz, 1H), 4.22 (d, *J* = 10.4 Hz, 1H), 3.87 (d, *J* = 2.7 Hz, 1H), 3.86 – 3.84 (m, 1H), 3.75 (d, *J* = 10.4 Hz, 1H), 2.46 (s, 3H). **^{13}C{1H} NMR** (100 MHz, $CDCl_3$) δ 149.0, 144.3, 136.6, 132.1, 130.0, 129.2, 127.8, 126.1, 79.5, 76.2, 60.9, 58.3, 56.5, 55.0, 21.6. HRMS (ESI) calcd for $C_{19}H_{19}O_4N_2INaS$ [M+Na]+ 521.0002; found: 520.9997.

Mixture of (*E*/*Z*)-4-(1-iodoethylidene)-3-(nitromethyl)-3-phenyl-1-tosylpyrrolidine (3s): The title compound

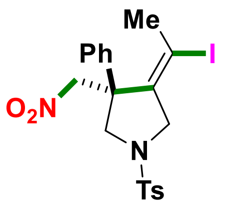

3s (*E*/*Z*= 43:57)

was prepared according to the general procedure and purified by silica gel column chromatography (eluent: hexane/ethyl acetate, 86:14) to afford a mixture of E/Z isomers (43:57) as a white solid (26 mg, 50% yield); Mp. 157-159 °C; **^{1}H NMR** (399 MHz, $CDCl_3$) δ 7.73 – 7.66 (m, 3.61H), 7.39 – 7.27 (m, 9.06H), 7.22 (ddd, *J* = 5.9, 2.1, 0.9 Hz, 2.04H), 7.19 – 7.14 (m, 1.68H), 5.41 (d, *J* = 13.3 Hz, 0.95H), 5.08 (d, *J* = 13.3 Hz, 0.94H), 4.98 (s, 2.04H), 4.19 (dd, *J* = 13.4, 1.2 Hz, 0.92H), 4.07 (d, *J* = 10.1 Hz, 1.07H), 3.97 (q, *J* = 2.0 Hz, 2H), 3.94 (d, *J* = 9.9 Hz, 0.90H), 3.90 (dd, *J* = 13.4, 1.4 Hz, 0.87H), 3.62 (d, *J* = 9.9 Hz, 0.89H), 3.54 (d, *J* = 10.1 Hz, 1.12H), 2.52 (s, 2.30H), 2.46 (s, 2.96H), 2.45 (s, 2.28H), 2.05 (s, 3.0H). **^{13}C{1H} NMR** (100 MHz, $CDCl_3$) δ 144.3, 144.3, 141.2, 140.8, 139.8, 139.1, 131.5, 131.5, 129.9, 129.8, 129.2, 128.9, 128.0, 128.0, 127.9, 127.6, 126.6, 126.0, 97.5, 92.9, 77.0, 76.7, 62.7, 61.6, 61.3, 54.7, 53.8, 53.4, 33.1, 30.9, 21.6. HRMS (ESI) calcd for $C_{20}H_{21}O_4N_2INaS$ [M+Na]+ 535.0158; found: 535.0156.

Mixture of 3-(nitromethyl)-3,5-diphenyl-1-tosyl-1,2,3,4-tetrahydropyridine (4a) and 3-(nitromethyl)-3,5-

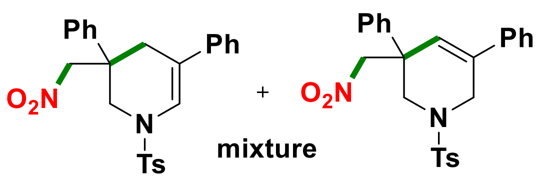

4a:4b (77:23)

diphenyl-1-tosyl-1,2,3,6-tetrahydropyridine (4b): The title compound was prepared according to the general procedure and purified by silica gel column chromatography (eluent: hexane/ethyl acetate, 82:18) to afford a mixture of regio-isomers (77:23) as a white solid (36 mg, 78% yield); Mp. 136-138 °C; **^{1}H NMR** (400 MHz, $CDCl_3$) δ 7.68 (d, *J* = 8.3 Hz, 2H), 7.38 – 7.33 (m, 9H), 7.30 (d, *J* = 8.2 Hz, 3H), 7.15 (s, 1H), 6.48 (s, 1H), 4.62 (s, 2H), 4.08 (dd, *J* = 11.8, 1.4 Hz, 1H), 3.48 (d, *J* = 11.8 Hz, 1H), 3.05 (d, *J* = 17.4 Hz, 1H), 2.90 (d, *J* = 17.2 Hz, 1H), 2.41 (s, 3H). **^{13}C{1H} NMR** (100 MHz, $CDCl_3$) δ 144.4, 138.6, 138.3, 134.4, 130.1, 129.0, 128.7, 127.0, 125.7, 124.9, 121.5, 81.2, 49.7, 40.4, 33.0, 21.5. HRMS (ESI) calcd for $C_{25}H_{24}O_4N_2NaS$ [M+Na]+ 471.1457; found: 471.1453.

5. References

1. (a) Mutra, M. R.; Kudale, V. S.; Li, J.; Tsai, W.-H.; J.-J. Wang. Alkene versus alkyne reactivity in unactivated 1,6-enynes: regio- and chemoselective radical cyclization with chalcogens under metal- and oxidant-free conditions. *Green Chem*. **2020**, *22*, 2288-2300; (b) Mutra, M. R.; Dhandabani, G. K.; Li, J.; Wang, J.-J. Regio- and chemoselective synthesis of nitrogen-containing heterocycles via the oxidative cascade cyclization of unactivated 1,n-enynes. *Chem. Commun*. **2020**, 56, 2051-2054.
2. (a) Wang, Q.; Wang, F.; Yang, X.; Zhou, X.; Li, X. Rh(III)- and Zn(II)-Catalyzed Synthesis of Quinazoline N-Oxides via C–H Amidation–Cyclization of Oximes. *Org. Lett*. **2016**, *18*, 6144–6147
3. Zhu, S.; Cheng, Q.; Yang, H.; Chen, X.; Han, Y.; Yan, C.-G.; Shi, Y.-C.; Hou, H. Three-Component Radical Iodonitrosylative Cyclization of 1,6-Enynes under Metal-Free Conditions. *Org. Lett*. **2021**, 23, 5044–5048.

6. Solvent system and crystallization method for compounds 3a and 4a

Single crystals of compounds **3a** and **4a** suitable for X-ray diffraction analysis were obtained by slow evaporation of acetone solutions of the pure compounds. To facilitate complete dissolution, each sample was refluxed in acetone, and the hot, clear solutions were then allowed to cool slowly to room temperature and left undisturbed under ambient conditions for several days. Crystals of suitable quality for X-ray analysis gradually formed during this process.

The crystallographic measurements were performed on a Rigaku Oxford XtaLab Pro II single-crystal X-ray diffractometer equipped with a microfocus sealed X-ray source and a HyPix detector. Data collection, structure solution, and refinement were carried out using standard crystallographic software packages. All non-hydrogen atoms were refined anisotropically. Hydrogen atoms were placed in idealized positions and refined using a riding model. The thermal ellipsoid plots are displayed at the 50% probability level to illustrate atomic displacement parameters.

The crystal structures unambiguously confirm the molecular connectivity and relative stereochemistry of the compounds. Crystallographic data have been deposited with the Cambridge Crystallographic Data Centre (CCDC) and are accessible free of charge via www.ccdc.cam.ac.uk/data_request/cif under the following accession number: **3a** (CCDC; 2475514) and **4a** (CCDC; 2504228).

6.1. X-ray crystallographic analysis data for compound 3a

checkCIF/PLATON report

Structure factors have been supplied for datablock(s) k11106-jjw-clj4-1_autored

THIS REPORT IS FOR GUIDANCE ONLY. IF USED AS PART OF A REVIEW PROCEDURE FOR PUBLICATION, IT SHOULD NOT REPLACE THE EXPERTISE OF AN EXPERIENCED CRYSTALLOGRAPHIC REFEREE.

No syntax errors found. CIF dictionary Interpreting this report

Datablock: k11106-jjw-clj4-1_autored

Bond precision: C-C = 0.0067 A Wavelength=0.71073

Cell:	a=10.0061(2)	b=8.1301(2)	c=28.6447(5)
	alpha=90	beta=91.056(2)	gamma=90
Temperature:	113 K		

	Calculated	Reported
Volume	2329.87(8)	2329.87(8)
Space group	P 21/c	P 1 21/c 1
Hall group	-P 2ybc	-P 2ybc
Moiety formula	C25 H23 I N2 O4 S [+ solvent]	C25 H23 I N2 O4 S
Sum formula	C25 H23 I N2 O4 S [+ solvent]	C25 H23 I N2 O4 S
Mr	574.41	574.41
Dx,g cm-3	1.638	1.638
Z	4	4
Mu (mm-1)	1.499	1.499
F000	1152.0	1152.0
F000’	1151.04	
h,k,lmax	11,9,34	11,9,34
Nref	4107	4105
Tmin,Tmax	0.799,0.861	0.708,1.000
Tmin’	0.799	

Correction method= # Reported T Limits: Tmin=0.708 Tmax=1.000 AbsCorr = MULTI-SCAN

Data completeness= 1.000 Theta(max)= 24.999

R(reflections)= 0.0455(3583) wR2(reflections)= 0.1231(4105)

S = 1.083 Npar= 299

The following ALERTS were generated. Each ALERT has the format
test-name_ALERT_alert-type_alert-level.
Click on the hyperlinks for more details of the test.

Alert level C

```
RINTA01_ALERT_3_C  The value of Rint is greater than 0.12 Rint
            given        0.126
PLAT906_ALERT_3_C Large K Value in the Analysis of Variance ......      2.131 Check
PLAT911_ALERT_3_C Missing FCF Refl Between Thmin & STh/L=    0.595          2 Report
PLAT971_ALERT_2_C Check Calcd Resid. Dens.  1.01Ang From I1              1.89 eA-3
PLAT971_ALERT_2_C Check Calcd Resid. Dens.  1.04Ang From I1              1.82 eA-3
PLAT972_ALERT_2_C Check Calcd Resid. Dens.  0.84Ang From I1             -1.53 eA-3
PLAT975_ALERT_2_C Check Calcd Resid. Dens.  0.80Ang From O1      .       0.41 eA-3
```

Alert level G

```
PLAT003_ALERT_2_G  Number  of  Uiso  or  Uij  Restrained  non-H  Atoms  ...      31  Report
PLAT020_ALERT_3_G  The  Value  of  Rint  is  Greater  Than  0.12  .........     0.126  Report
PLAT178_ALERT_4_G  The  CIF-Embedded  .res  File  Contains  SIMU  Records       1  Report
PLAT431_ALERT_2_G Short Inter HL..A Contact  I1       ..O3       .       3.30 Ang.
                                           1-x,1-y,1-z  =      3_666 Check
PLAT605_ALERT_4_G Largest Solvent Accessible VOID in the Structure          0 A**3
PLAT793_ALERT_4_G Model has Chirality at C2          (Centro SPGR)          S Verify
PLAT860_ALERT_3_G Number of Least-Squares Restraints .............        768 Note
PLAT909_ALERT_3_G Percentage of I>2sig(I) Data at Theta(Max) Still        72% Note
PLAT910_ALERT_3_G Missing # of FCF Reflection(s) Below Theta(Min).          1 Note
PLAT933_ALERT_2_G Number of HKL-OMIT Records in Embedded .res File          2 Note
PLAT967_ALERT_5_G Note: Two-Theta Cutoff Value in Embedded .res ..       50.0 Degree
PLAT978_ALERT_2_G Number C-C Bonds with Positive Residual Density.          8 Info
```

---

0 **ALERT level A** = Most likely a serious problem - resolve or explain
0 **ALERT level B** = A potentially serious problem, consider carefully
7 **ALERT level C** = Check. Ensure it is not caused by an omission or oversight
12 **ALERT level G** = General information/check it is not something unexpected

0 ALERT type 1 CIF construction/syntax error, inconsistent or missing data
8 ALERT type 2 Indicator that the structure model may be wrong or deficient
7 ALERT type 3 Indicator that the structure quality may be low
3 ALERT type 4 Improvement, methodology, query or suggestion
1 ALERT type 5 Informative message, check

---

checkCIF publication errors

Alert level A

PUBL004_ALERT_1_A The contact author's name and address are missing,

```
            _publ_contact_author_name and _publ_contact_author_address.
PUBL005_ALERT_1_A  _publ_contact_author_email, _publ_contact_author_fax and
            _publ_contact_author_phone are all missing. At
            least one of these should be present.
PUBL006_ALERT_1_A  _publ_requested_journal is missing
            e.g. 'Acta Crystallographica Section C' PUBL008_ALERT_1_A
_publ_section_title is missing. Title of paper.
PUBL009_ALERT_1_A  _publ_author_name is missing. List of author(s) name(s). PUBL010_ALERT_1_A
_publ_author_address is missing. Author(s) address(es). PUBL012_ALERT_1_A  _publ_section_abstract
is missing.
            Abstract of paper in English.
```

7 **ALERT level A** = Data missing that is essential or data in wrong format
0 **ALERT level G** = General alerts. Data that may be required is missing

Publication of your CIF

You should attempt to resolve as many as possible of the alerts in all categories. Often the minor alerts point to easily fixed oversights, errors and omissions in your CIF or refinement strategy, so attention to these fine details can be worthwhile. In order to resolve some of the more serious problems it may be necessary to carry out additional measurements or structure refinements. However, the nature of your study may justify the reported deviations from journal submission requirements and the more serious of these should be commented upon in the discussion or experimental section of a paper or in the "special_details" fields of the CIF. *checkCIF* was carefully designed to identify outliers and unusual parameters, but every test has its limitations and alerts that are not important in a particular case may appear. Conversely, the absence of alerts does not guarantee there are no aspects of the results needing attention. It is up to the individual to critically assess their own results and, if necessary, seek expert advice.

If level A alerts remain, which you believe to be justified deviations, and you intend to submit this CIF for publication in a journal, you should additionally insert an explanation in your CIF using the Validation Reply Form (VRF) below. This will allow your explanation to be considered as part of the review process.

Validation response form

Please find below a validation response form (VRF) that can be filled in and pasted into your CIF.

```
# start Validation Reply Form
_vrf_PUBL004_GLOBAL
;
PROBLEM: The contact author's name and address are missing, RESPONSE: ...
;
_vrf_PUBL005_GLOBAL
;
PROBLEM: _publ_contact_author_email, _publ_contact_author_fax and
RESPONSE: ...
;
_vrf_PUBL006_GLOBAL
```

```
;
PROBLEM: _publ_requested_journal is missing RESPONSE: ...
;
_vrf_PUBL008_GLOBAL
;
PROBLEM: _publ_section_title is missing. Title of paper.
RESPONSE: ...
;
_vrf_PUBL009_GLOBAL
;
PROBLEM: _publ_author_name is missing. List of author(s) name(s).
RESPONSE: ...
;
_vrf_PUBL010_GLOBAL
;
PROBLEM: _publ_author_address is missing. Author(s) address(es). RESPONSE:
...
;
_vrf_PUBL012_GLOBAL
;
PROBLEM: _publ_section_abstract is missing. RESPONSE: ...
;
# end Validation Reply Form
```

If you wish to submit your CIF for publication in Acta Crystallographica Section C or E, you should upload your CIF via the web. If you wish to submit your CIF for publication in IUCrData you should upload your CIF via the web. If your CIF is to form part of a submission to another IUCr journal, you will be asked, either during electronic submission or by the Co-editor handling your paper, to upload your CIF via our web site.

PLATON version of 18/05/2022; check.def file version of 19/01/2022

Figure S1. ORTEP view of X-crystal structure of **3a** (the ellipsoid contour 50% probability levels)

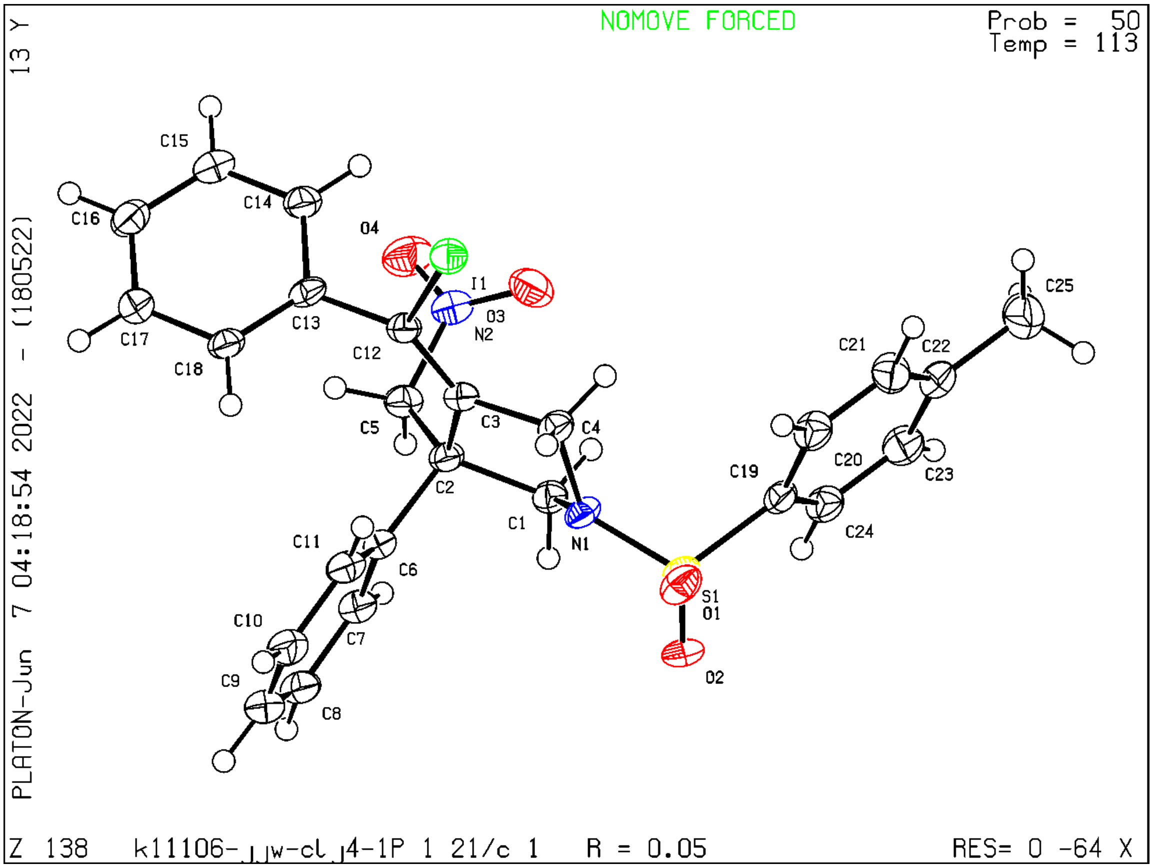

6.2. X-ray crystallographic analysis data for compound 4a

Figure S2. ORTEP view of X-crystal structure of **4a** (the ellipsoid contour 50% probability levels)

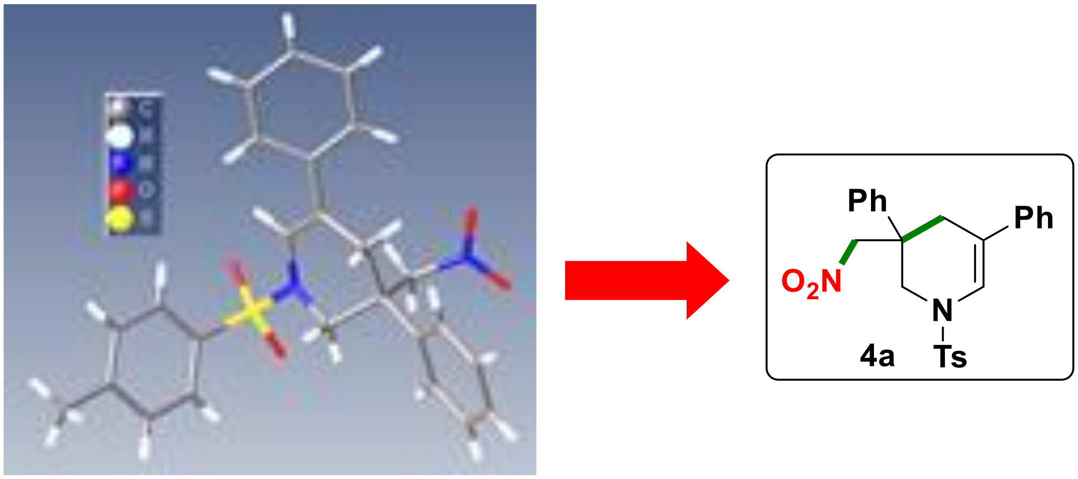

Table S1. Crystal data and structure refinement for **4a**.

Identification code	shelx	
Empirical formula	C25 H24 N2 O4 S	
Formula weight	448.52	
Temperature	200(2) K	
Wavelength	0.71073 Å	
Crystal system	Triclinic	
Space group	P -1	
Unit cell dimensions	a = 9.324(5) Å	a= 76.25(2)°.
	b = 11.440(6) Å	b= 87.56(3)°.
	c = 22.971(12) Å	g = 71.40(2)°.
Volume	2254(2) $Å^3$	
Z	4	
Density (calculated)	1.322 Mg/m^3	
Absorption coefficient	0.178 mm^{-1}	
F(000)	944	
Crystal size	0.390 x 0.350 x 0.200 mm^3	
Theta range for data collection	1.933 to 25.734°.	
Index ranges	-11<=h<=11, -13<=k<=13, -27<=l<=27	
Reflections collected	46242	
Independent reflections	8052 [R(int) = 0.1074]	
Completeness to theta = 25.242°	99.6 %	
Refinement method	Full-matrix least-squares on F^2	
Data / restraints / parameters	8052 / 0 / 581	
Goodness-of-fit on F^2	1.010	
Final R indices [I>2sigma(I)]	R1 = 0.1181, wR2 = 0.3176	
R indices (all data)	R1 = 0.1531, wR2 = 0.3484	
Extinction coefficient	0.034(5)	
Largest diff. peak and hole	0.805 and -0.611 e.$Å^{-3}$	

Table S2. Atomic coordinates (x 10^4) and equivalent isotropic displacement parameters ($Å^2$x 10^3) for d25749a. U(eq) is defined as one third of the trace of the orthogonalized U^{ij} tensor.

	x	y	z	U(eq)
C(1)	6997(10)	2928(7)	3549(4)	42(2)
C(2)	6517(12)	1917(10)	3539(6)	70(3)
C(3)	5126(13)	2146(10)	3253(6)	82(4)
C(4)	4268(11)	3364(9)	2953(5)	54(2)
C(5)	2760(13)	3598(12)	2659(6)	81(4)
C(6)	4800(12)	4326(9)	2957(5)	62(3)
C(7)	6177(13)	4135(9)	3243(5)	64(3)
C(8)	7921(10)	4533(8)	4508(4)	47(2)
C(9)	7472(9)	5042(7)	4993(4)	38(2)
C(10)	7198(9)	6401(8)	4945(4)	39(2)
C(11)	6601(9)	7297(8)	4419(4)	47(2)
C(12)	6338(11)	8570(9)	4367(5)	61(3)
C(13)	6671(11)	8996(9)	4852(6)	62(3)
C(14)	7286(12)	8123(9)	5388(5)	57(2)
C(15)	7556(11)	6831(8)	5428(4)	48(2)
C(16)	7175(12)	4256(8)	5567(4)	48(2)
C(17)	7914(9)	2826(7)	5633(3)	34(2)
C(18)	9668(10)	2433(10)	5675(4)	48(2)
C(19)	7328(8)	2049(7)	6158(4)	35(2)
C(20)	6438(10)	2612(9)	6577(4)	49(2)
C(21)	5985(12)	1802(13)	7075(5)	66(3)
C(22)	6413(13)	536(12)	7141(5)	65(3)
C(23)	7307(12)	5(10)	6729(5)	59(3)
C(24)	7773(9)	736(8)	6242(4)	44(2)
C(25)	7535(11)	2531(8)	5037(4)	45(2)
C(26)	7809(11)	3394(8)	8604(4)	47(2)
C(27)	8755(14)	4048(11)	8713(5)	67(3)
C(28)	10050(14)	3960(13)	8423(5)	72(3)
C(29)	10505(12)	3244(13)	8019(5)	71(3)
C(30)	11973(14)	3112(17)	7699(6)	103(5)
C(31)	9601(13)	2549(10)	7900(5)	63(3)
C(32)	8241(11)	2644(9)	8187(4)	53(2)
C(33)	6869(9)	1075(8)	9549(4)	41(2)
C(34)	7478(9)	71(8)	10018(4)	39(2)
C(35)	7738(9)	-1244(8)	9942(4)	43(2)

C(36)	8307(11)	-1604(11)	9415(5)	58(3)
C(37)	8579(12)	-2848(12)	9366(6)	70(3)
C(38)	8254(12)	-3737(11)	9823(6)	69(3)
C(39)	7671(11)	-3366(9)	10321(5)	59(3)
C(40)	7396(10)	-2134(8)	10400(4)	48(2)
C(41)	7943(11)	263(8)	10594(4)	46(2)
C(42)	7180(9)	1607(7)	10688(3)	37(2)
C(43)	5447(10)	1875(9)	10765(4)	47(2)
C(44)	7877(9)	1842(8)	11213(4)	37(2)
C(45)	8898(11)	886(9)	11623(4)	51(2)
C(46)	9496(13)	1129(11)	12101(5)	66(3)
C(47)	9083(13)	2320(12)	12197(5)	67(3)
C(48)	8021(13)	3293(11)	11815(4)	60(3)
C(49)	7444(10)	3045(9)	11328(4)	49(2)
C(50)	7346(10)	2506(8)	10107(4)	41(2)
N(1)	8214(8)	3240(6)	4534(3)	41(2)
N(2)	10214(9)	2461(9)	6256(4)	58(2)
N(3)	6580(8)	2330(7)	9600(3)	43(2)
N(4)	5108(10)	1125(10)	11341(4)	61(2)
O(1)	9367(7)	1320(6)	4151(3)	55(2)
O(2)	9512(8)	3400(7)	3581(3)	60(2)
O(3)	10016(11)	3503(8)	6374(4)	78(2)
O(4)	10806(9)	1445(8)	6625(3)	72(2)
O(5)	5672(9)	4625(6)	9193(3)	67(2)
O(6)	5141(8)	3191(7)	8630(3)	64(2)
O(7)	5239(10)	19(8)	11403(4)	83(2)
O(8)	4663(12)	1677(10)	11745(4)	93(3)
S(1)	8683(2)	2662(2)	3939(1)	47(1)
S(2)	6138(3)	3476(2)	8990(1)	52(1)

Table S3. Bond lengths [Å] and angles [°] for d25749a.

C(1)-C(2)	1.372(13)
C(1)-C(7)	1.378(13)
C(1)-S(1)	1.747(9)
C(2)-C(3)	1.399(14)
C(2)-H(2)	0.9500
C(3)-C(4)	1.392(14)
C(3)-H(3)	0.9500
C(4)-C(6)	1.346(14)
C(4)-C(5)	1.503(14)
C(5)-H(5A)	0.9800
C(5)-H(5B)	0.9800
C(5)-H(5C)	0.9800
C(6)-C(7)	1.396(15)
C(6)-H(6)	0.9500
C(7)-H(7)	0.9500
C(8)-C(9)	1.368(12)
C(8)-N(1)	1.402(11)
C(8)-H(8)	0.9500
C(9)-C(10)	1.470(11)
C(9)-C(16)	1.479(12)
C(10)-C(11)	1.388(12)
C(10)-C(15)	1.408(12)
C(11)-C(12)	1.374(13)
C(11)-H(11)	0.9500
C(12)-C(13)	1.400(16)
C(12)-H(12)	0.9500
C(13)-C(14)	1.398(16)
C(13)-H(13)	0.9500
C(14)-C(15)	1.399(12)
C(14)-H(14)	0.9500
C(15)-H(15)	0.9500
C(16)-C(17)	1.531(11)
C(16)-H(16A)	0.9900
C(16)-H(16B)	0.9900
C(17)-C(19)	1.513(11)
C(17)-C(18)	1.553(11)
C(17)-C(25)	1.565(11)
C(18)-N(2)	1.459(12)

C(18)-H(18A)	0.9900
C(18)-H(18B)	0.9900
C(19)-C(20)	1.384(12)
C(19)-C(24)	1.392(11)
C(20)-C(21)	1.432(14)
C(20)-H(20)	0.9500
C(21)-C(22)	1.346(16)
C(21)-H(21)	0.9500
C(22)-C(23)	1.358(16)
C(22)-H(22)	0.9500
C(23)-C(24)	1.373(13)
C(23)-H(23)	0.9500
C(24)-H(24)	0.9500
C(25)-N(1)	1.488(11)
C(25)-H(25A)	0.9900
C(25)-H(25B)	0.9900
C(26)-C(27)	1.389(13)
C(26)-C(32)	1.397(13)
C(26)-S(2)	1.748(10)
C(27)-C(28)	1.341(16)
C(27)-H(27)	0.9500
C(28)-C(29)	1.348(17)
C(28)-H(28)	0.9500
C(29)-C(31)	1.403(16)
C(29)-C(30)	1.508(17)
C(30)-H(30A)	0.9800
C(30)-H(30B)	0.9800
C(30)-H(30C)	0.9800
C(31)-C(32)	1.390(15)
C(31)-H(31)	0.9500
C(32)-H(32)	0.9500
C(33)-C(34)	1.360(12)
C(33)-N(3)	1.406(11)
C(33)-H(33)	0.9500
C(34)-C(35)	1.497(12)
C(34)-C(41)	1.496(11)
C(35)-C(40)	1.384(13)
C(35)-C(36)	1.400(12)
C(36)-C(37)	1.395(15)
C(36)-H(36)	0.9500

C(37)-C(38)	1.375(18)
C(37)-H(37)	0.9500
C(38)-C(39)	1.348(16)
C(38)-H(38)	0.9500
C(39)-C(40)	1.404(12)
C(39)-H(39)	0.9500
C(40)-H(40)	0.9500
C(41)-C(42)	1.538(11)
C(41)-H(41A)	0.9900
C(41)-H(41B)	0.9900
C(42)-C(44)	1.510(11)
C(42)-C(50)	1.516(12)
C(42)-C(43)	1.556(12)
C(43)-N(4)	1.478(12)
C(43)-H(43A)	0.9900
C(43)-H(43B)	0.9900
C(44)-C(45)	1.382(11)
C(44)-C(49)	1.391(12)
C(45)-C(46)	1.376(14)
C(45)-H(45)	0.9500
C(46)-C(47)	1.363(16)
C(46)-H(46)	0.9500
C(47)-C(48)	1.377(16)
C(47)-H(47)	0.9500
C(48)-C(49)	1.387(13)
C(48)-H(48)	0.9500
C(49)-H(49)	0.9500
C(50)-N(3)	1.476(10)
C(50)-H(50A)	0.9900
C(50)-H(50B)	0.9900
N(1)-S(1)	1.640(7)
N(2)-O(4)	1.240(11)
N(2)-O(3)	1.241(11)
N(3)-S(2)	1.638(7)
N(4)-O(7)	1.206(12)
N(4)-O(8)	1.229(12)
O(1)-S(1)	1.431(7)
O(2)-S(1)	1.420(7)
O(5)-S(2)	1.430(7)
O(6)-S(2)	1.430(7)

C(2)-C(1)-C(7)	120.3(9)
C(2)-C(1)-S(1)	119.3(7)
C(7)-C(1)-S(1)	120.4(7)
C(1)-C(2)-C(3)	118.9(9)
C(1)-C(2)-H(2)	120.6
C(3)-C(2)-H(2)	120.6
C(4)-C(3)-C(2)	121.3(9)
C(4)-C(3)-H(3)	119.3
C(2)-C(3)-H(3)	119.3
C(6)-C(4)-C(3)	117.9(9)
C(6)-C(4)-C(5)	121.4(9)
C(3)-C(4)-C(5)	120.7(9)
C(4)-C(5)-H(5A)	109.5
C(4)-C(5)-H(5B)	109.5
H(5A)-C(5)-H(5B)	109.5
C(4)-C(5)-H(5C)	109.5
H(5A)-C(5)-H(5C)	109.5
H(5B)-C(5)-H(5C)	109.5
C(4)-C(6)-C(7)	122.4(9)
C(4)-C(6)-H(6)	118.8
C(7)-C(6)-H(6)	118.8
C(1)-C(7)-C(6)	119.0(9)
C(1)-C(7)-H(7)	120.5
C(6)-C(7)-H(7)	120.5
C(9)-C(8)-N(1)	122.5(8)
C(9)-C(8)-H(8)	118.7
N(1)-C(8)-H(8)	118.7
C(8)-C(9)-C(10)	120.5(8)
C(8)-C(9)-C(16)	120.9(7)
C(10)-C(9)-C(16)	118.5(7)
C(11)-C(10)-C(15)	117.9(8)
C(11)-C(10)-C(9)	121.1(8)
C(15)-C(10)-C(9)	121.0(8)
C(12)-C(11)-C(10)	121.8(9)
C(12)-C(11)-H(11)	119.1
C(10)-C(11)-H(11)	119.1
C(11)-C(12)-C(13)	120.1(10)
C(11)-C(12)-H(12)	119.9
C(13)-C(12)-H(12)	119.9

C(14)-C(13)-C(12)	119.9(8)
C(14)-C(13)-H(13)	120.0
C(12)-C(13)-H(13)	120.0
C(13)-C(14)-C(15)	118.8(9)
C(13)-C(14)-H(14)	120.6
C(15)-C(14)-H(14)	120.6
C(14)-C(15)-C(10)	121.4(9)
C(14)-C(15)-H(15)	119.3
C(10)-C(15)-H(15)	119.3
C(9)-C(16)-C(17)	113.9(7)
C(9)-C(16)-H(16A)	108.8
C(17)-C(16)-H(16A)	108.8
C(9)-C(16)-H(16B)	108.8
C(17)-C(16)-H(16B)	108.8
H(16A)-C(16)-H(16B)	107.7
C(19)-C(17)-C(16)	112.4(6)
C(19)-C(17)-C(18)	110.7(7)
C(16)-C(17)-C(18)	111.9(7)
C(19)-C(17)-C(25)	109.6(6)
C(16)-C(17)-C(25)	106.7(6)
C(18)-C(17)-C(25)	105.2(7)
N(2)-C(18)-C(17)	111.8(7)
N(2)-C(18)-H(18A)	109.3
C(17)-C(18)-H(18A)	109.3
N(2)-C(18)-H(18B)	109.3
C(17)-C(18)-H(18B)	109.3
H(18A)-C(18)-H(18B)	107.9
C(20)-C(19)-C(24)	119.4(8)
C(20)-C(19)-C(17)	121.1(7)
C(24)-C(19)-C(17)	119.3(7)
C(19)-C(20)-C(21)	117.6(9)
C(19)-C(20)-H(20)	121.2
C(21)-C(20)-H(20)	121.2
C(22)-C(21)-C(20)	121.7(10)
C(22)-C(21)-H(21)	119.1
C(20)-C(21)-H(21)	119.1
C(21)-C(22)-C(23)	119.5(9)
C(21)-C(22)-H(22)	120.2
C(23)-C(22)-H(22)	120.2
C(22)-C(23)-C(24)	121.2(10)

C(22)-C(23)-H(23)	119.4
C(24)-C(23)-H(23)	119.4
C(23)-C(24)-C(19)	120.5(9)
C(23)-C(24)-H(24)	119.8
C(19)-C(24)-H(24)	119.8
N(1)-C(25)-C(17)	108.4(6)
N(1)-C(25)-H(25A)	110.0
C(17)-C(25)-H(25A)	110.0
N(1)-C(25)-H(25B)	110.0
C(17)-C(25)-H(25B)	110.0
H(25A)-C(25)-H(25B)	108.4
C(27)-C(26)-C(32)	118.5(10)
C(27)-C(26)-S(2)	120.9(8)
C(32)-C(26)-S(2)	120.6(7)
C(28)-C(27)-C(26)	120.5(10)
C(28)-C(27)-H(27)	119.7
C(26)-C(27)-H(27)	119.7
C(27)-C(28)-C(29)	122.7(10)
C(27)-C(28)-H(28)	118.7
C(29)-C(28)-H(28)	118.7
C(28)-C(29)-C(31)	119.0(11)
C(28)-C(29)-C(30)	123.4(12)
C(31)-C(29)-C(30)	117.6(13)
C(29)-C(30)-H(30A)	109.5
C(29)-C(30)-H(30B)	109.5
H(30A)-C(30)-H(30B)	109.5
C(29)-C(30)-H(30C)	109.5
H(30A)-C(30)-H(30C)	109.5
H(30B)-C(30)-H(30C)	109.5
C(32)-C(31)-C(29)	119.3(11)
C(32)-C(31)-H(31)	120.4
C(29)-C(31)-H(31)	120.4
C(31)-C(32)-C(26)	120.0(10)
C(31)-C(32)-H(32)	120.0
C(26)-C(32)-H(32)	120.0
C(34)-C(33)-N(3)	121.1(7)
C(34)-C(33)-H(33)	119.4
N(3)-C(33)-H(33)	119.4
C(33)-C(34)-C(35)	119.0(7)
C(33)-C(34)-C(41)	121.0(7)

C(35)-C(34)-C(41)	120.0(7)
C(40)-C(35)-C(36)	118.5(9)
C(40)-C(35)-C(34)	119.6(8)
C(36)-C(35)-C(34)	121.9(9)
C(37)-C(36)-C(35)	120.4(11)
C(37)-C(36)-H(36)	119.8
C(35)-C(36)-H(36)	119.8
C(38)-C(37)-C(36)	121.2(10)
C(38)-C(37)-H(37)	119.4
C(36)-C(37)-H(37)	119.4
C(39)-C(38)-C(37)	117.6(10)
C(39)-C(38)-H(38)	121.2
C(37)-C(38)-H(38)	121.2
C(38)-C(39)-C(40)	123.8(11)
C(38)-C(39)-H(39)	118.1
C(40)-C(39)-H(39)	118.1
C(35)-C(40)-C(39)	118.5(9)
C(35)-C(40)-H(40)	120.7
C(39)-C(40)-H(40)	120.7
C(34)-C(41)-C(42)	114.0(7)
C(34)-C(41)-H(41A)	108.7
C(42)-C(41)-H(41A)	108.7
C(34)-C(41)-H(41B)	108.7
C(42)-C(41)-H(41B)	108.7
H(41A)-C(41)-H(41B)	107.6
C(44)-C(42)-C(50)	111.2(7)
C(44)-C(42)-C(41)	112.6(6)
C(50)-C(42)-C(41)	105.7(7)
C(44)-C(42)-C(43)	110.1(7)
C(50)-C(42)-C(43)	105.9(7)
C(41)-C(42)-C(43)	111.0(7)
N(4)-C(43)-C(42)	112.0(7)
N(4)-C(43)-H(43A)	109.2
C(42)-C(43)-H(43A)	109.2
N(4)-C(43)-H(43B)	109.2
C(42)-C(43)-H(43B)	109.2
H(43A)-C(43)-H(43B)	107.9
C(45)-C(44)-C(49)	116.2(8)
C(45)-C(44)-C(42)	123.0(7)
C(49)-C(44)-C(42)	120.7(7)

C(46)-C(45)-C(44)	121.5(9)
C(46)-C(45)-H(45)	119.2
C(44)-C(45)-H(45)	119.2
C(47)-C(46)-C(45)	121.2(9)
C(47)-C(46)-H(46)	119.4
C(45)-C(46)-H(46)	119.4
C(46)-C(47)-C(48)	119.3(9)
C(46)-C(47)-H(47)	120.4
C(48)-C(47)-H(47)	120.4
C(47)-C(48)-C(49)	119.1(10)
C(47)-C(48)-H(48)	120.5
C(49)-C(48)-H(48)	120.5
C(48)-C(49)-C(44)	122.6(9)
C(48)-C(49)-H(49)	118.7
C(44)-C(49)-H(49)	118.7
N(3)-C(50)-C(42)	111.1(7)
N(3)-C(50)-H(50A)	109.4
C(42)-C(50)-H(50A)	109.4
N(3)-C(50)-H(50B)	109.4
C(42)-C(50)-H(50B)	109.4
H(50A)-C(50)-H(50B)	108.0
C(8)-N(1)-C(25)	116.1(7)
C(8)-N(1)-S(1)	121.6(6)
C(25)-N(1)-S(1)	118.4(5)
O(4)-N(2)-O(3)	121.9(9)
O(4)-N(2)-C(18)	119.1(9)
O(3)-N(2)-C(18)	118.9(8)
C(33)-N(3)-C(50)	116.9(6)
C(33)-N(3)-S(2)	118.5(6)
C(50)-N(3)-S(2)	118.5(6)
O(7)-N(4)-O(8)	121.8(9)
O(7)-N(4)-C(43)	120.6(9)
O(8)-N(4)-C(43)	117.6(9)
O(2)-S(1)-O(1)	120.0(4)
O(2)-S(1)-N(1)	106.3(4)
O(1)-S(1)-N(1)	106.7(4)
O(2)-S(1)-C(1)	108.5(4)
O(1)-S(1)-C(1)	107.9(4)
N(1)-S(1)-C(1)	106.7(4)
O(5)-S(2)-O(6)	121.0(5)

O(5)-S(2)-N(3)	105.4(4)
O(6)-S(2)-N(3)	107.1(4)
O(5)-S(2)-C(26)	108.4(5)
O(6)-S(2)-C(26)	107.4(4)
N(3)-S(2)-C(26)	106.7(4)

Table S4. Anisotropic displacement parameters ($Å^2$x 10^3)for d25749a. The anisotropic displacement factor exponent takes the form: $-2p^2[h^2a^{*2}U^{11} + ... + 2 h k a^* b^* U^{12}]$

	U^{11}	U^{22}	U^{33}	U^{23}	U^{13}	U^{12}
C(1)	46(5)	29(4)	51(5)	-15(4)	-4(4)	-5(3)
C(2)	58(6)	47(6)	97(8)	9(6)	-25(6)	-22(5)
C(3)	73(7)	48(6)	121(10)	23(6)	-41(7)	-36(5)
C(4)	53(5)	50(6)	57(6)	-6(4)	-5(4)	-16(4)
C(5)	59(7)	82(8)	87(8)	4(7)	-25(6)	-18(6)
C(6)	64(6)	30(5)	83(7)	-12(5)	-14(5)	-2(4)
C(7)	87(7)	33(5)	76(7)	-22(5)	-16(6)	-18(5)
C(8)	52(5)	43(5)	49(5)	-18(4)	2(4)	-14(4)
C(9)	49(5)	26(4)	43(4)	-14(3)	7(4)	-13(3)
C(10)	33(4)	40(5)	50(5)	-17(4)	6(3)	-14(3)
C(11)	40(4)	30(4)	67(6)	-3(4)	-4(4)	-11(3)
C(12)	43(5)	45(5)	87(7)	1(5)	-7(5)	-16(4)
C(13)	55(6)	25(4)	104(8)	-20(5)	18(6)	-9(4)
C(14)	67(6)	38(5)	74(7)	-23(5)	9(5)	-21(4)
C(15)	60(5)	31(4)	55(5)	-16(4)	9(4)	-16(4)
C(16)	76(6)	32(4)	40(4)	-11(4)	4(4)	-20(4)
C(17)	37(4)	24(4)	40(4)	-11(3)	4(3)	-7(3)
C(18)	40(5)	66(6)	48(5)	-17(4)	8(4)	-27(4)
C(19)	27(4)	33(4)	45(4)	-10(3)	4(3)	-10(3)
C(20)	51(5)	41(5)	53(5)	-10(4)	7(4)	-15(4)
C(21)	53(6)	102(9)	51(6)	-23(6)	13(5)	-31(6)
C(22)	70(7)	77(8)	54(6)	3(5)	5(5)	-45(6)
C(23)	69(6)	46(5)	63(6)	4(5)	-7(5)	-32(5)
C(24)	42(4)	32(4)	55(5)	-6(4)	5(4)	-11(3)
C(25)	50(5)	35(4)	50(5)	-9(4)	-1(4)	-16(4)
C(26)	58(5)	36(5)	40(4)	3(4)	-13(4)	-13(4)
C(27)	99(9)	75(7)	53(6)	-24(5)	5(6)	-58(7)
C(28)	76(7)	99(9)	57(6)	-11(6)	0(6)	-57(7)
C(29)	52(6)	93(9)	55(6)	10(6)	-12(5)	-25(6)
C(30)	50(6)	157(15)	82(9)	5(9)	-9(6)	-28(8)
C(31)	70(7)	46(6)	62(6)	-6(5)	12(5)	-10(5)
C(32)	62(6)	40(5)	52(5)	-2(4)	0(4)	-16(4)
C(33)	42(4)	48(5)	38(4)	-17(4)	4(3)	-16(4)
C(34)	34(4)	41(5)	44(4)	-15(4)	2(3)	-13(3)
C(35)	29(4)	43(5)	55(5)	-21(4)	-4(4)	-2(3)

C(36)	47(5)	79(7)	64(6)	-42(5)	12(4)	-26(5)
C(37)	48(6)	85(8)	90(8)	-63(7)	0(5)	-5(5)
C(38)	54(6)	53(6)	101(9)	-36(6)	-7(6)	-7(5)
C(39)	57(6)	39(5)	79(7)	-20(5)	-10(5)	-8(4)
C(40)	51(5)	41(5)	51(5)	-20(4)	-10(4)	-7(4)
C(41)	62(5)	27(4)	48(5)	-13(4)	-6(4)	-8(4)
C(42)	45(4)	28(4)	37(4)	-12(3)	-2(3)	-7(3)
C(43)	41(5)	54(5)	41(4)	-5(4)	-7(4)	-13(4)
C(44)	36(4)	34(4)	40(4)	-12(3)	4(3)	-7(3)
C(45)	52(5)	45(5)	42(5)	-7(4)	-12(4)	3(4)
C(46)	60(6)	73(7)	50(6)	-12(5)	-19(5)	-2(5)
C(47)	73(7)	94(9)	47(6)	-19(6)	-5(5)	-43(6)
C(48)	74(7)	67(7)	55(6)	-30(5)	9(5)	-36(6)
C(49)	51(5)	46(5)	58(5)	-19(4)	-4(4)	-21(4)
C(50)	43(4)	40(5)	39(4)	-10(4)	-3(3)	-12(4)
N(1)	51(4)	35(4)	43(4)	-15(3)	8(3)	-19(3)
N(2)	40(4)	66(6)	66(5)	10(4)	-8(4)	-30(4)
N(3)	48(4)	39(4)	36(4)	-3(3)	-8(3)	-11(3)
N(4)	60(5)	79(7)	50(5)	-15(4)	13(4)	-35(5)
O(1)	56(4)	40(3)	64(4)	-24(3)	-6(3)	3(3)
O(2)	70(4)	77(5)	57(4)	-40(4)	29(3)	-43(4)
O(3)	116(7)	73(5)	64(5)	-18(4)	-8(4)	-54(5)
O(4)	75(5)	77(5)	64(4)	-7(4)	-12(4)	-27(4)
O(5)	75(5)	44(4)	58(4)	-7(3)	-3(4)	9(3)
O(6)	47(4)	81(5)	51(4)	6(3)	-20(3)	-13(3)
O(7)	95(6)	69(6)	87(6)	0(4)	17(5)	-46(5)
O(8)	117(7)	121(8)	71(5)	-41(5)	44(5)	-69(6)
S(1)	44(1)	53(1)	49(1)	-25(1)	6(1)	-13(1)
S(2)	51(1)	46(1)	46(1)	-1(1)	-7(1)	-4(1)

Table S5. Hydrogen coordinates (x 10^4) and isotropic displacement parameters (Å^{2}x 10^3) for d25749a.

	x	y	z	U(eq)
H(2)	7119	1076	3723	84
H(3)	4760	1456	3263	99
H(5A)	2499	4398	2351	121
H(5B)	1982	3651	2961	121
H(5C)	2815	2899	2472	121
H(6)	4219	5164	2759	74
H(7)	6543	4828	3226	76
H(8)	8038	5076	4138	56
H(11)	6369	7023	4085	57
H(12)	5929	9162	4001	73
H(13)	6480	9877	4816	74
H(14)	7517	8403	5720	69
H(15)	7991	6231	5789	57
H(16A)	6067	4440	5602	58
H(16B)	7550	4502	5901	58
H(18A)	10104	1565	5610	58
H(18B)	10017	3017	5354	58
H(20)	6138	3504	6536	58
H(21)	5366	2167	7367	80
H(22)	6093	18	7474	78
H(23)	7615	-890	6778	71
H(24)	8403	344	5961	53
H(25A)	7955	1608	5062	54
H(25B)	6424	2795	4969	54
H(27)	8483	4561	8995	80
H(28)	10672	4421	8505	86
H(30A)	11767	3639	7288	155
H(30B)	12461	2222	7687	155
H(30C)	12644	3390	7912	155
H(31)	9916	2019	7625	75
H(32)	7604	2199	8100	63
H(33)	6636	927	9180	49
H(36)	8510	-998	9089	69
H(37)	8997	-3085	9010	85

H(38)	8435	-4583	9789	82
H(39)	7432	-3972	10636	70
H(40)	6985	-1917	10760	57
H(41A)	9054	80	10606	55
H(41B)	7697	-356	10931	55
H(43A)	4957	2790	10745	56
H(43B)	5017	1668	10431	56
H(45)	9193	42	11573	61
H(46)	10210	452	12370	79
H(47)	9522	2478	12524	80
H(48)	7688	4123	11884	72
H(49)	6725	3723	11062	59
H(50A)	6905	3393	10145	49
H(50B)	8435	2352	10026	49

Solvent CDCl$_3$
Spectrometer Frequency 400.28
Nucleus ^{1}H

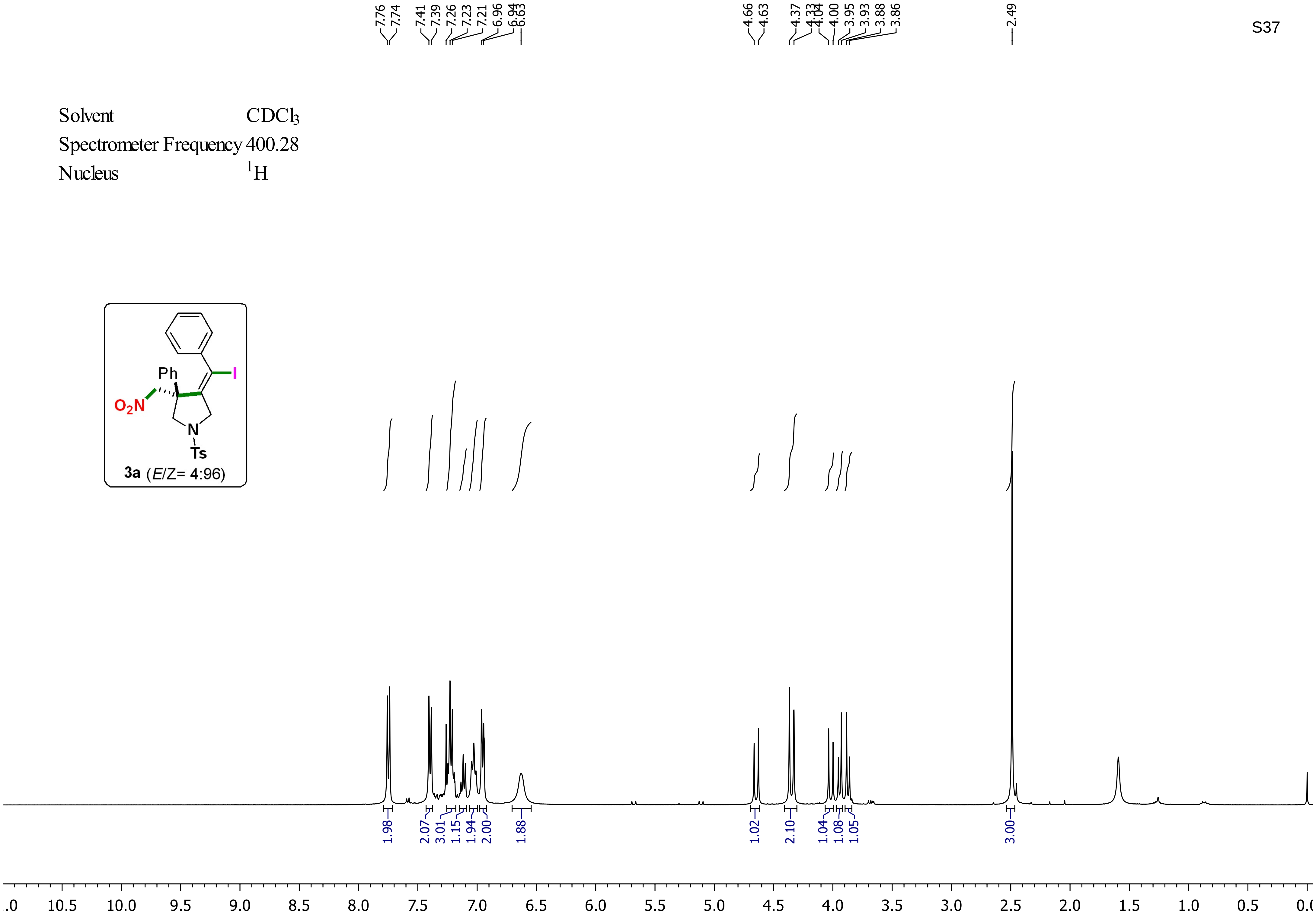

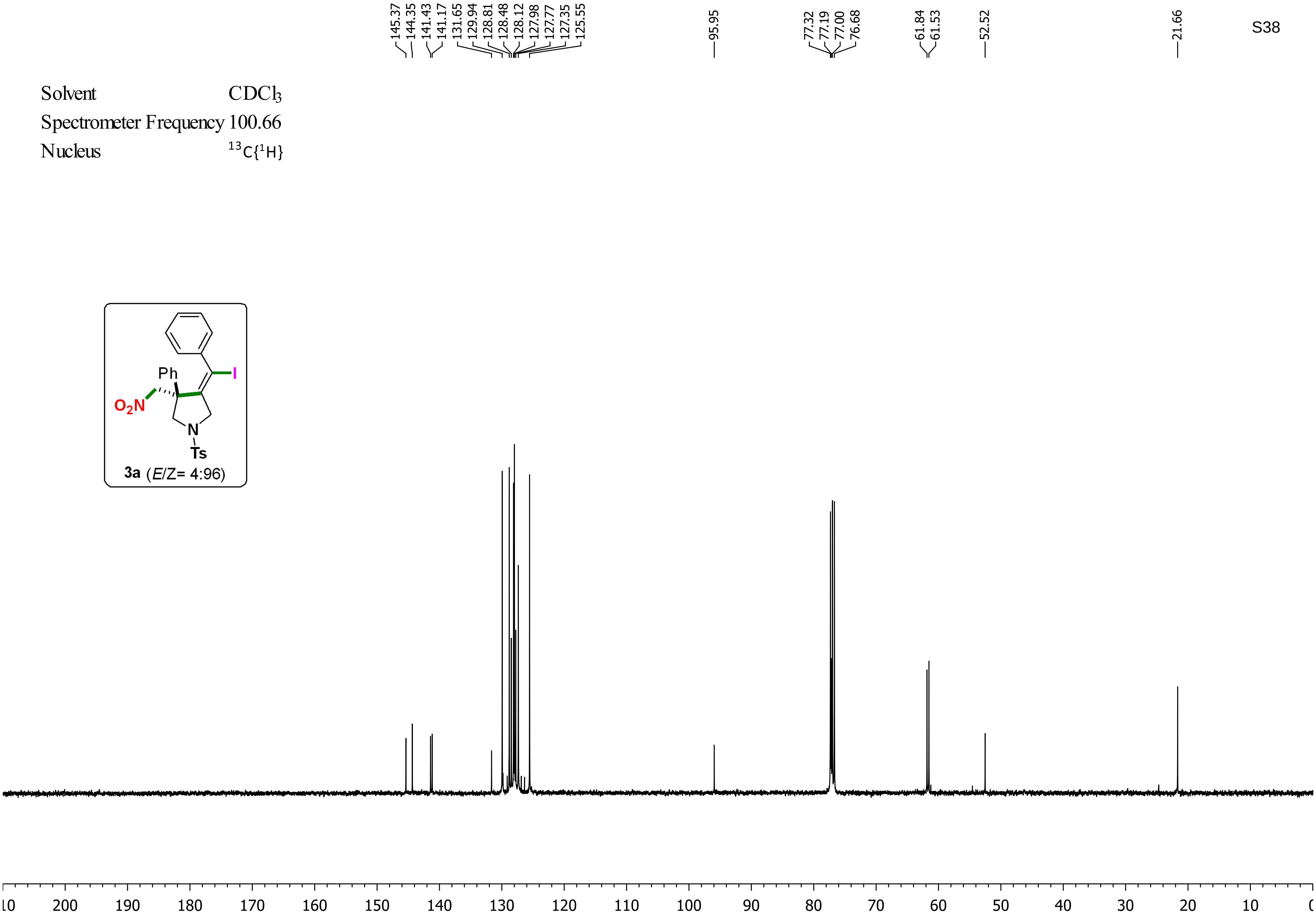

145.37
144.35
141.43
141.17
131.65
129.94
128.81
128.48
128.12
127.98
127.77
127.35
125.55
95.95
77.32
77.19
77.00
76.68
61.84
61.53
52.52
21.66
Solvent CDCl3
Spectrometer Frequency 100.66
Nucleus 13C{1H}
200 190 180 170 160 150 140 130 120 110 100 90 80 70 60 50 40 30 20 10
Ph
O2N
N
Ts
I

3a (*E*/*Z*= 4:96)

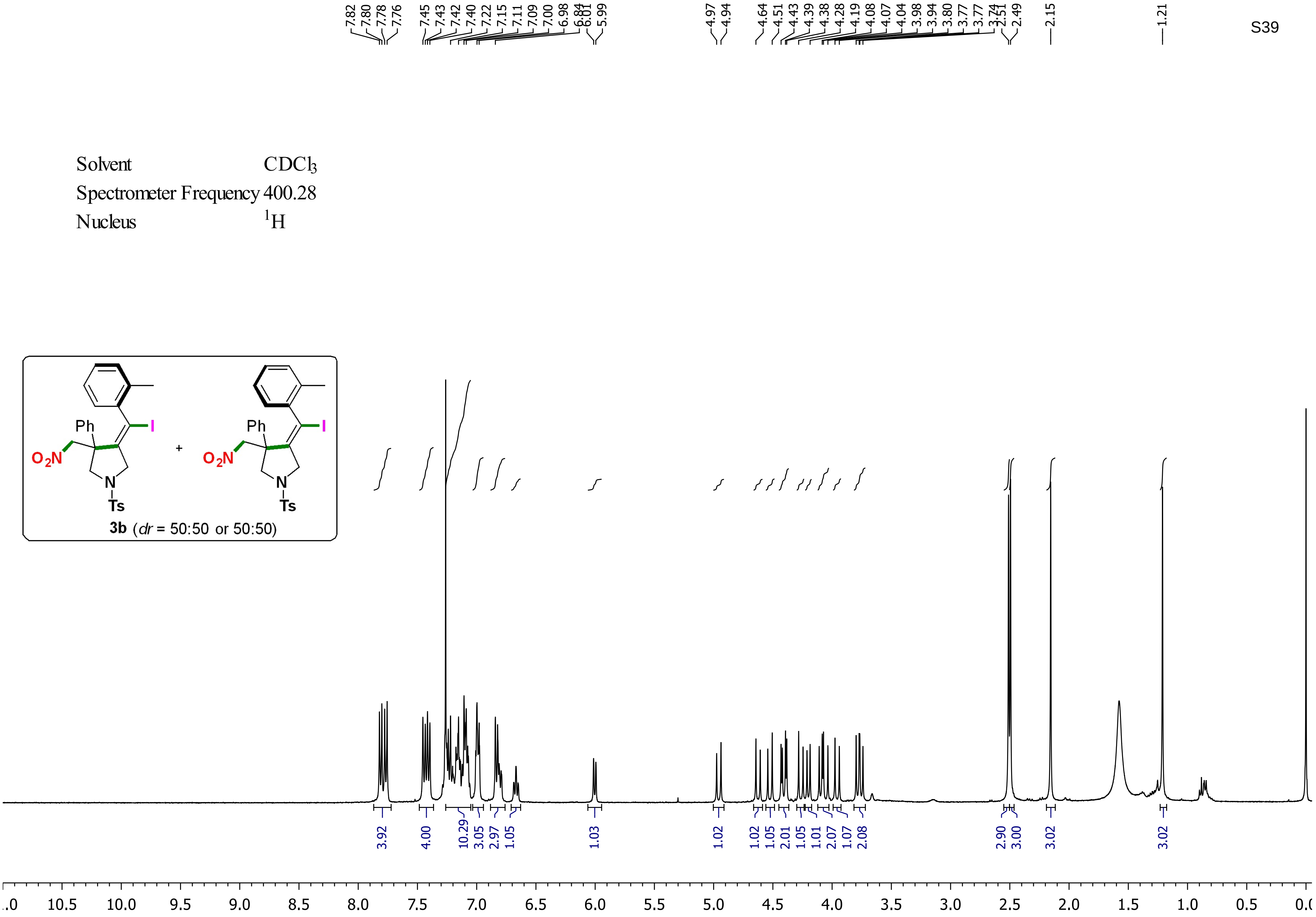

3b (*dr* = 50:50 or 50:50)

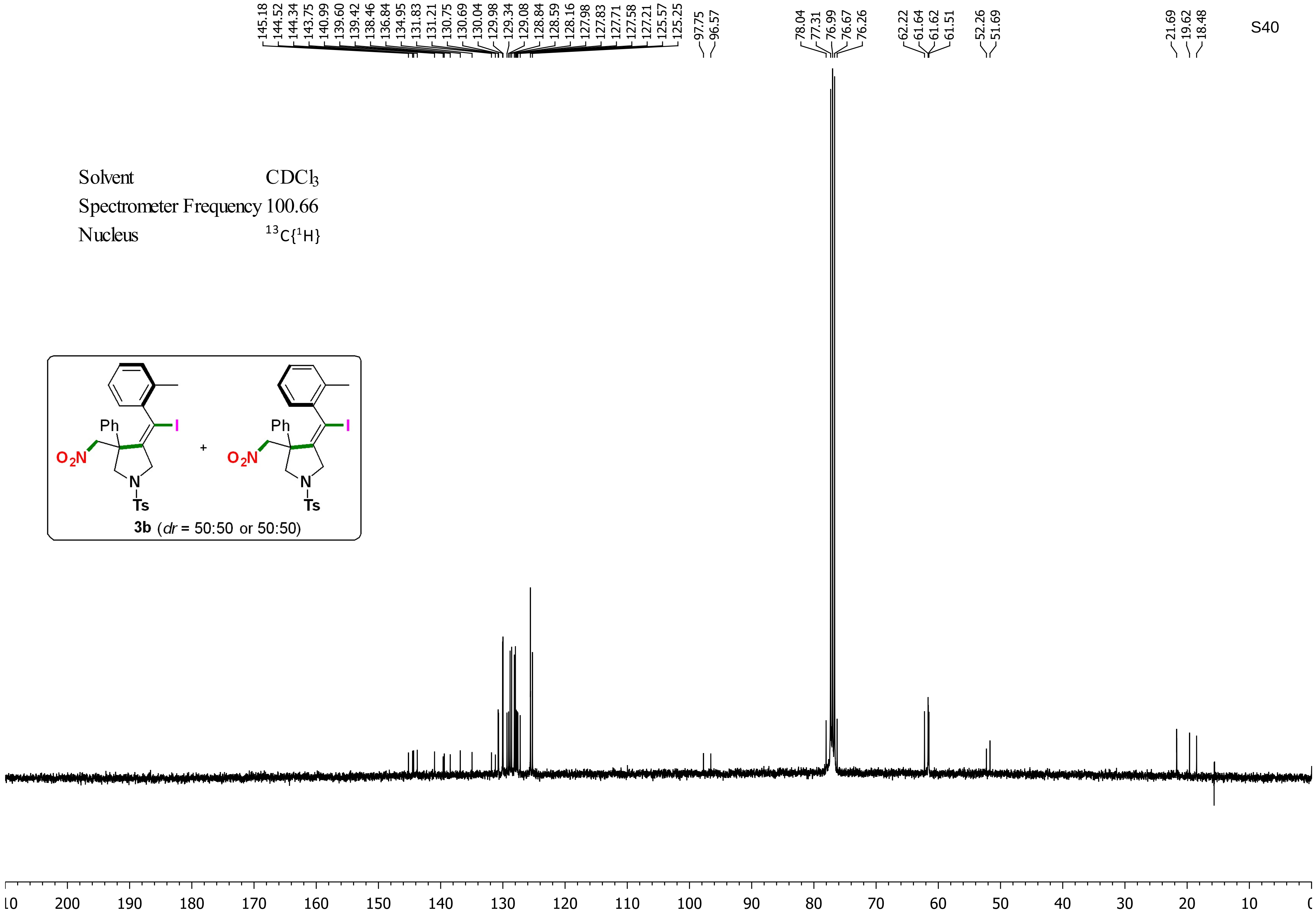
145.18 144.52 144.34 143.75 140.99 139.60 139.42 138.46 136.84 134.95 131.83 131.21 130.75 130.69 130.04 129.98 129.34 129.08 128.84 128.59 128.16 127.98 127.83 127.71 127.58 127.21 125.57 125.25
97.75 96.57
78.04 77.31 76.99 76.67 76.26
62.22 61.64 61.62 61.51
52.26 51.69
21.69 19.62 18.48
Solvent CDCl3
Spectrometer Frequency 100.66
Nucleus 13C{1H}
Ph
O2N
N
Ts
I
+
3b (dr = 50:50 or 50:50)
10 200 190 180 170 160 150 140 130 120 110 100 90 80 70 60 50 40 30 20 10 0

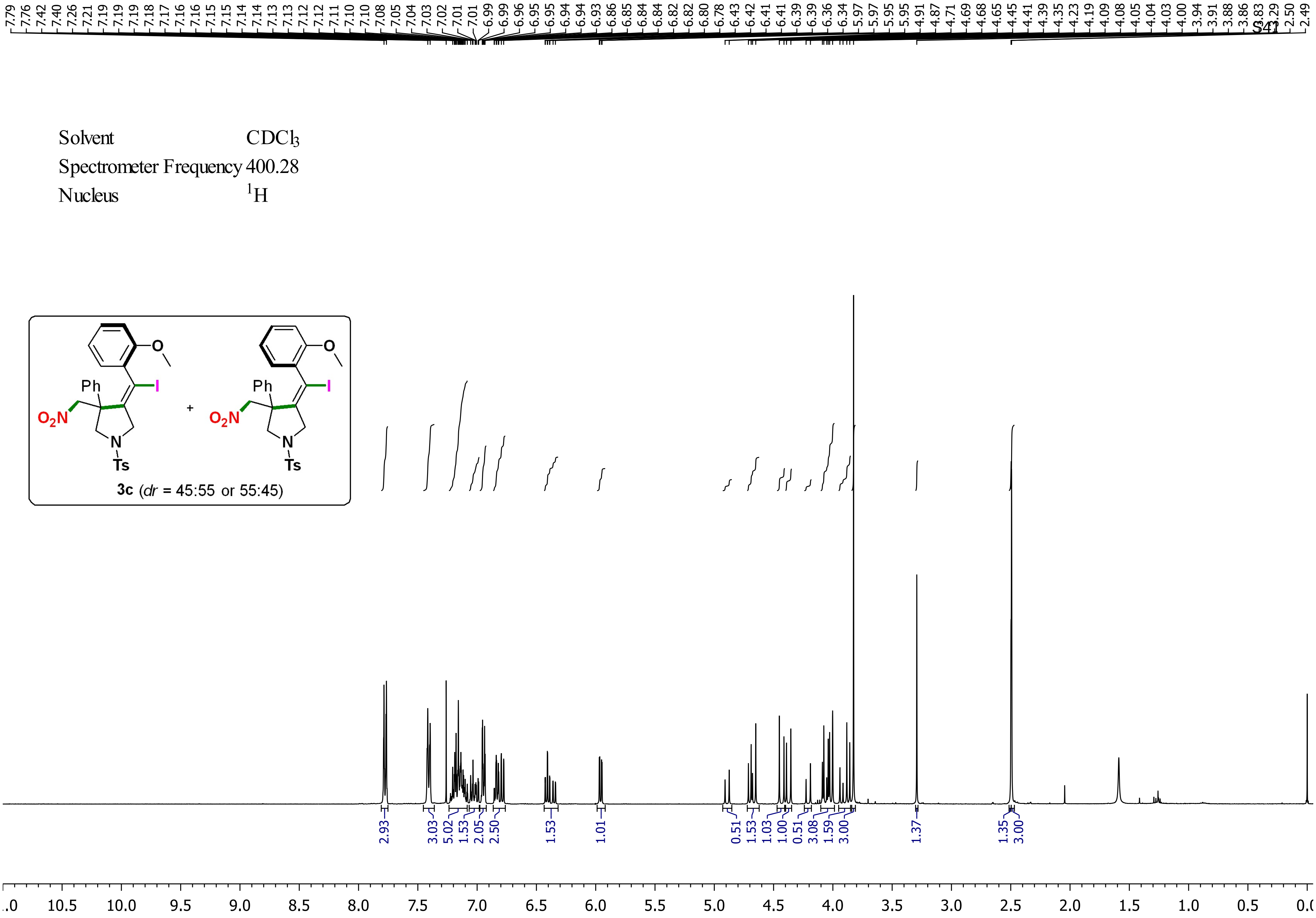

3c (*dr* = 45:55 or 55:45)

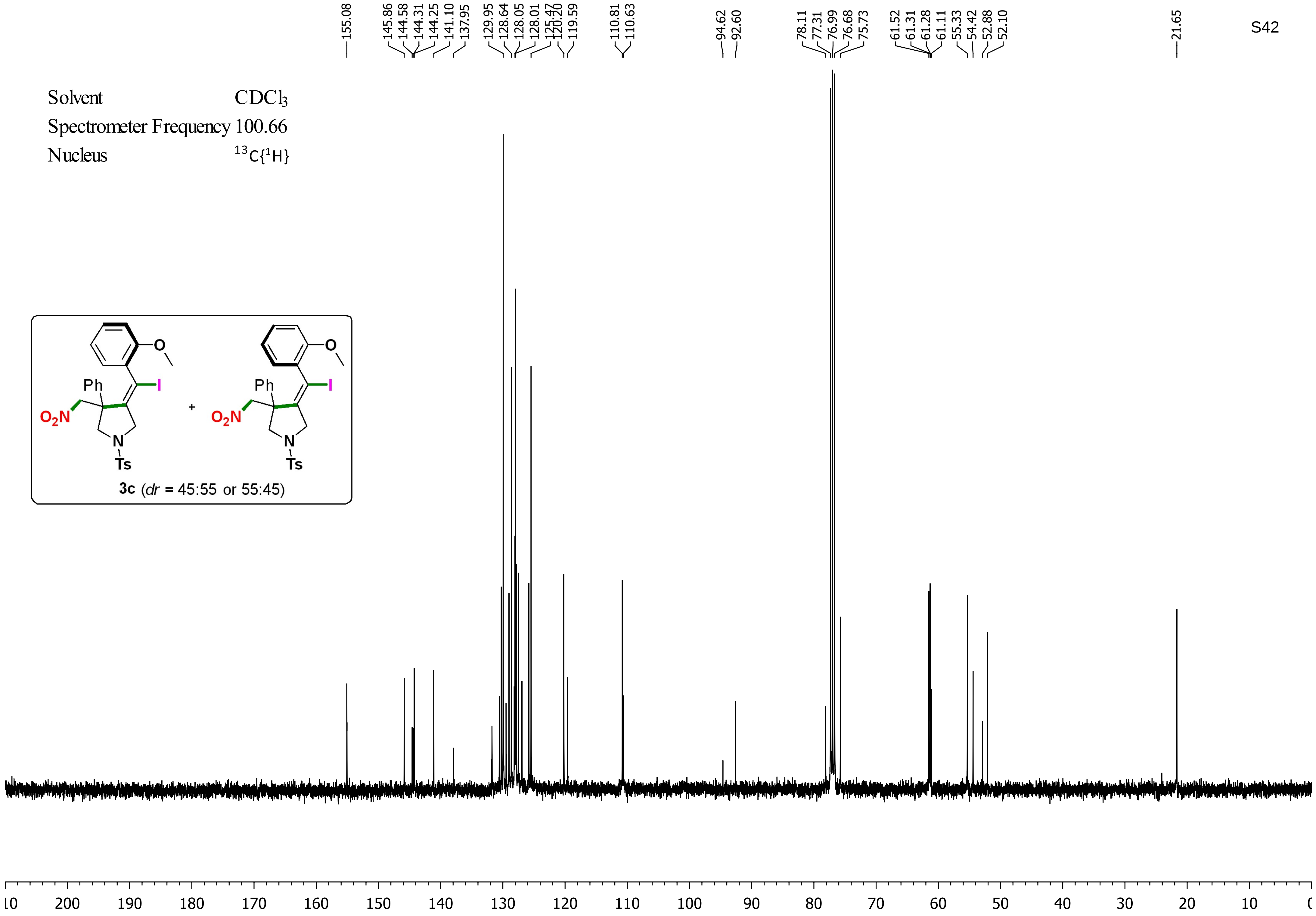
155.08
145.86
144.58
144.31
144.25
141.10
137.95
129.95
128.64
128.05
128.01
125.47
120.20
119.59
110.81
110.63
94.62
92.60
78.11
77.31
76.99
76.68
75.73
61.52
61.31
61.28
61.11
55.33
54.42
52.88
52.10
21.65
Solvent CDCl3
Spectrometer Frequency 100.66
Nucleus 13C{1H}
Ph
O
I
O2N
N
Ts
+
3c (dr = 45:55 or 55:45)
200
190
180
170
160
150
140
130
120
110
100
90
80
70
60
50
40
30
20
10

Solvent CDCl$_3$

Spectrometer Frequency 400.40

Nucleus ^{1}H

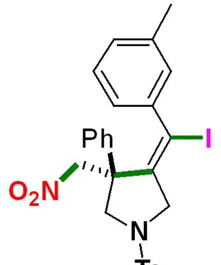

3d (*E*/*Z*= 5:95)

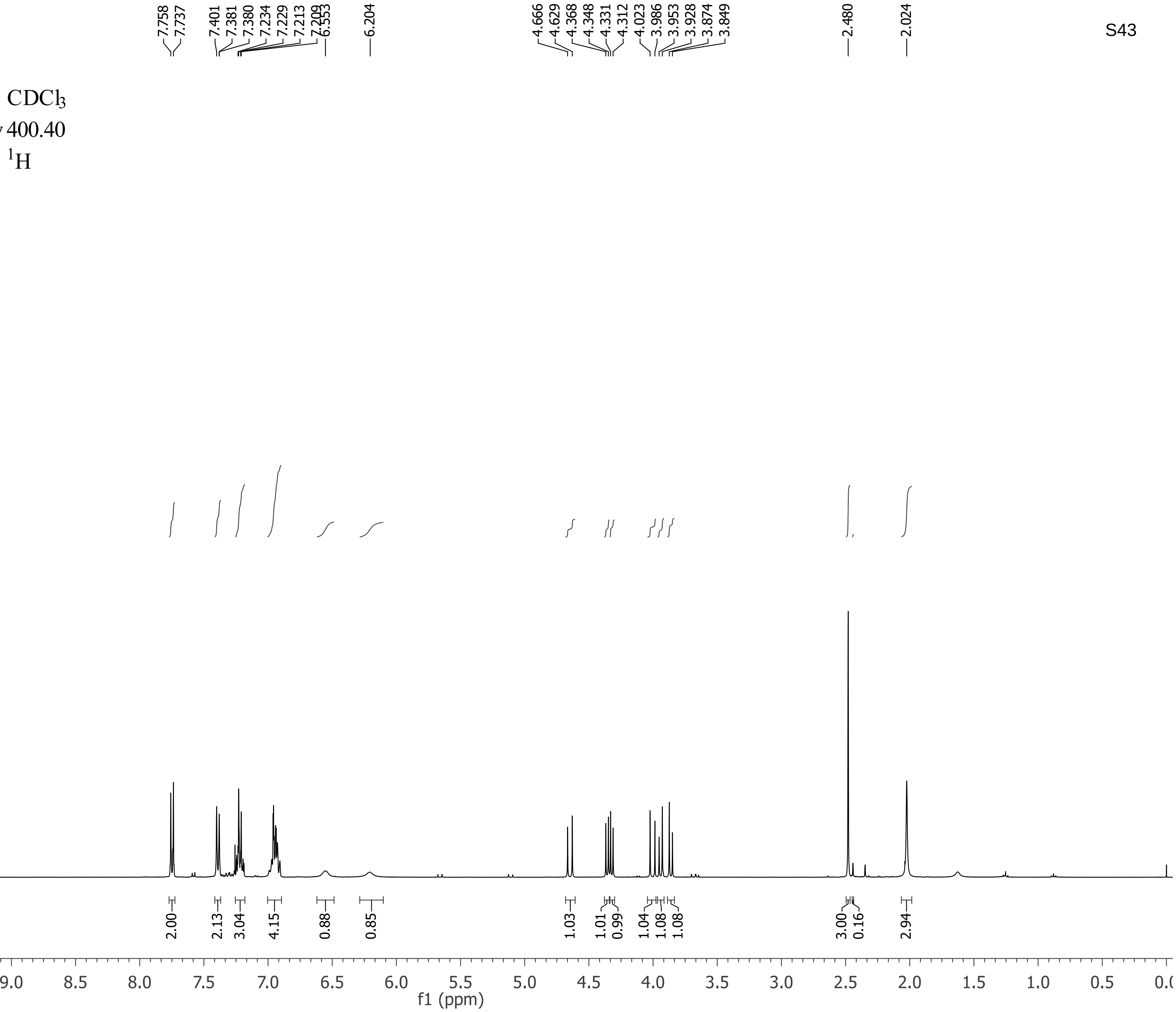

Solvent CDCl$_3$
Spectrometer Frequency 100.69
Nucleus ^{13}C{^{1}H}

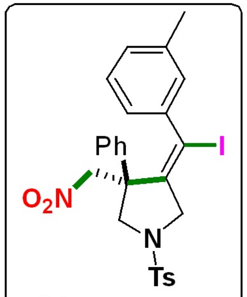

3d (*E*/*Z*= 5:95)

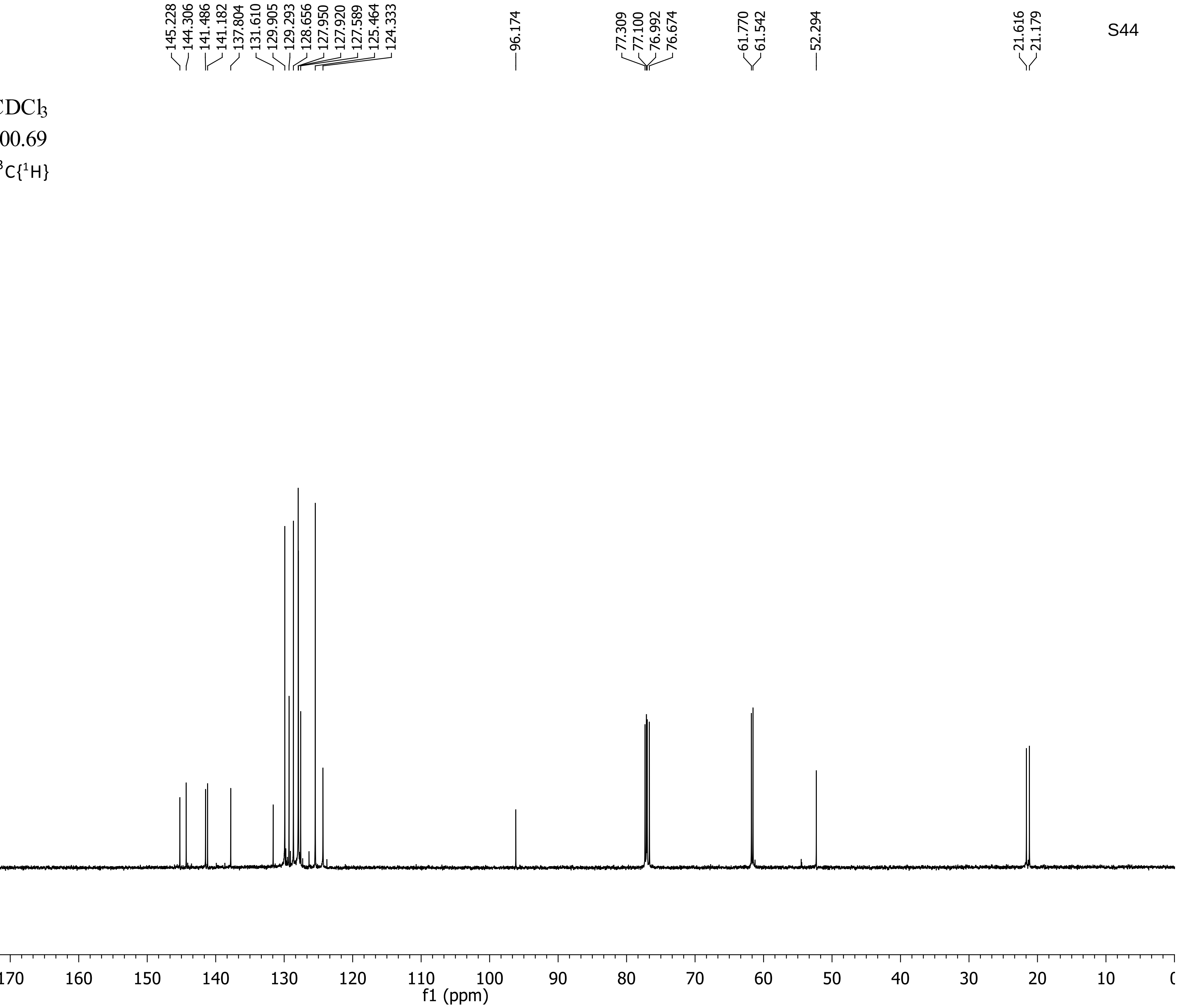

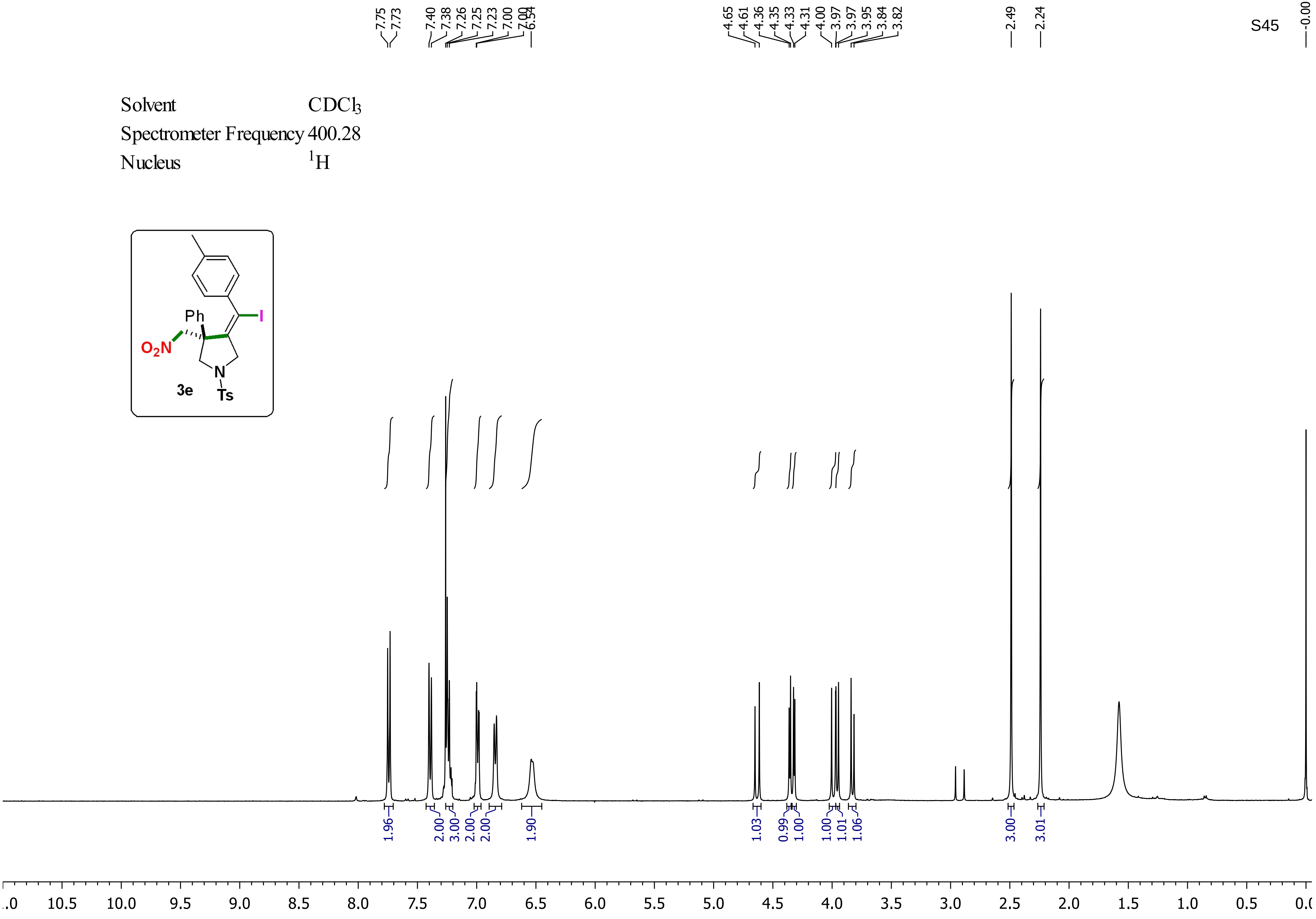
7.75
7.73
7.40
7.38
7.26
7.25
7.23
7.00
7.00
6.54
4.65
4.61
4.36
4.35
4.33
4.31
4.00
3.97
3.97
3.95
3.84
3.82
2.49
2.24
-0.00
Solvent CDCl3
Spectrometer Frequency 400.28
Nucleus 1H
Ph
O2N
I
N
Ts
3e
1.96
2.00
3.00
2.00
2.00
1.90
1.03
0.99
1.00
1.00
1.01
1.06
3.00
3.01
10.5
10.0
9.5
9.0
8.5
8.0
7.5
7.0
6.5
6.0
5.5
5.0
4.5
4.0
3.5
3.0
2.5
2.0
1.5
1.0
0.5

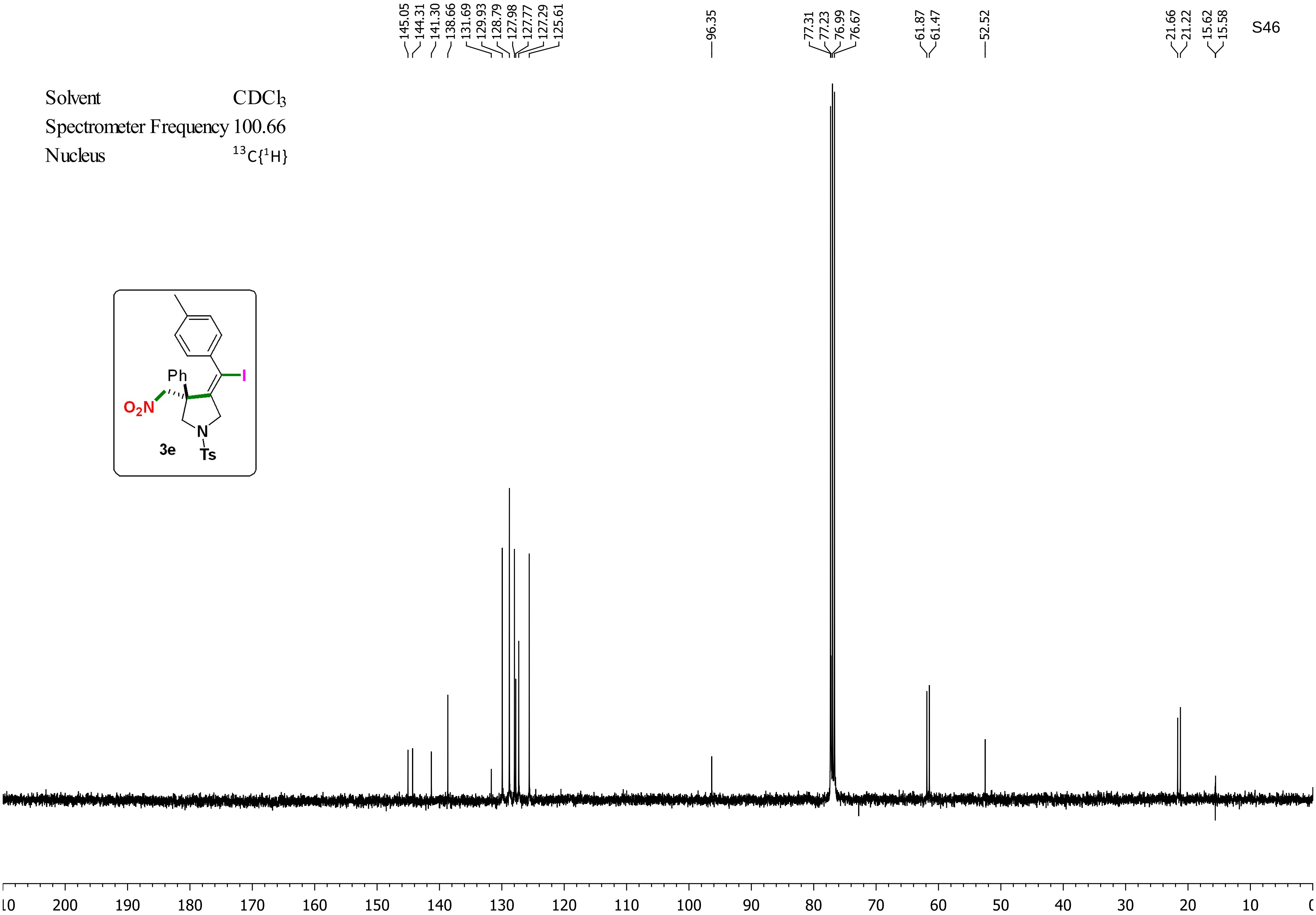
145.05
144.31
141.30
138.66
131.69
129.93
128.79
127.98
127.77
127.29
125.61
96.35
77.31
77.23
76.99
76.67
61.87
61.47
52.52
21.66
21.22
15.62
15.58
Solvent CDCl3
Spectrometer Frequency 100.66
Nucleus 13C{1H}
200
190
180
170
160
150
140
130
120
110
100
90
80
70
60
50
40
30
20
10
Ph
I
O2N
N
Ts
3e

Solvent CDCl$_3$
Spectrometer Frequency 400.28
Nucleus ^{1}H

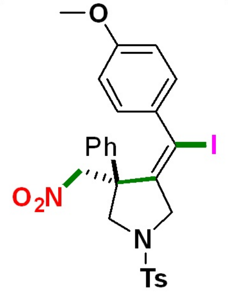

3f (*E*/Z= 15:85)

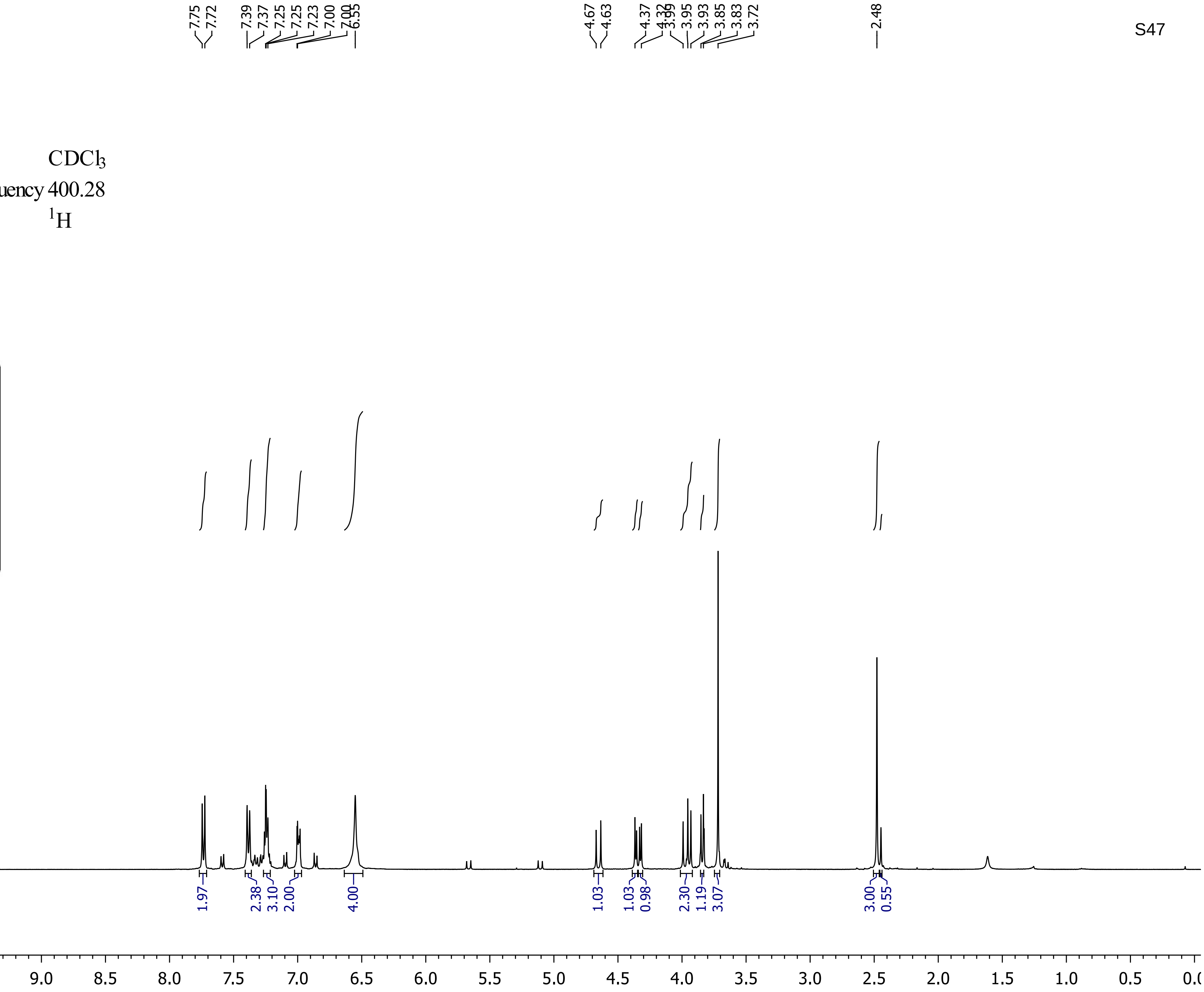

Solvent CDCl$_3$
Spectrometer Frequency 100.66
Nucleus $^{13}C\{^1H\}$

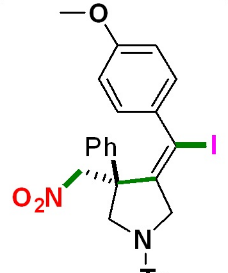

3f (*E*/Z= 15:85)

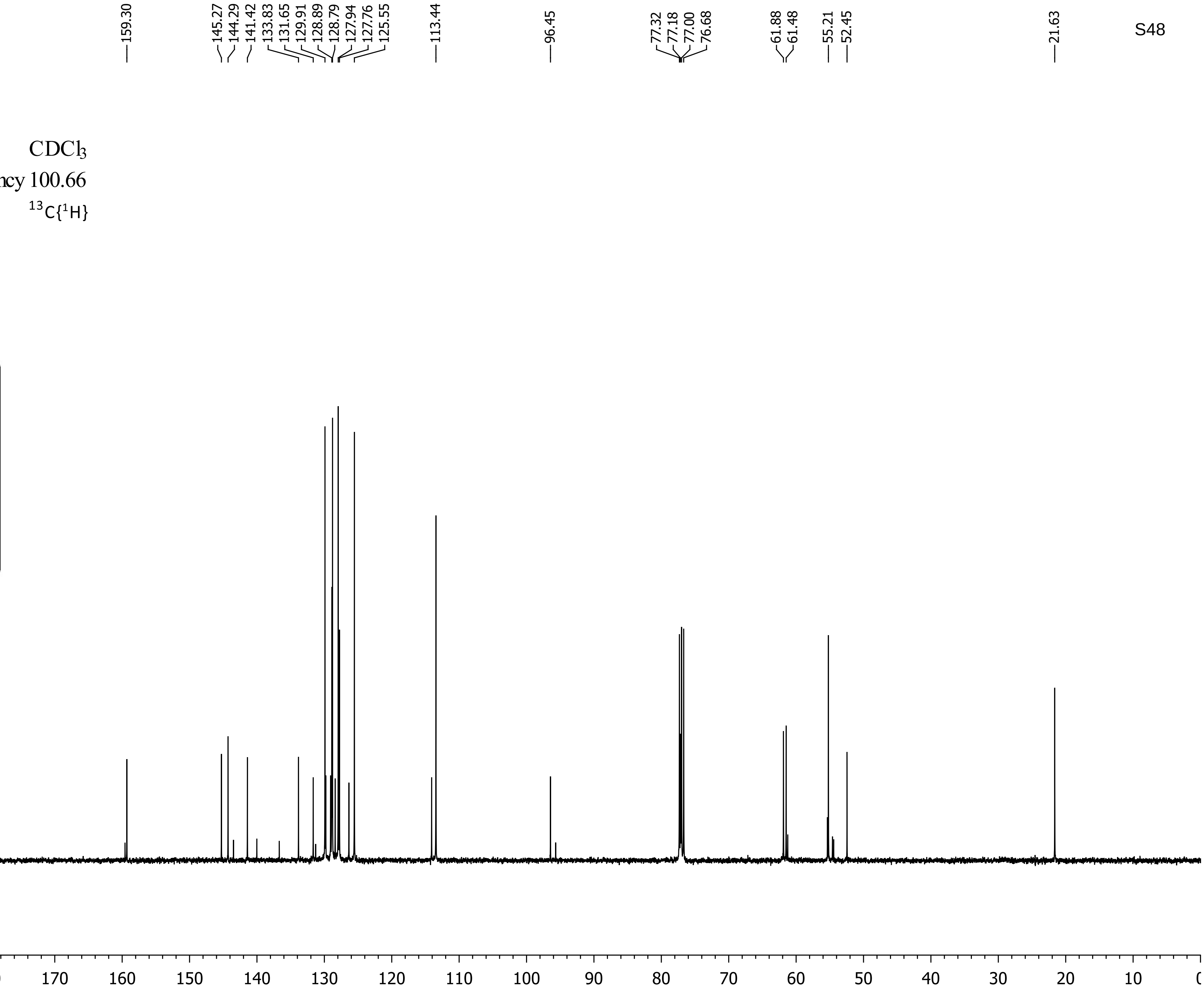

Solvent CDCl$_3$
Spectrometer Frequency 400.39
Nucleus ^{1}H

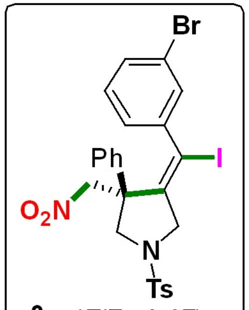

3g (*E*/Z= 3:97)

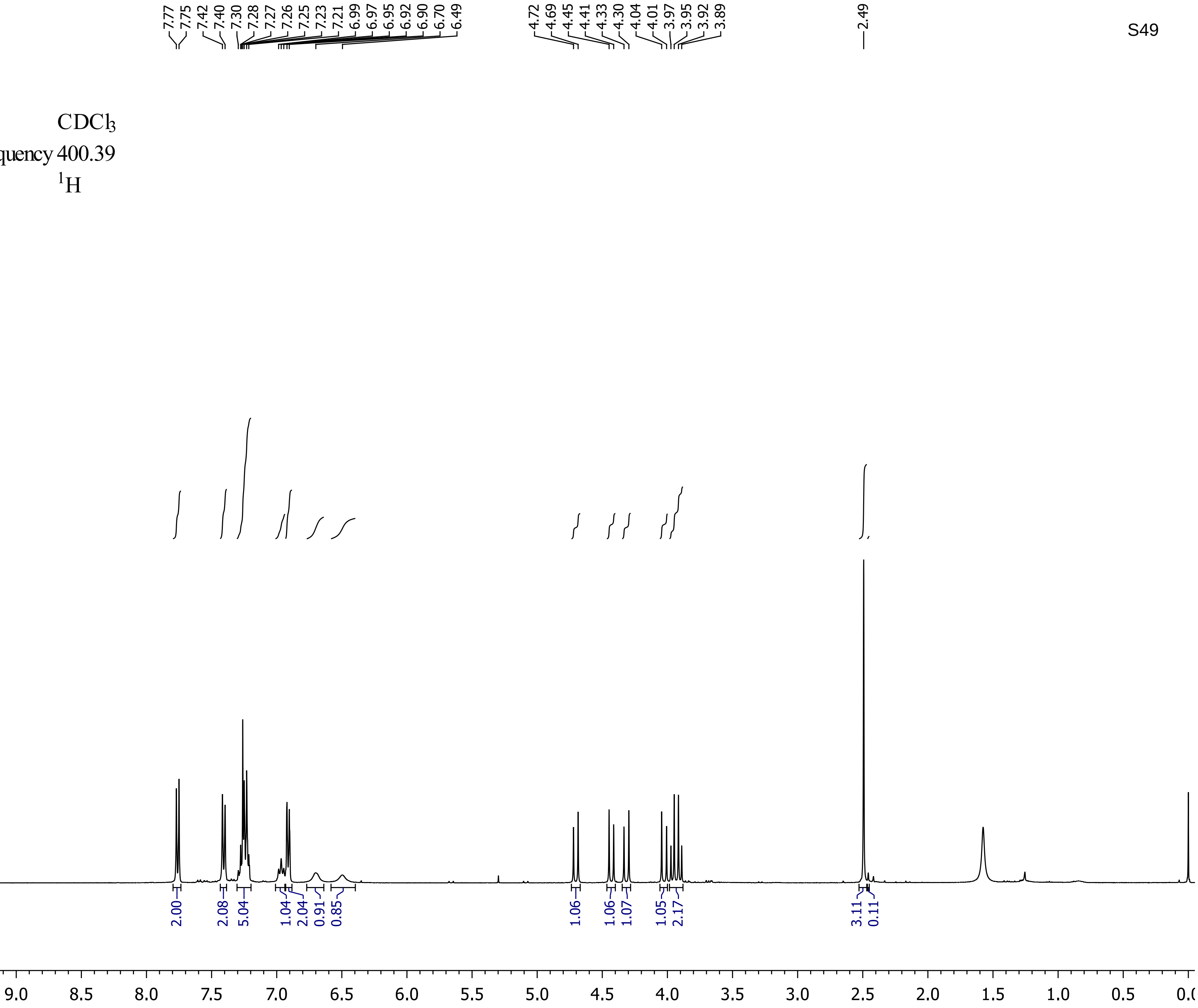

Solvent CDCl$_3$
Spectrometer Frequency 100.69
Nucleus $^{13}C\{^1H\}$

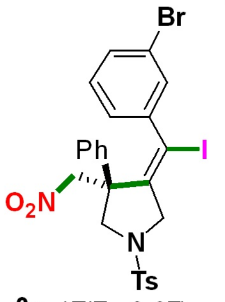

3g (*E*/Z= 3:97)

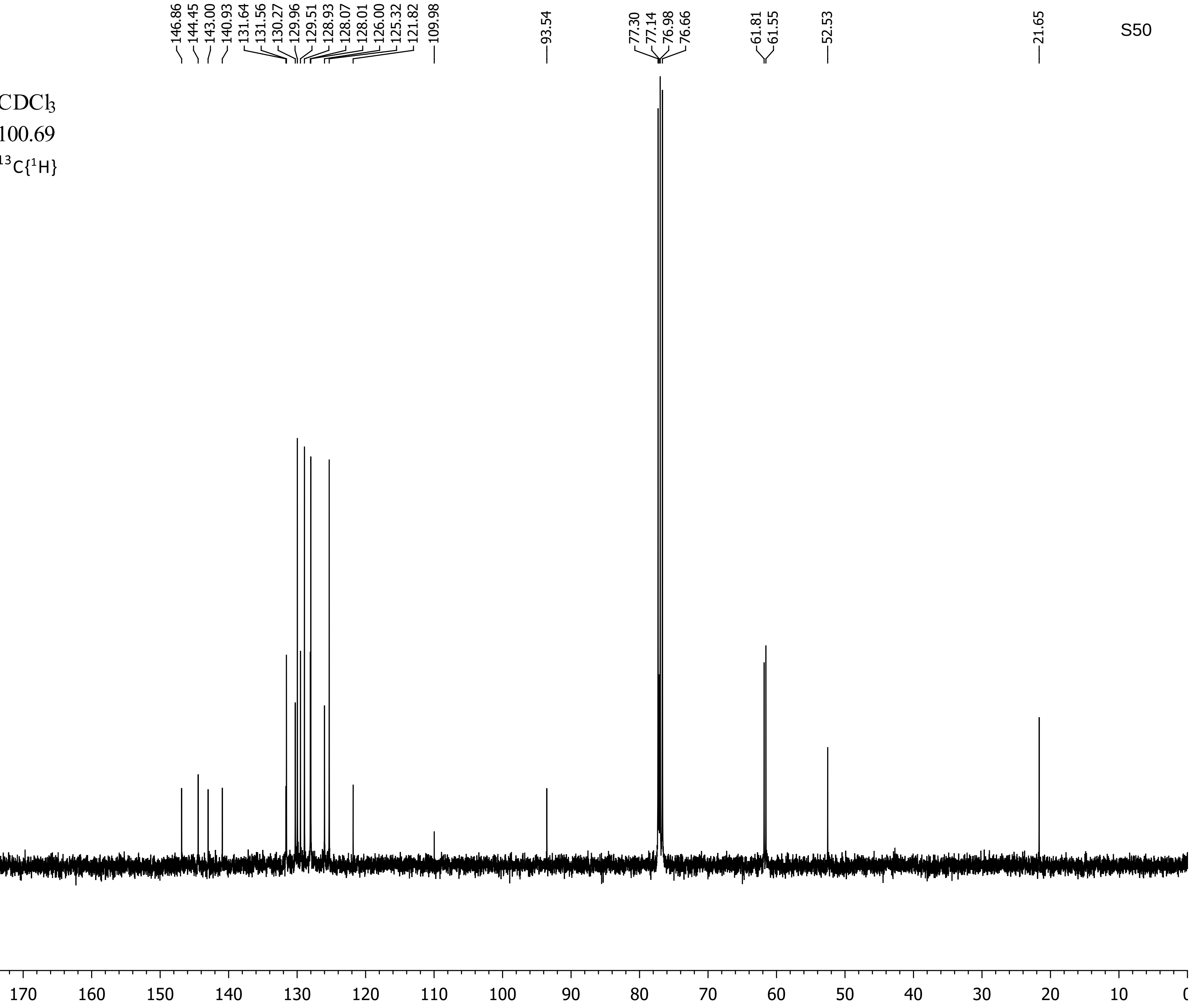

Solvent	$CDCl_3$
Spectrometer Frequency	400.28
Nucleus	^1H

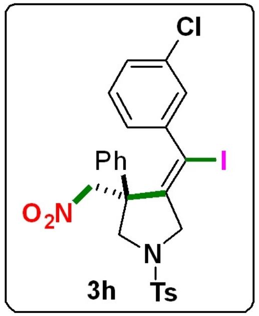

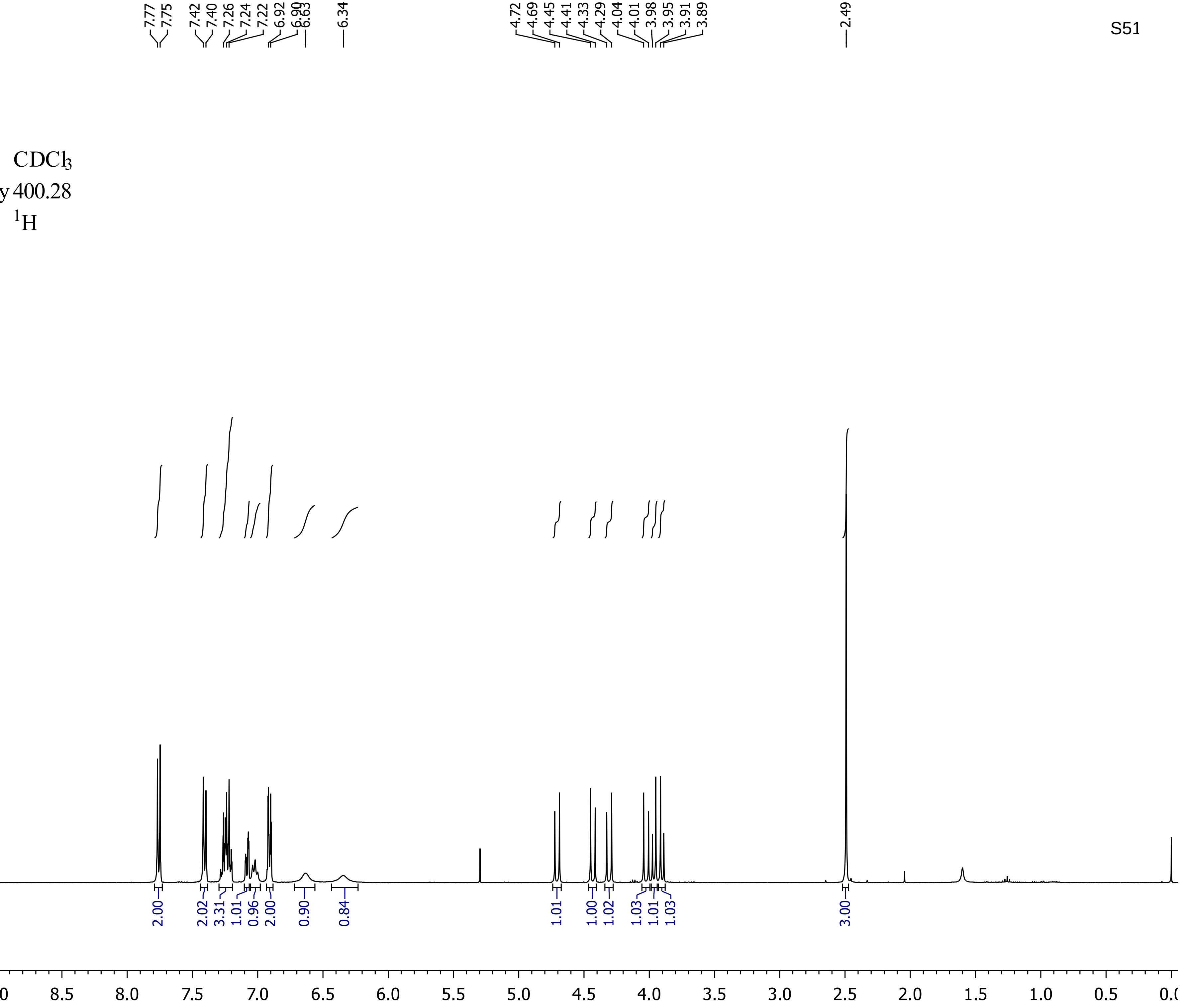

Solvent CDCl3
Spectrometer Frequency 100.66
Nucleus $^{13}C\{^{1}H\}$

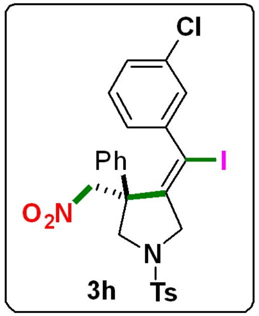

3h

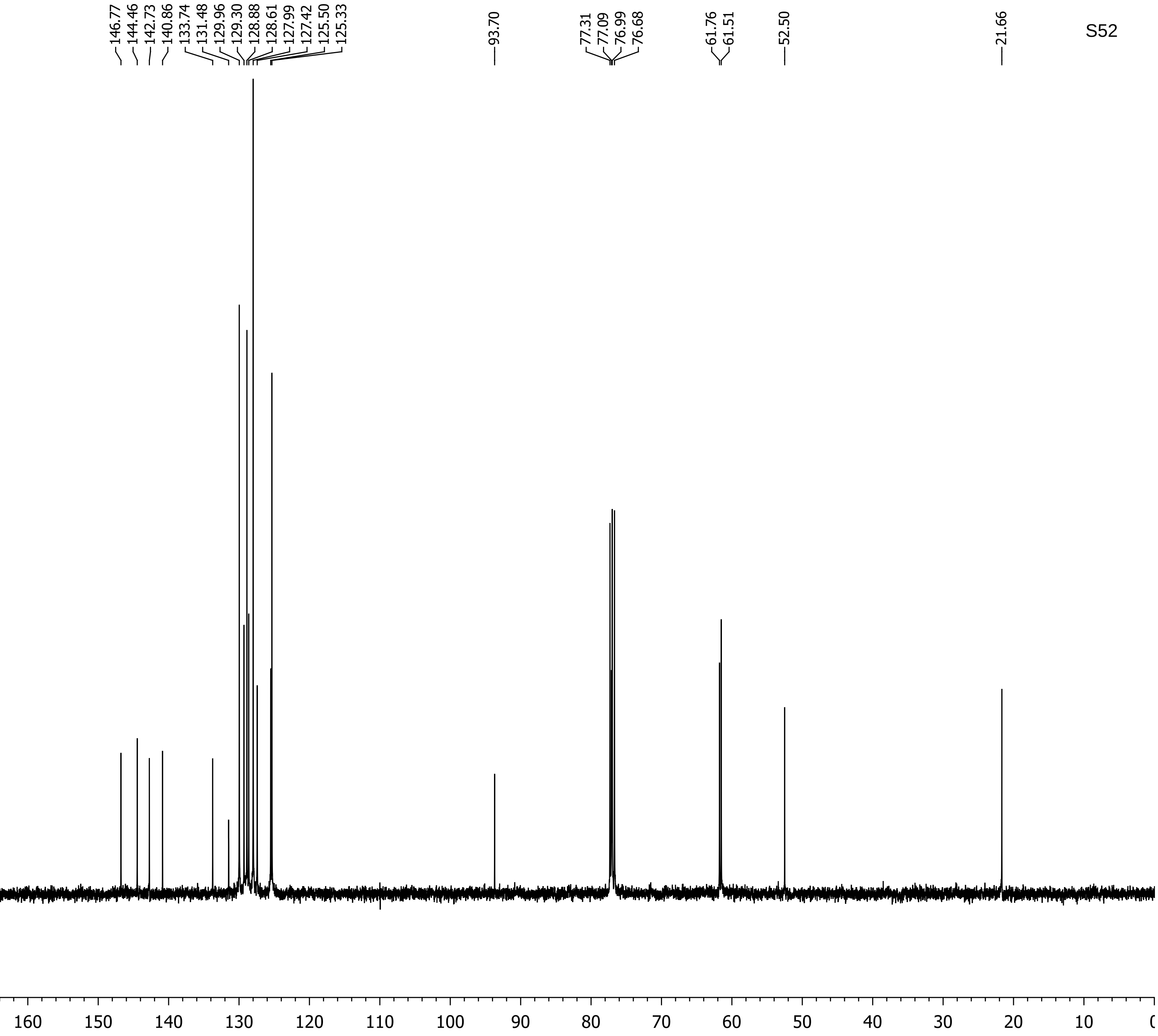

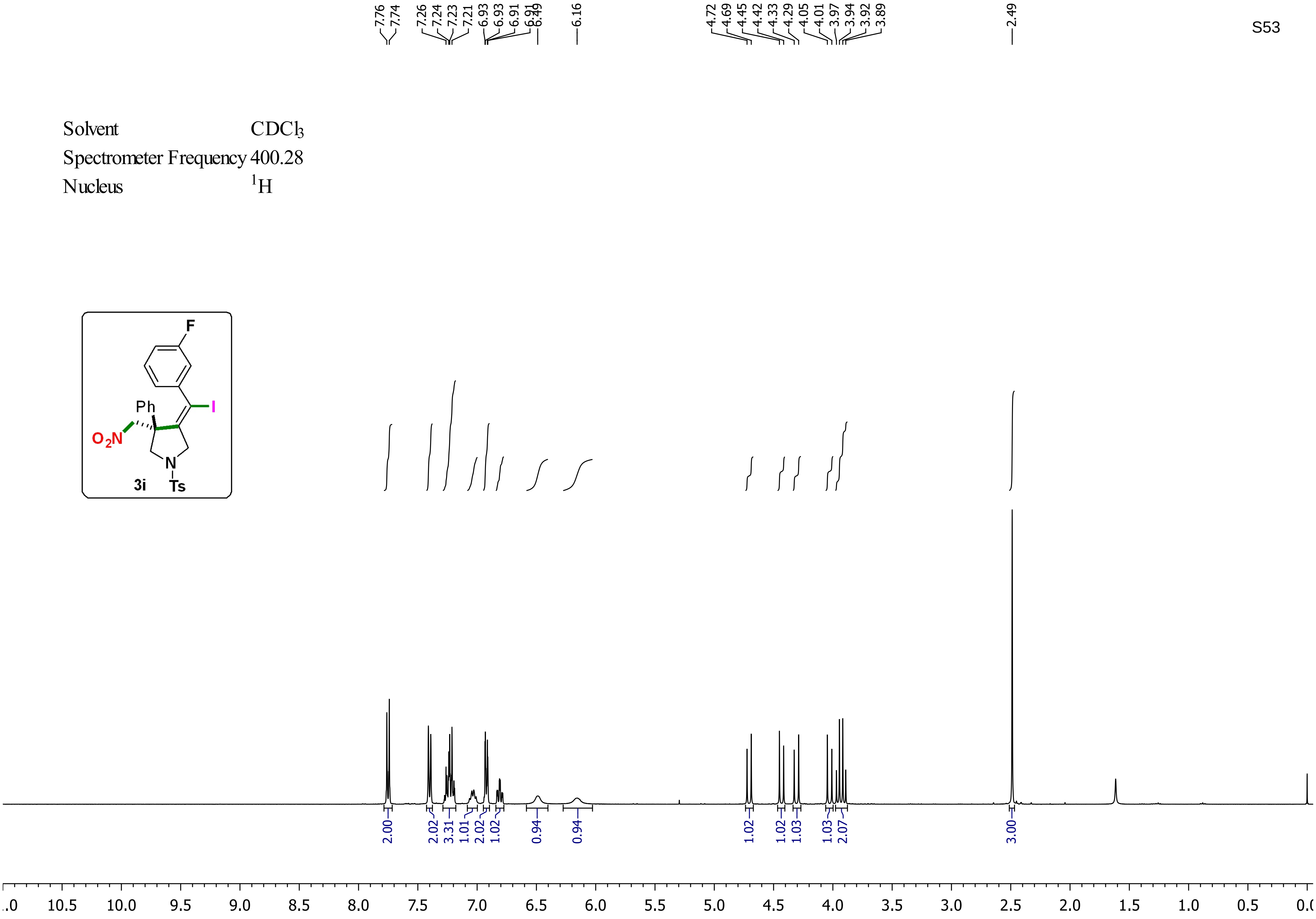

Solvent CDCl3
Spectrometer Frequency 400.28
Nucleus 1H
7.76 7.74 7.26 7.24 7.23 7.21 6.93 6.93 6.91 6.91 6.49 6.16
4.72 4.69 4.45 4.42 4.33 4.29 4.05 4.01 3.97 3.94 3.92 3.89
2.49
2.00 2.02 3.31 1.01 2.02 1.02 0.94 0.94 1.02 1.02 1.03 1.03 2.07 3.00
10.5 10.0 9.5 9.0 8.5 8.0 7.5 7.0 6.5 6.0 5.5 5.0 4.5 4.0 3.5 3.0 2.5 2.0 1.5 1.0 0.5
F
Ph
I
O2N
N
Ts
3i

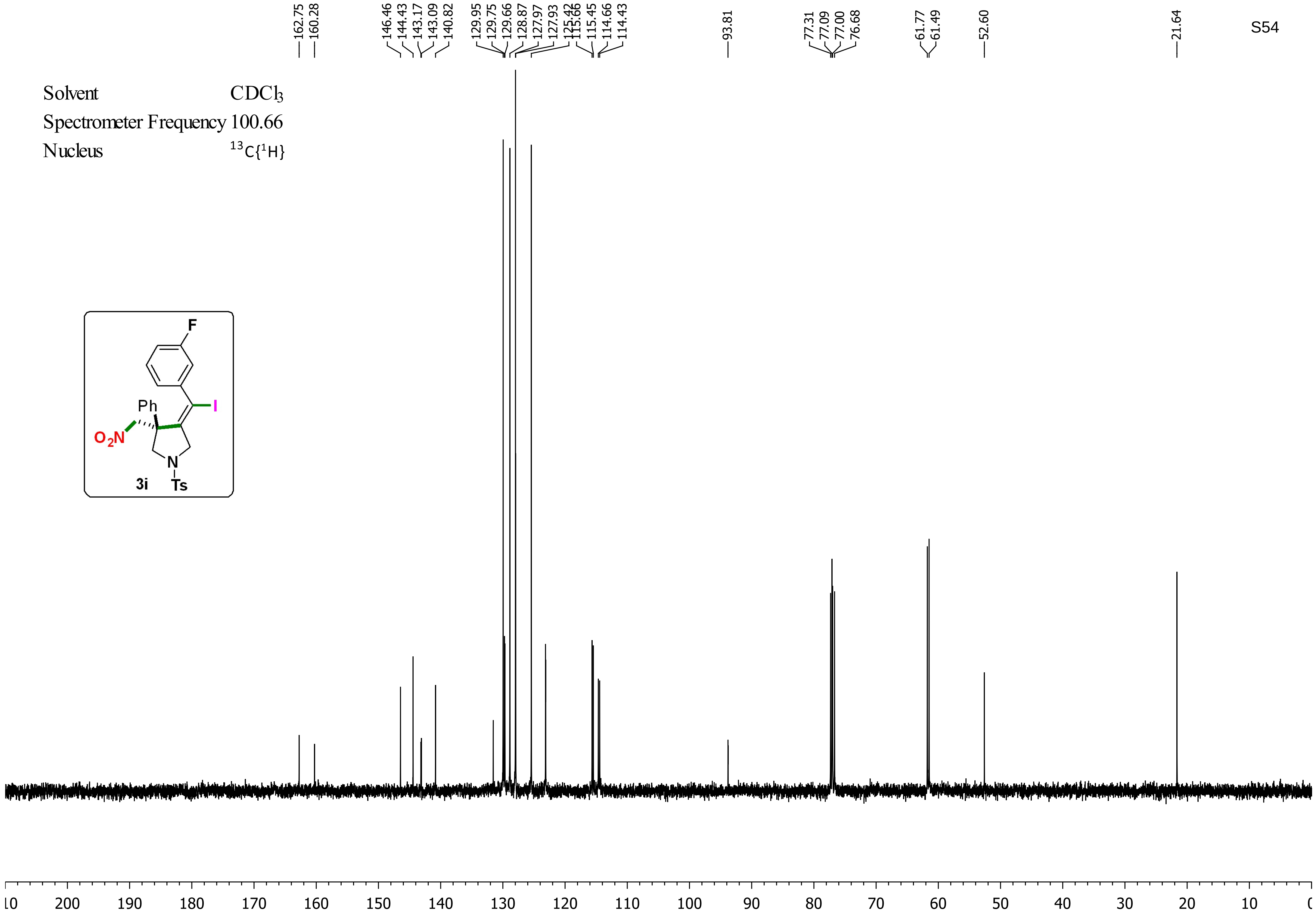
162.75
160.28
146.46
144.43
143.17
143.09
140.82
129.95
129.75
129.66
128.87
127.97
127.93
125.42
115.66
115.45
114.66
114.43
93.81
77.31
77.09
77.00
76.68
61.77
61.49
52.60
21.64
Solvent CDCl3
Spectrometer Frequency 100.66
Nucleus 13C{1H}
F
Ph
I
O2N
N
Ts
3i
200 190 180 170 160 150 140 130 120 110 100 90 80 70 60 50 40 30 20 10

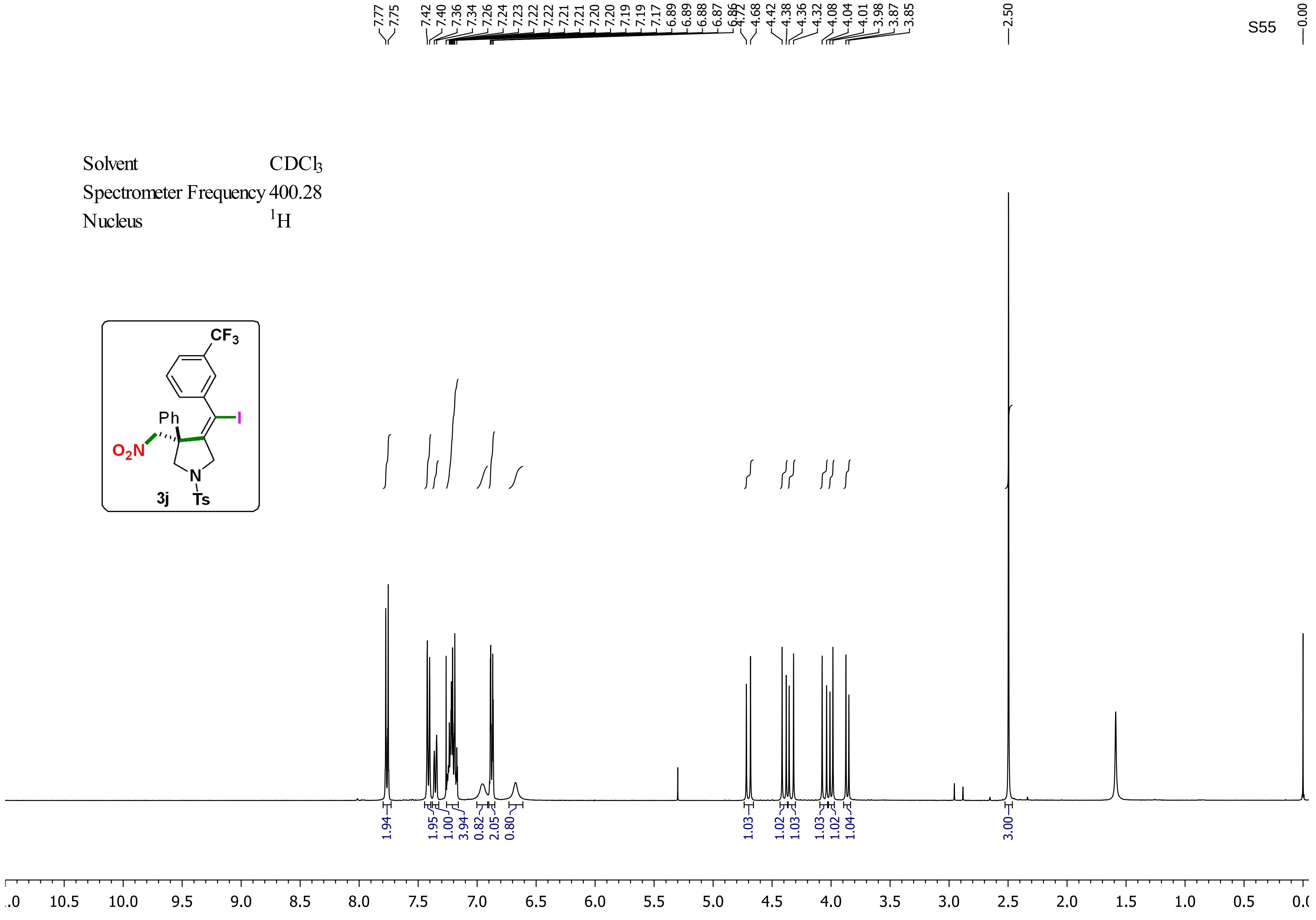
7.77
7.75
7.42
7.40
7.36
7.34
7.26
7.24
7.23
7.22
7.22
7.21
7.21
7.20
7.20
7.19
7.19
7.17
6.89
6.89
6.88
6.87
6.86
4.72
4.68
4.42
4.38
4.36
4.32
4.08
4.04
4.01
3.98
3.87
3.85
2.50
0.00
Solvent CDCl3
Spectrometer Frequency 400.28
Nucleus 1H
CF3
Ph
I
O2N
N
Ts
3j
1.94
1.95
1.00
3.94
0.82
2.05
0.80
1.03
1.02
1.03
1.03
1.02
1.04
3.00
10.5
10.0
9.5
9.0
8.5
8.0
7.5
7.0
6.5
6.0
5.5
5.0
4.5
4.0
3.5
3.0
2.5
2.0
1.5
1.0
0.5

Solvent CDCl3
Spectrometer Frequency 100.66
Nucleus $^{13}C\{^1H\}$

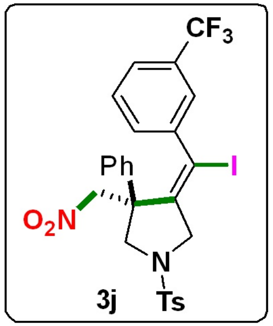

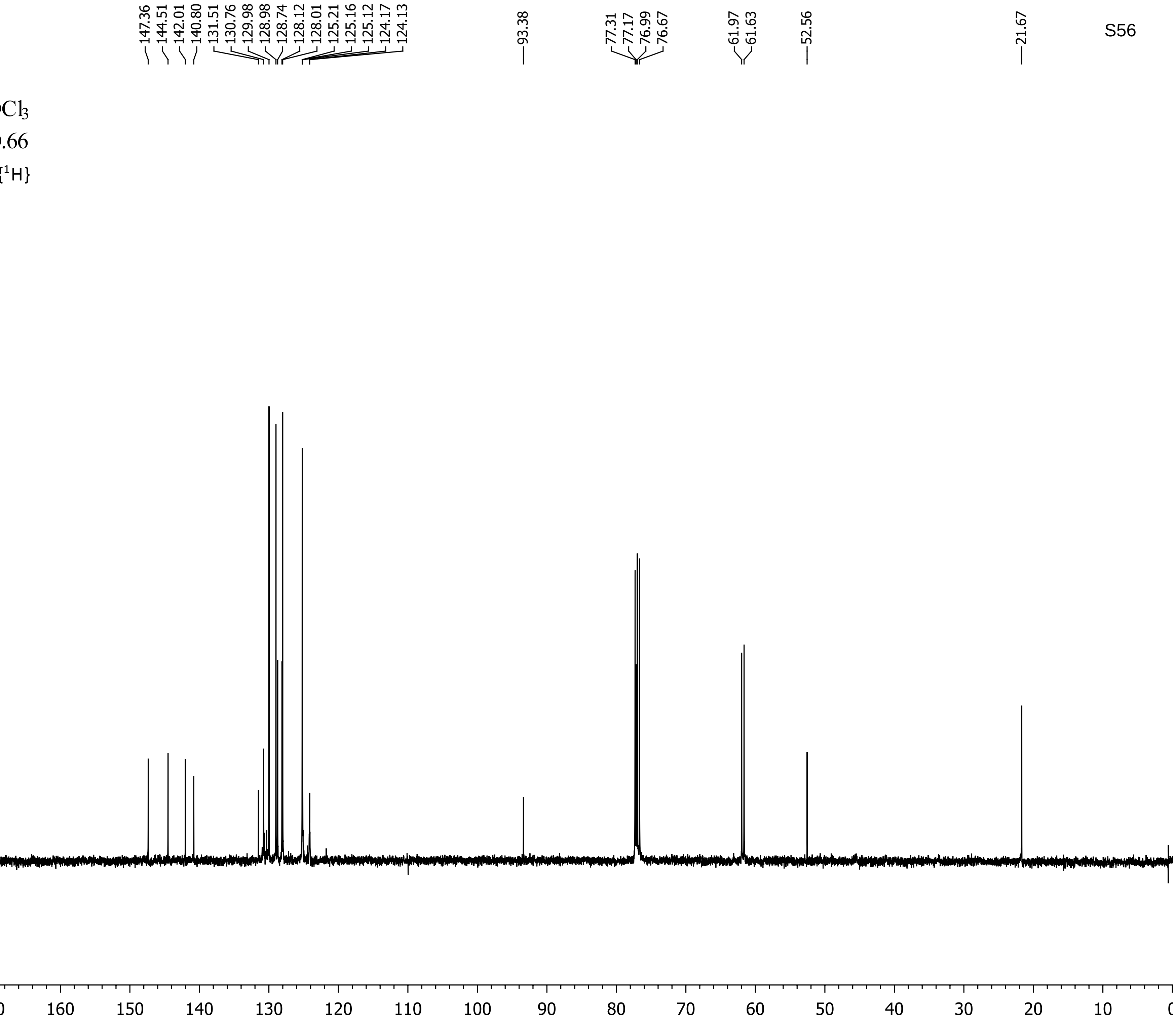

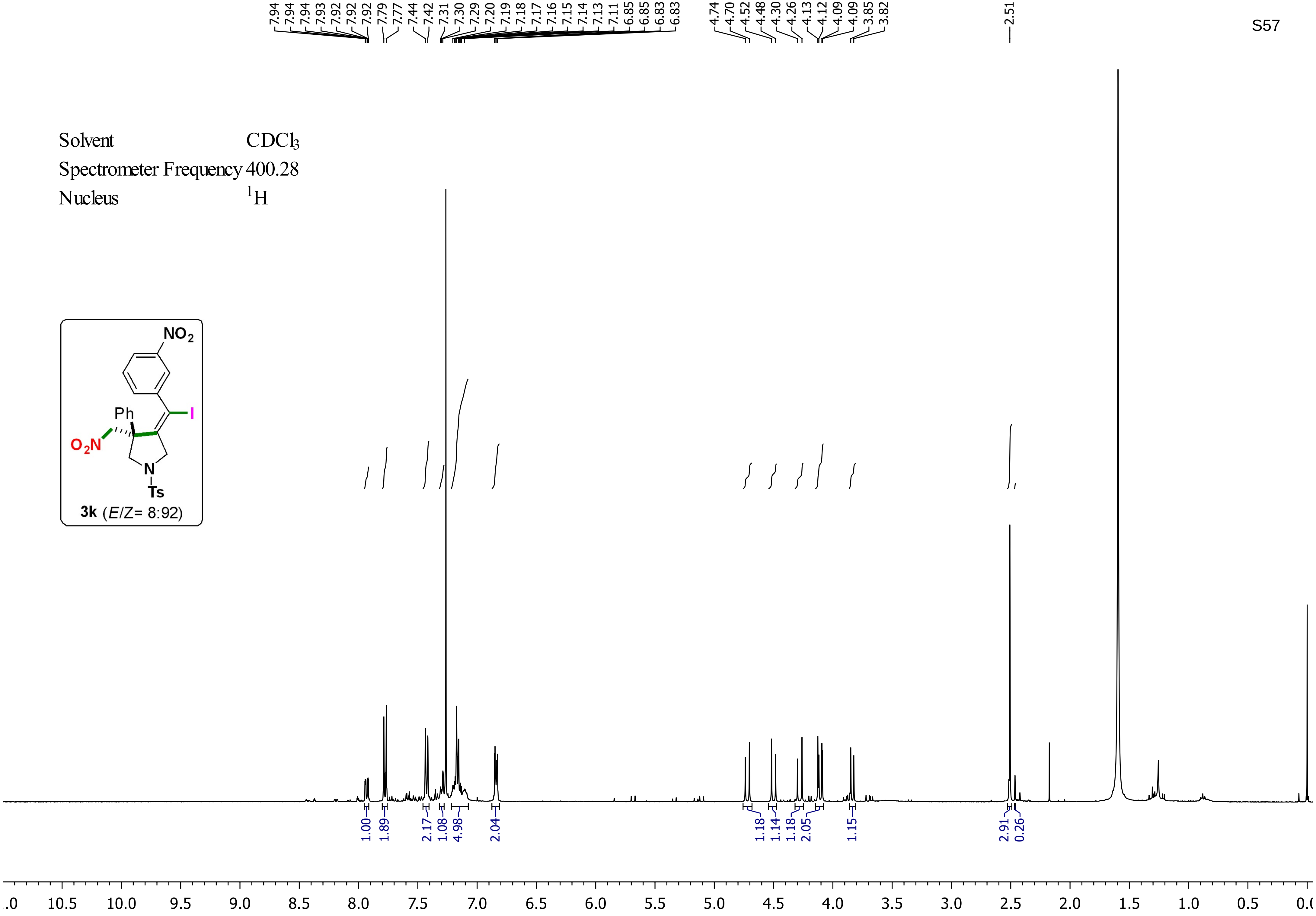
7.94 7.94 7.94 7.93 7.92 7.92 7.92 7.79 7.77 7.44 7.42 7.31 7.30 7.29 7.20 7.19 7.18 7.17 7.16 7.15 7.14 7.13 7.11 6.85 6.85 6.83 6.83
4.74 4.70 4.52 4.48 4.30 4.26 4.13 4.12 4.09 4.09 3.85 3.82
2.51
Solvent CDCl$_3$
Spectrometer Frequency 400.28
Nucleus ^{1}H
NO$_2$
Ph
I
O$_2$N
N
Ts
3k (E/Z= 8:92)
1.00 1.89 2.17 1.08 4.98 2.04
1.18 1.14 1.18 2.05 1.15
2.91 0.26
10.5 10.0 9.5 9.0 8.5 8.0 7.5 7.0 6.5 6.0 5.5 5.0 4.5 4.0 3.5 3.0 2.5 2.0 1.5 1.0 0.5

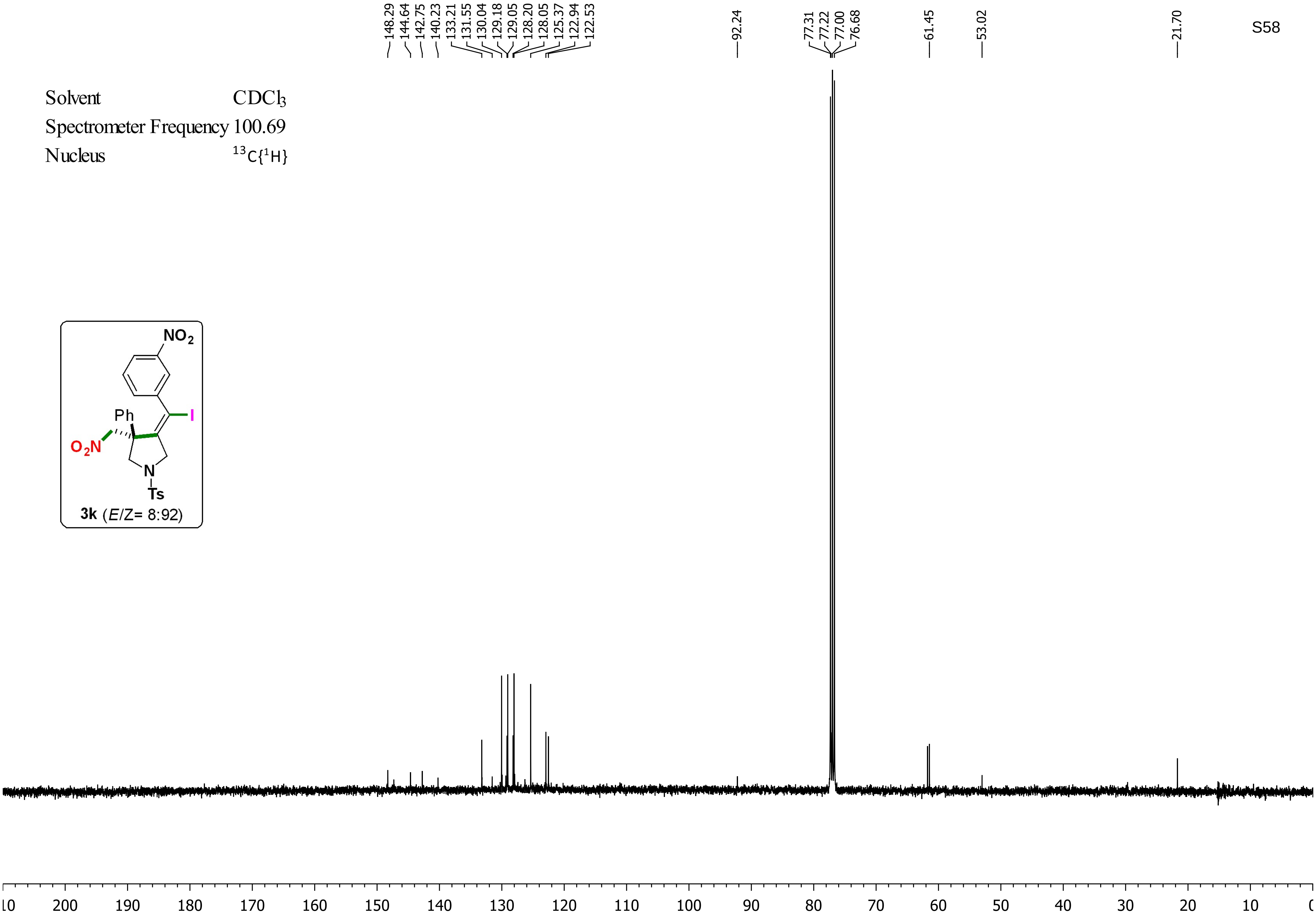
Solvent CDCl3
Spectrometer Frequency 100.69
Nucleus 13C{1H}
148.29
144.64
142.75
140.23
133.21
131.55
130.04
129.18
129.05
128.20
128.05
125.37
122.94
122.53
92.24
77.31
77.22
77.00
76.68
61.45
53.02
21.70
NO2
Ph
I
O2N
N
Ts
3k (E/Z= 8:92)
200
190
180
170
160
150
140
130
120
110
100
90
80
70
60
50
40
30
20
10

Solvent CDCl$_3$
Spectrometer Frequency 400.39
Nucleus ^{1}H

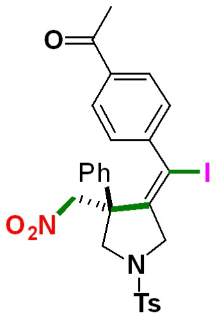

3l (*E*/*Z*= 5:95)

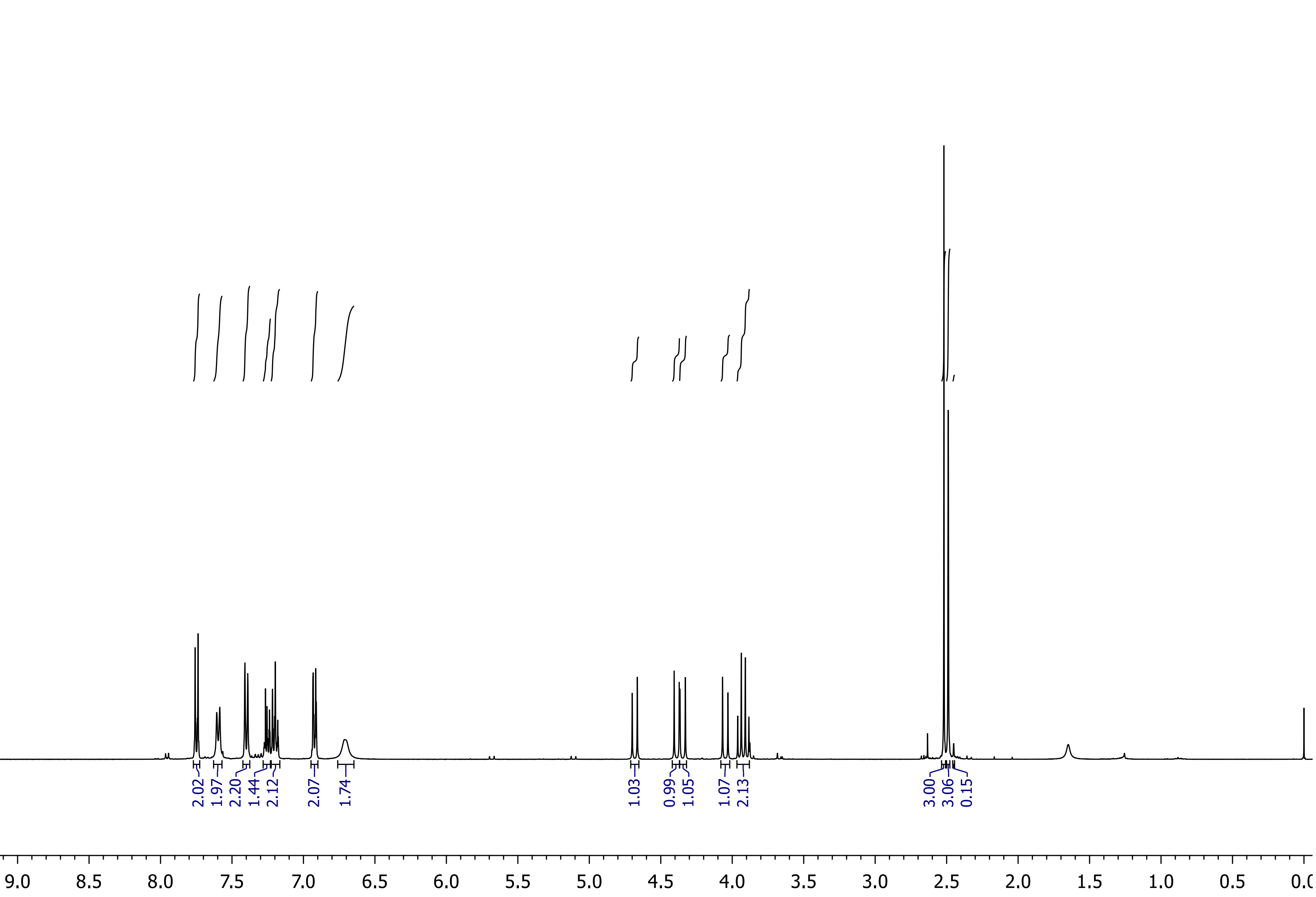

Solvent	$CDCl_3$
Spectrometer Frequency	100.69
Nucleus	$^{13}C\{^1H\}$

3l (*E*/*Z*= 5:95)

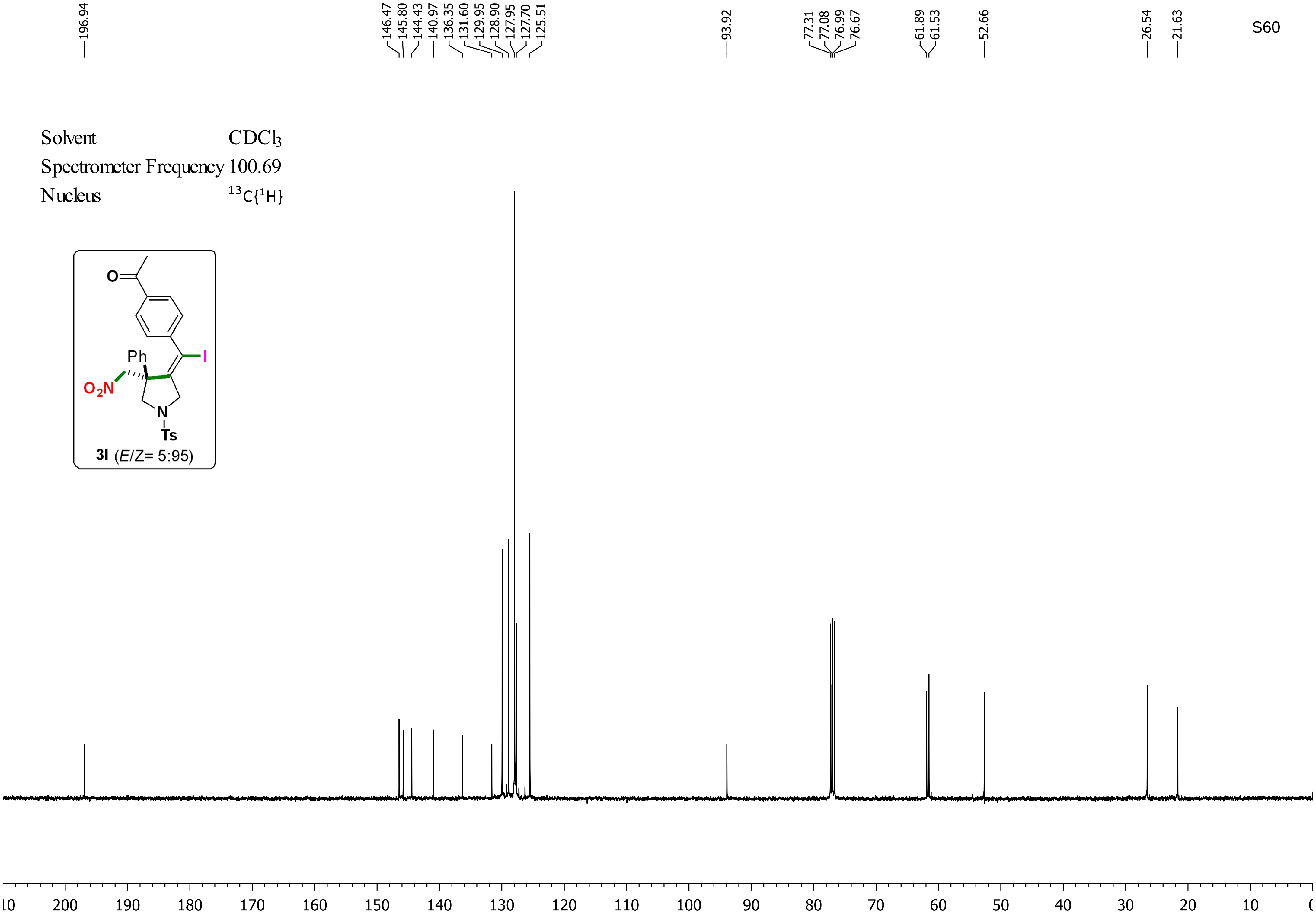

Solvent	$CDCl_3$
Spectrometer Frequency	400.28
Nucleus	^1H

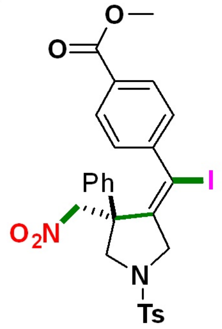

3m (*E*/Z= 5:95)

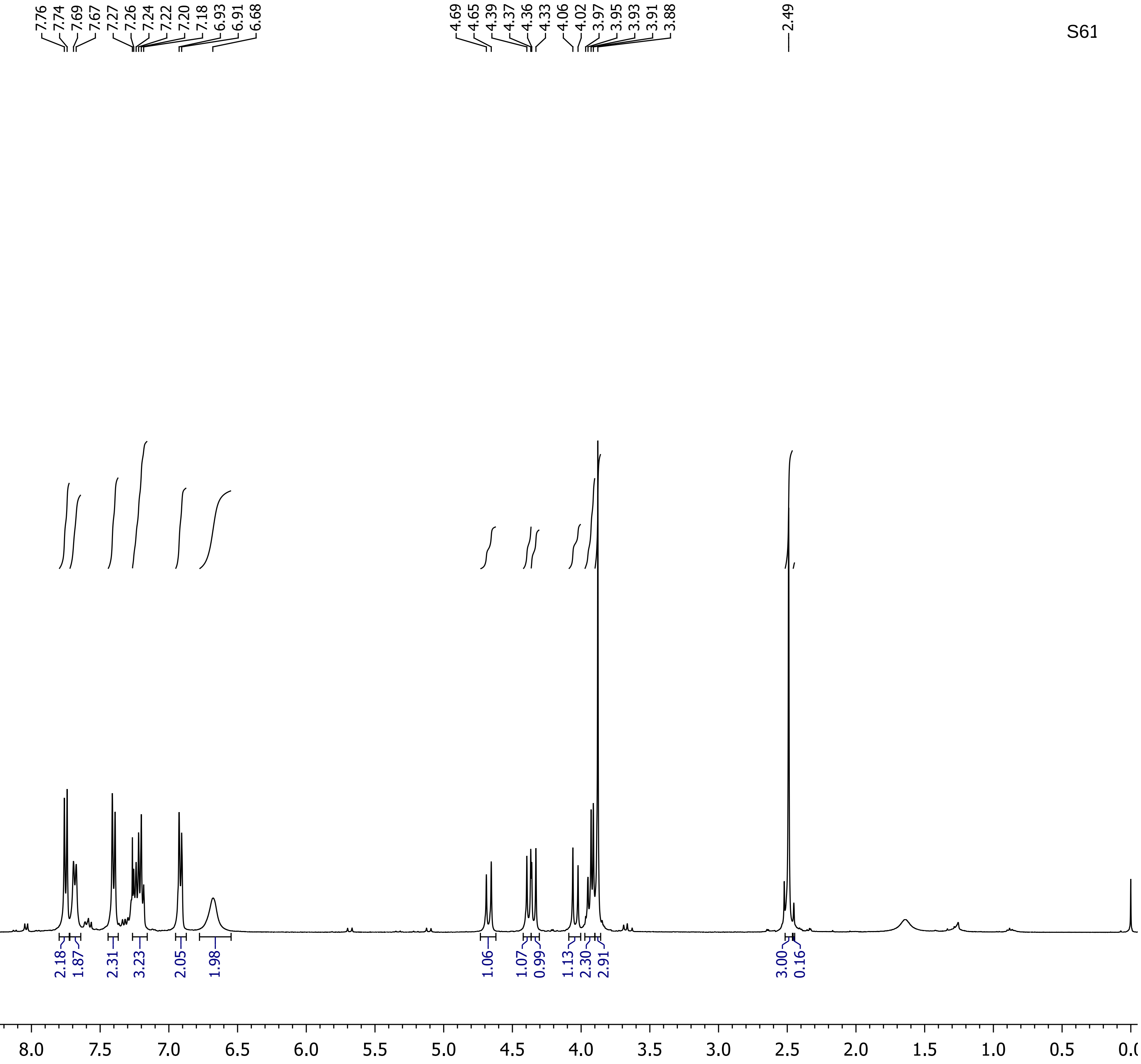

Solvent CDCl$_3$
Spectrometer Frequency 100.66
Nucleus $^{13}C\{^1H\}$

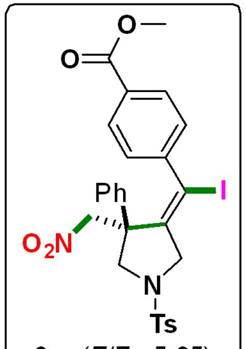

3m (*E*/Z= 5:95)

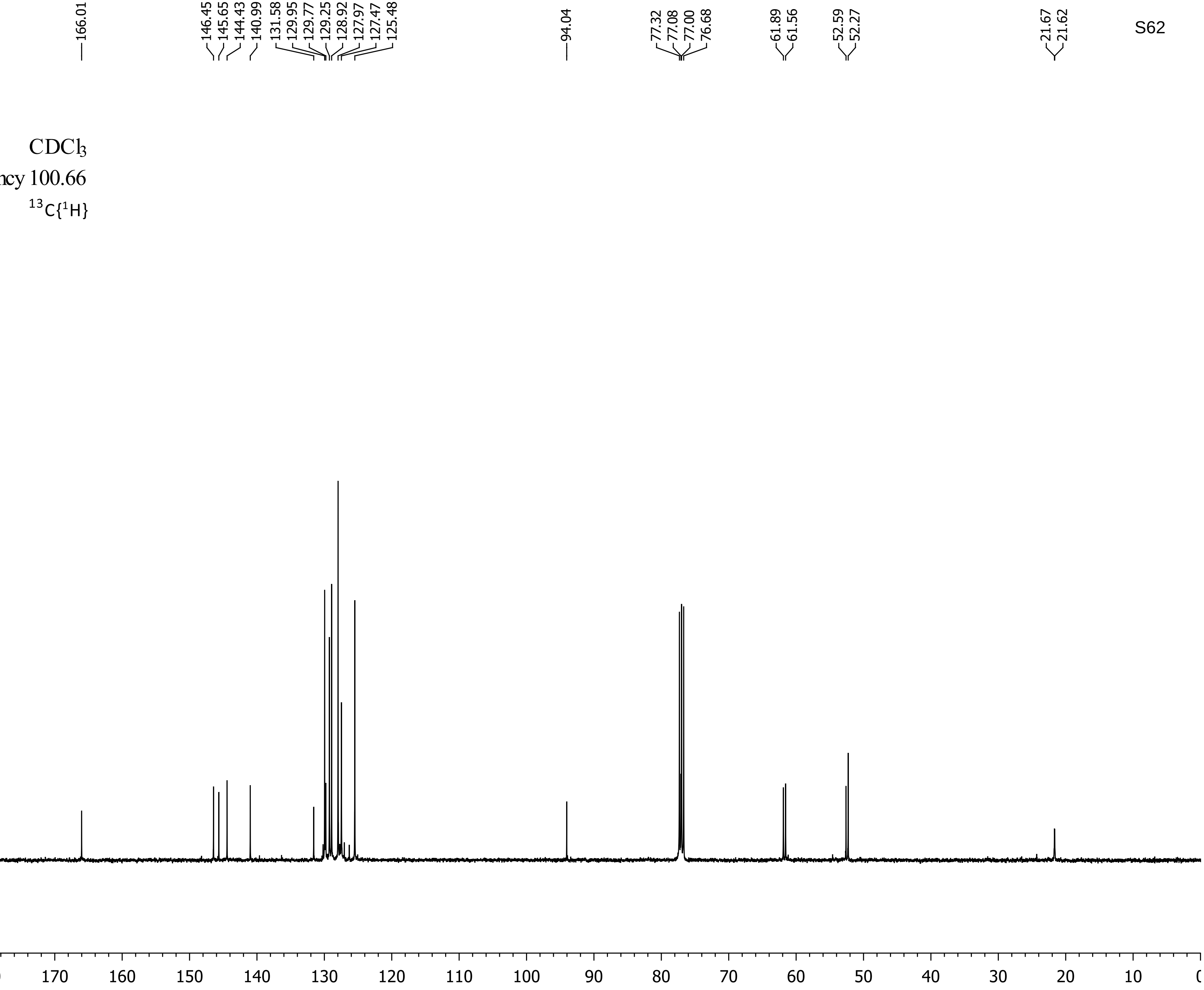

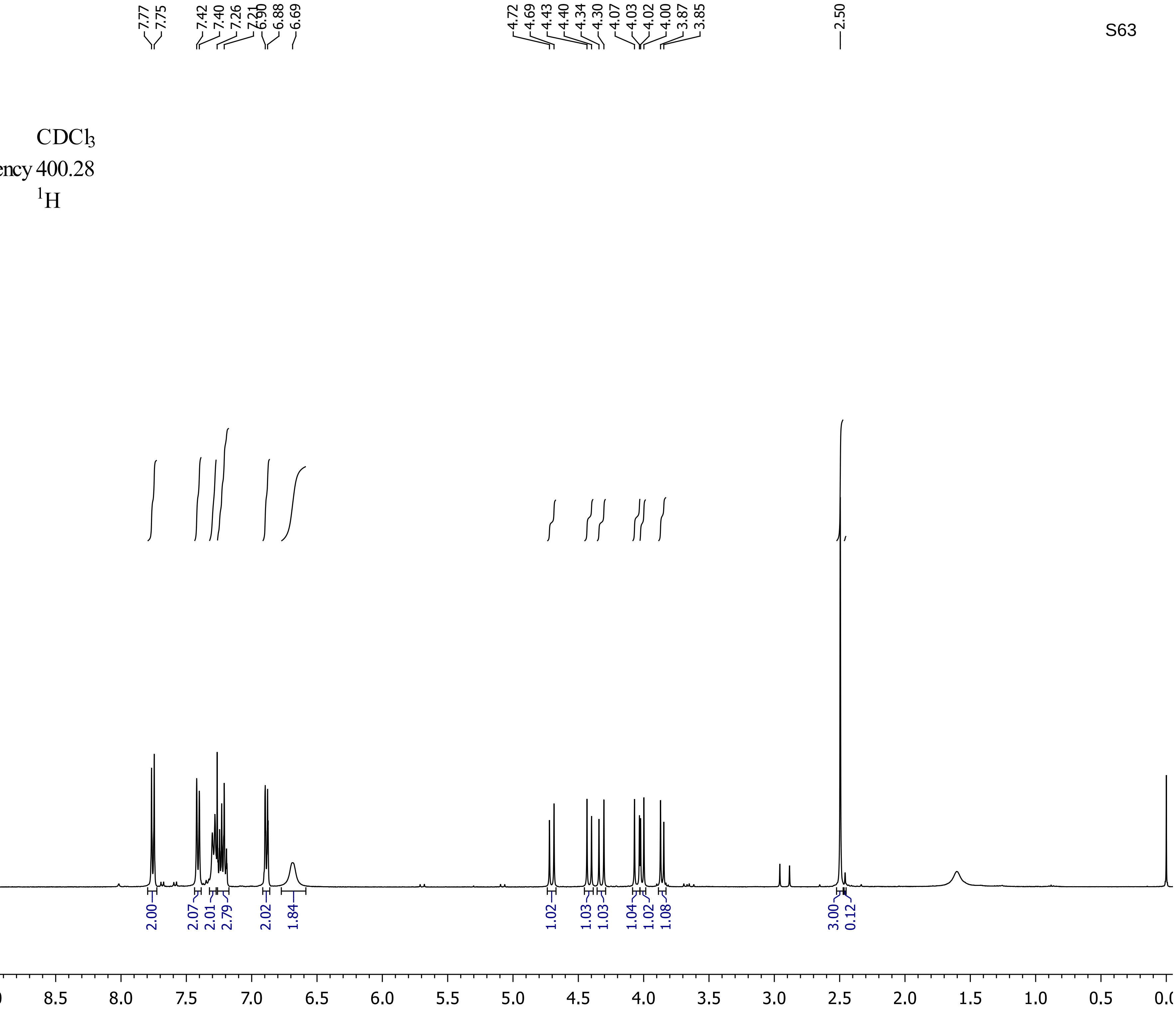

Solvent	$CDCl_3$
Spectrometer Frequency	400.28
Nucleus	^1H

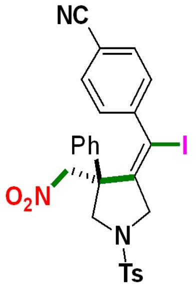

3n (*E*/*Z*= 6:94)

Solvent CDCl$_3$
Spectrometer Frequency 100.66
Nucleus $^{13}C\{^{1}H\}$

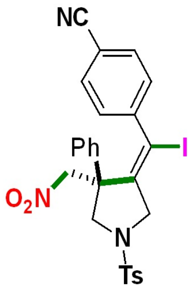

3n (*E*/Z= 6:94)

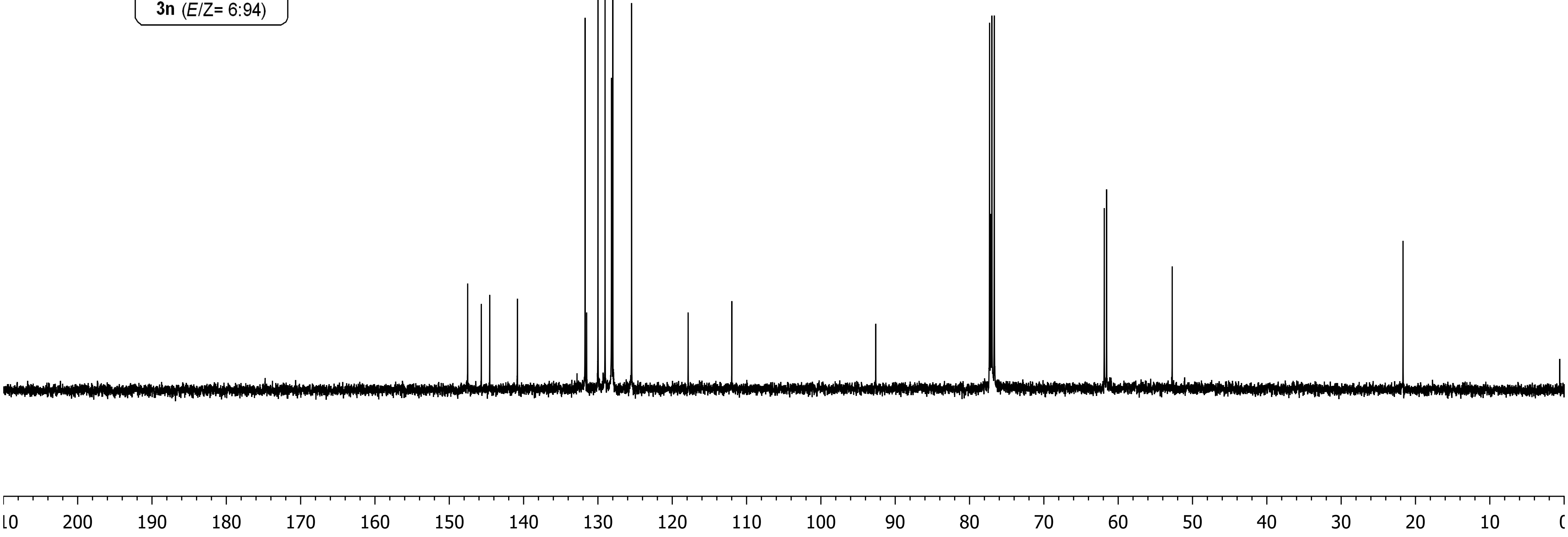

Solvent $CDCl_3$
Spectrometer Frequency 597.21
Nucleus ^1H

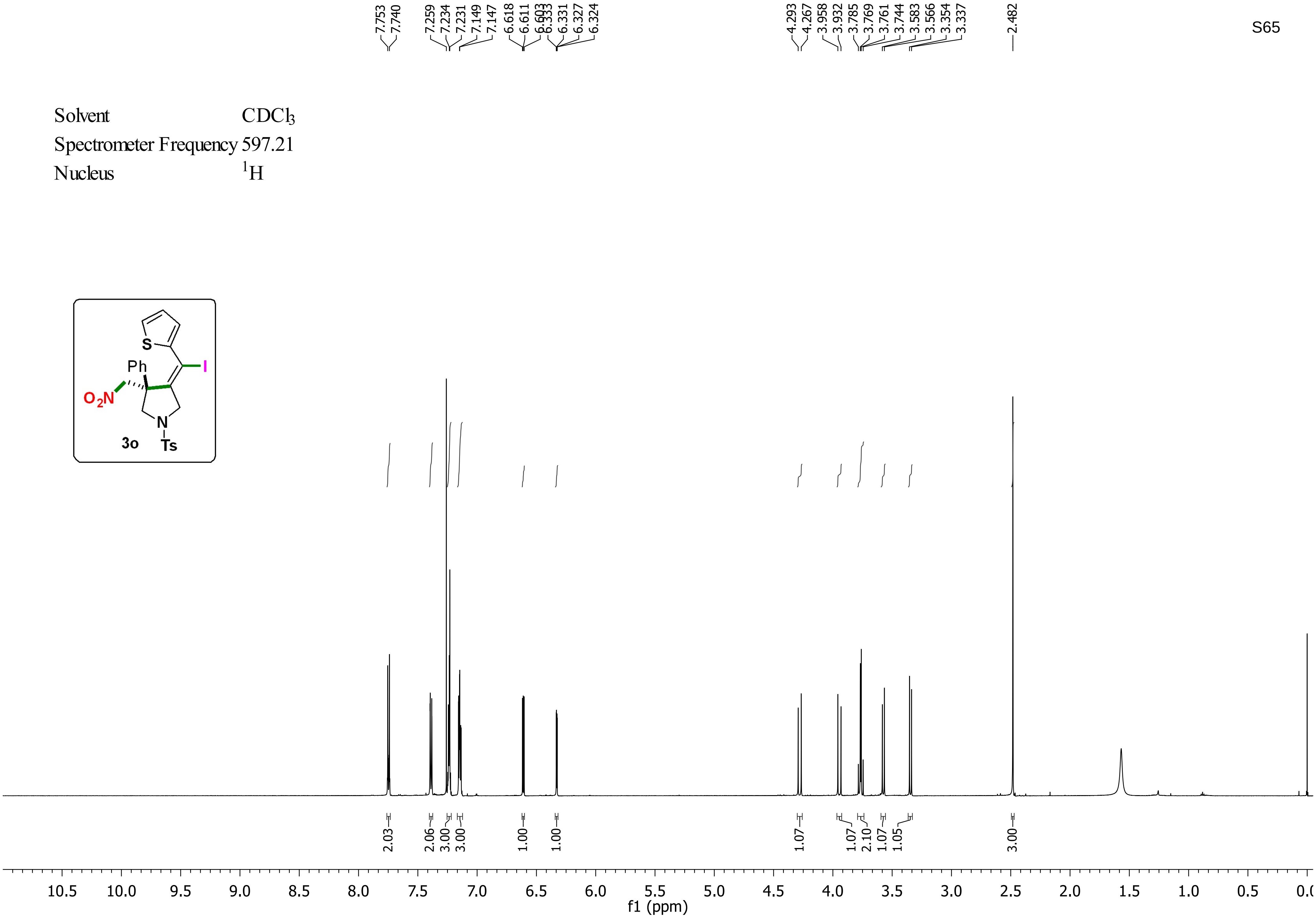

Solvent CDCl$_3$
Spectrometer Frequency 150.18
Nucleus $^{13}C\{^{1}H\}$

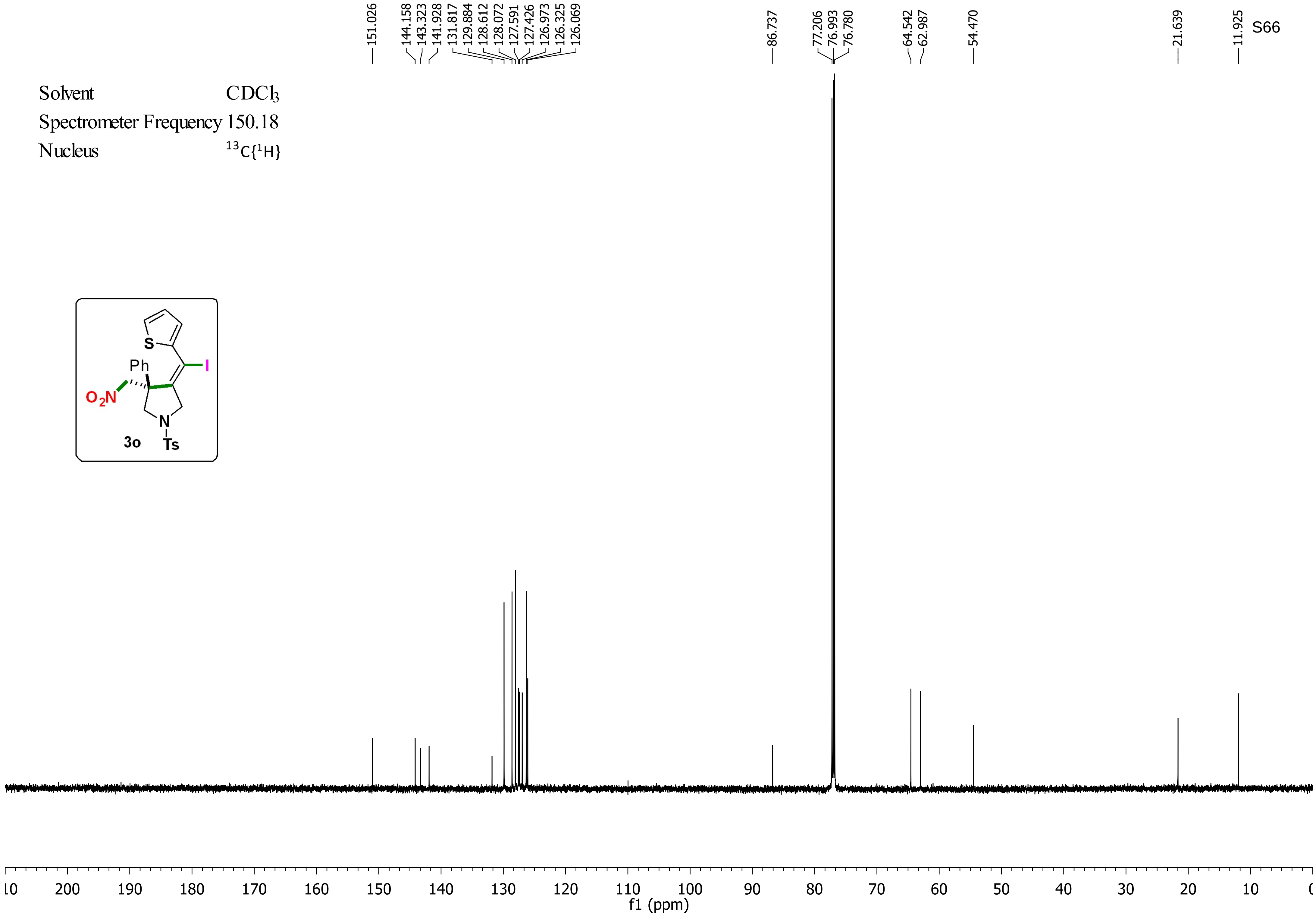

Solvent CDCl$_3$
Spectrometer Frequency 399.44
Nucleus ^{1}H

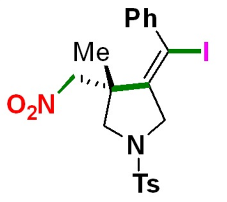

3p (*E*/Z = 10:90)

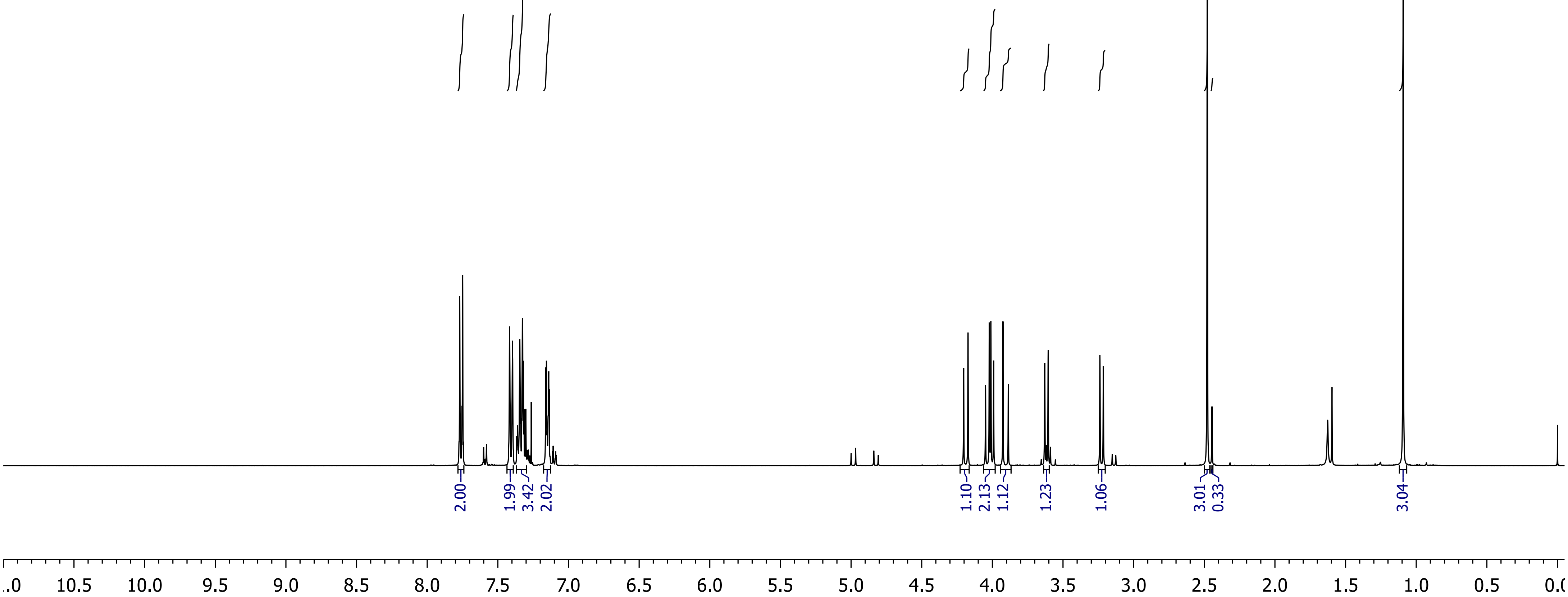

Solvent CDCl$_3$
Spectrometer Frequency 100.45
Nucleus $^{13}C\{^1H\}$

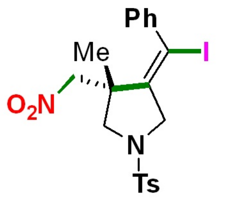

3p (*E*/Z = 10:90)

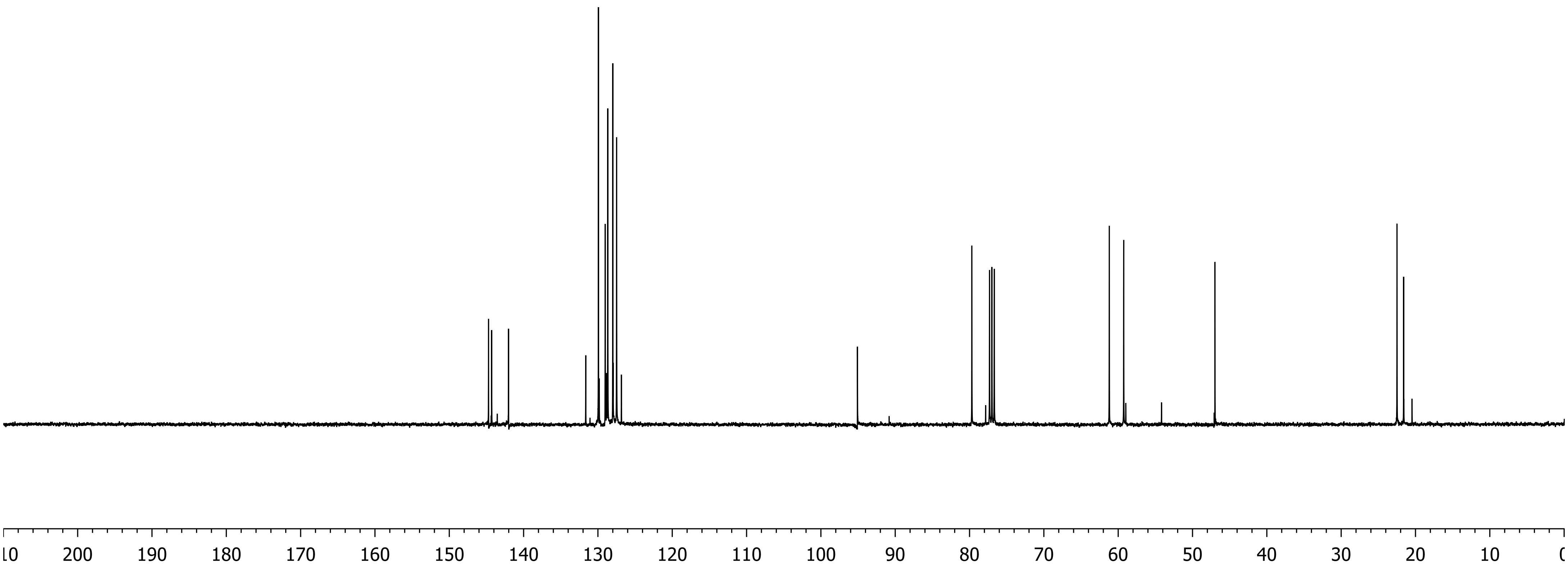

Solvent CDCl3
Spectrometer Frequency 400.40
Nucleus ^{1}H

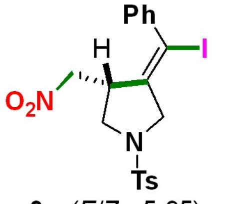

3q (*E*/*Z*= 5:95)

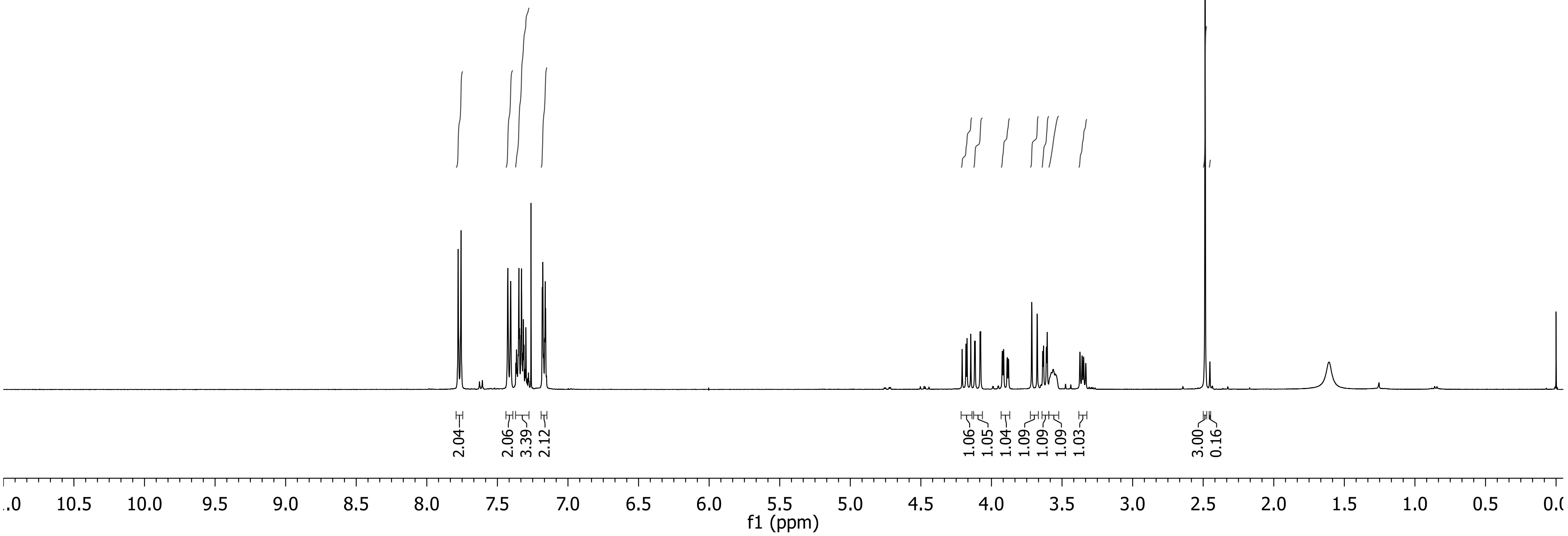

Solvent CDCl$_3$
Spectrometer Frequency 100.69
Nucleus $^{13}C\{^1H\}$

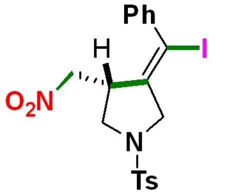

3q (*E*/*Z*= 5:95)

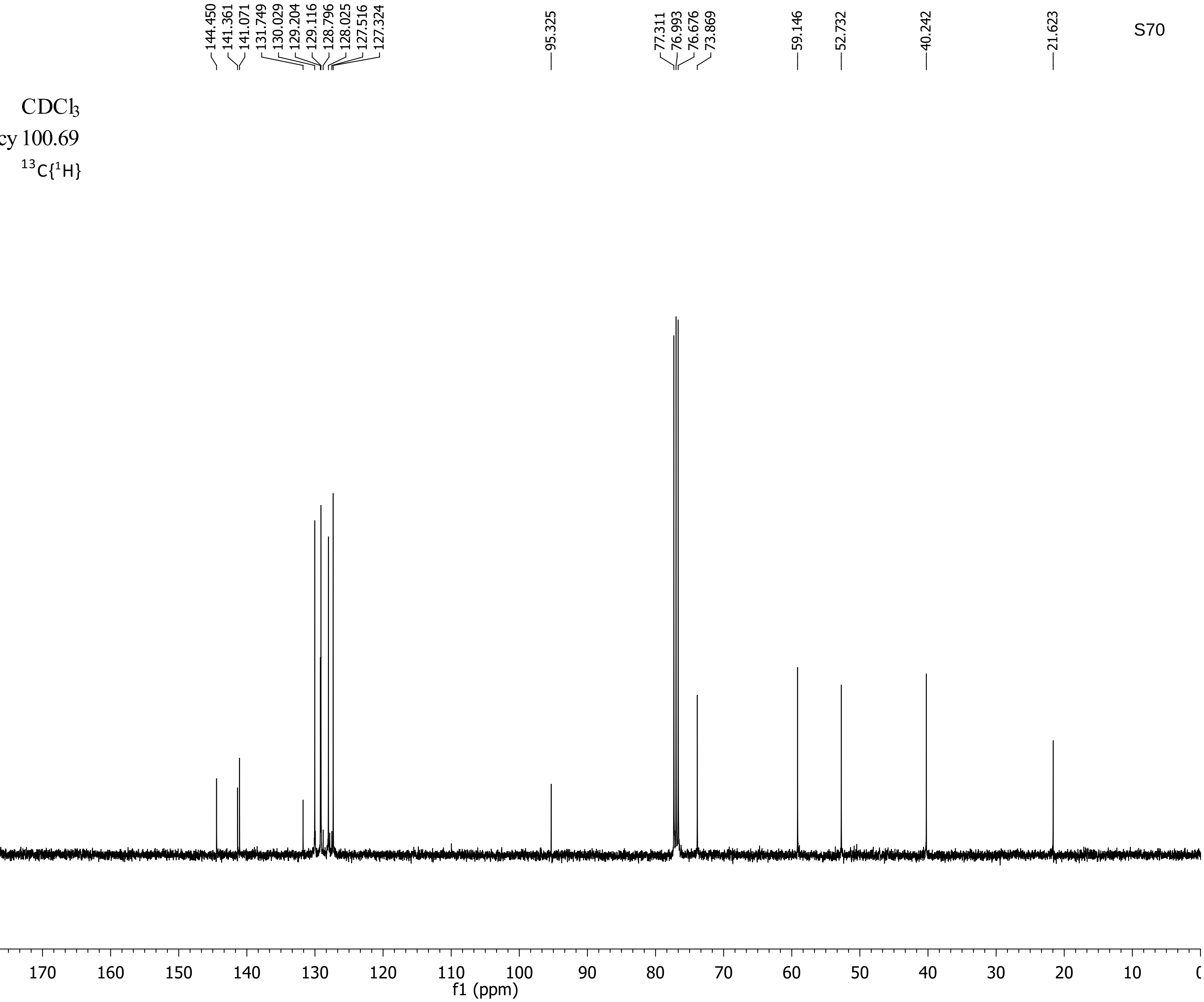

Solvent CDCl3
Spectrometer Frequency 399.44
Nucleus ^{1}H

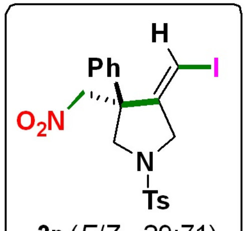

3r (*E*/*Z*= 29:71)

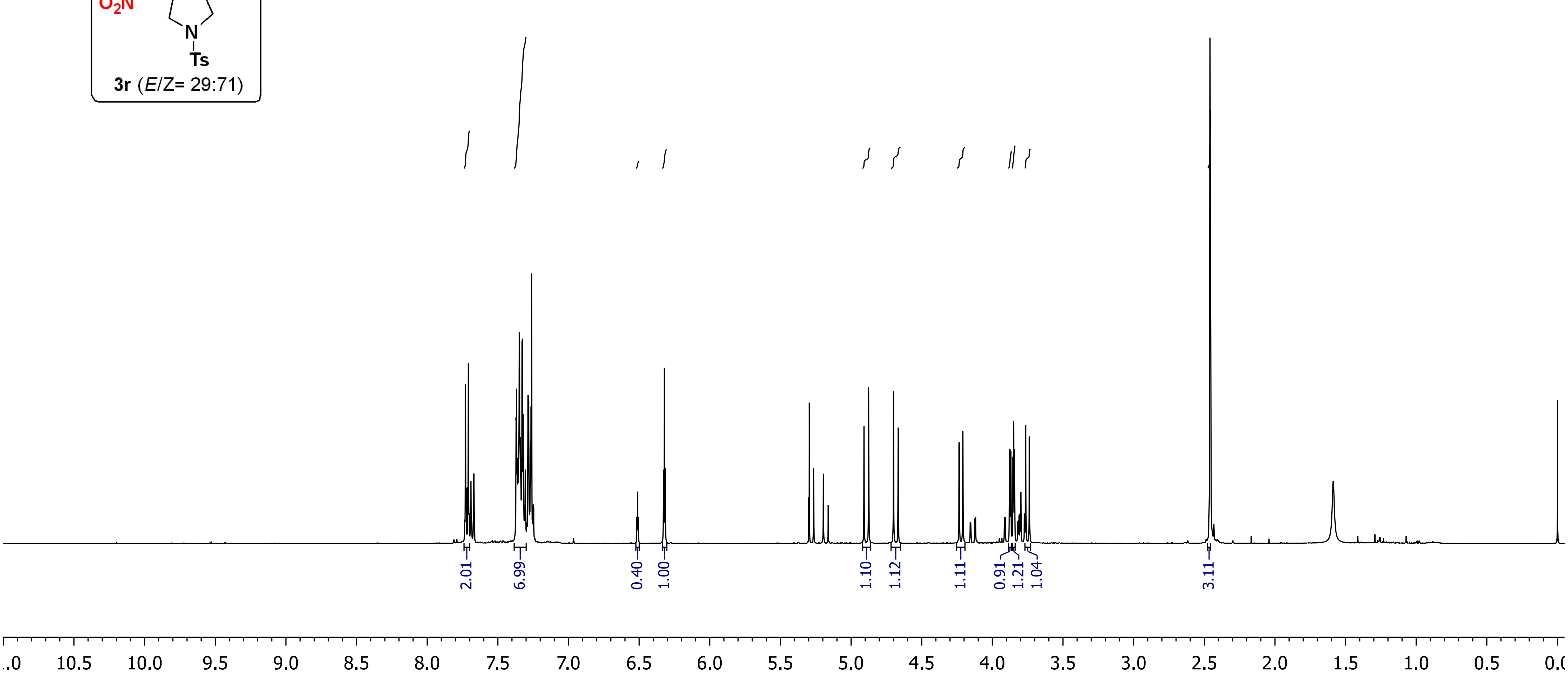

Solvent CDCl3

Spectrometer Frequency 100.45

Nucleus $^{13}C\{^1H\}$

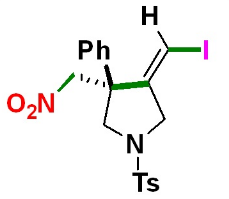

3r (*E*/*Z*= 29:71)

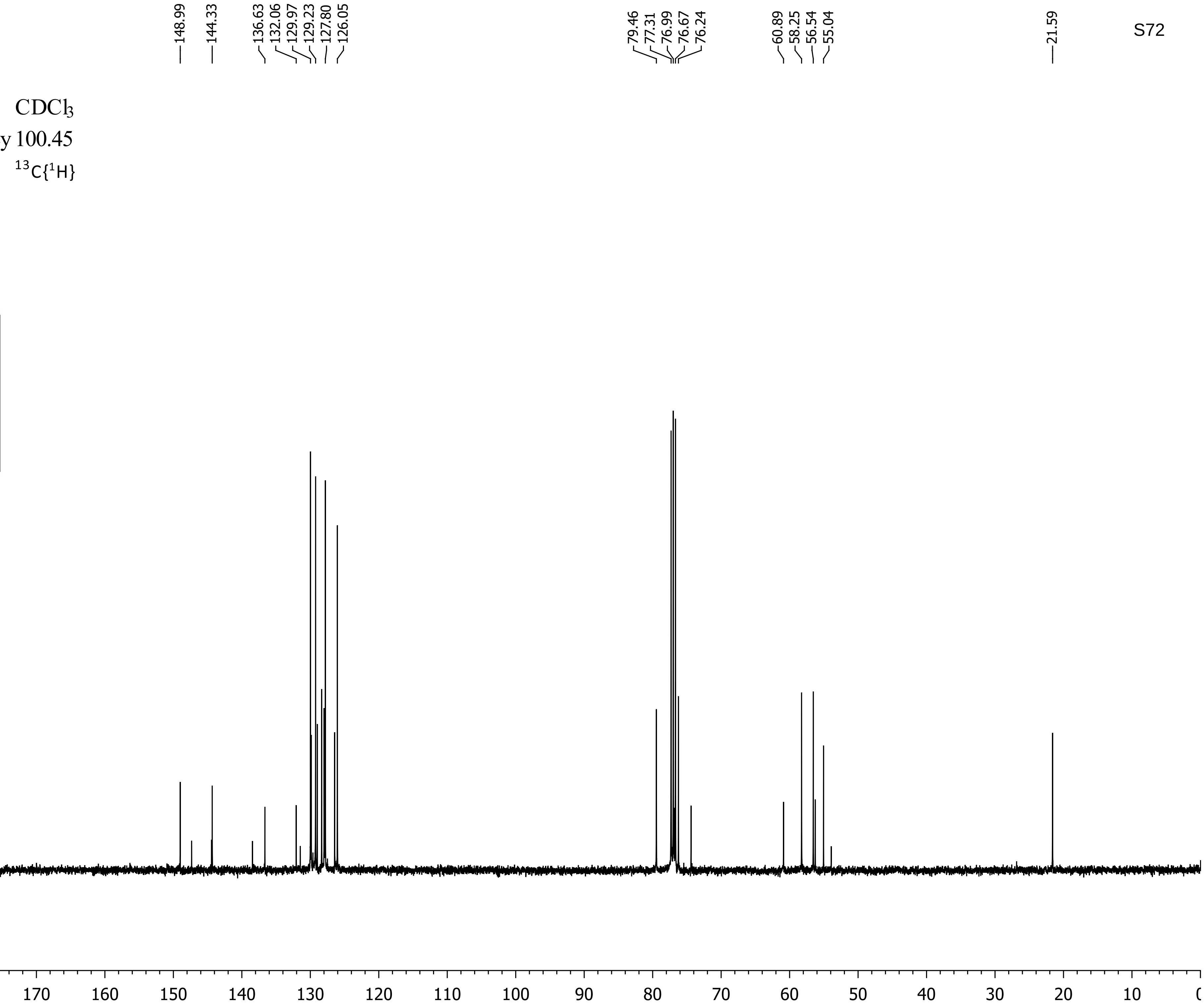

Solvent	$CDCl_3$
Spectrometer Frequency	399.44
Nucleus	^{1}H

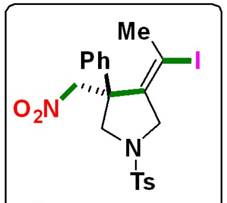

3s (*E*/*Z*= 43:57)

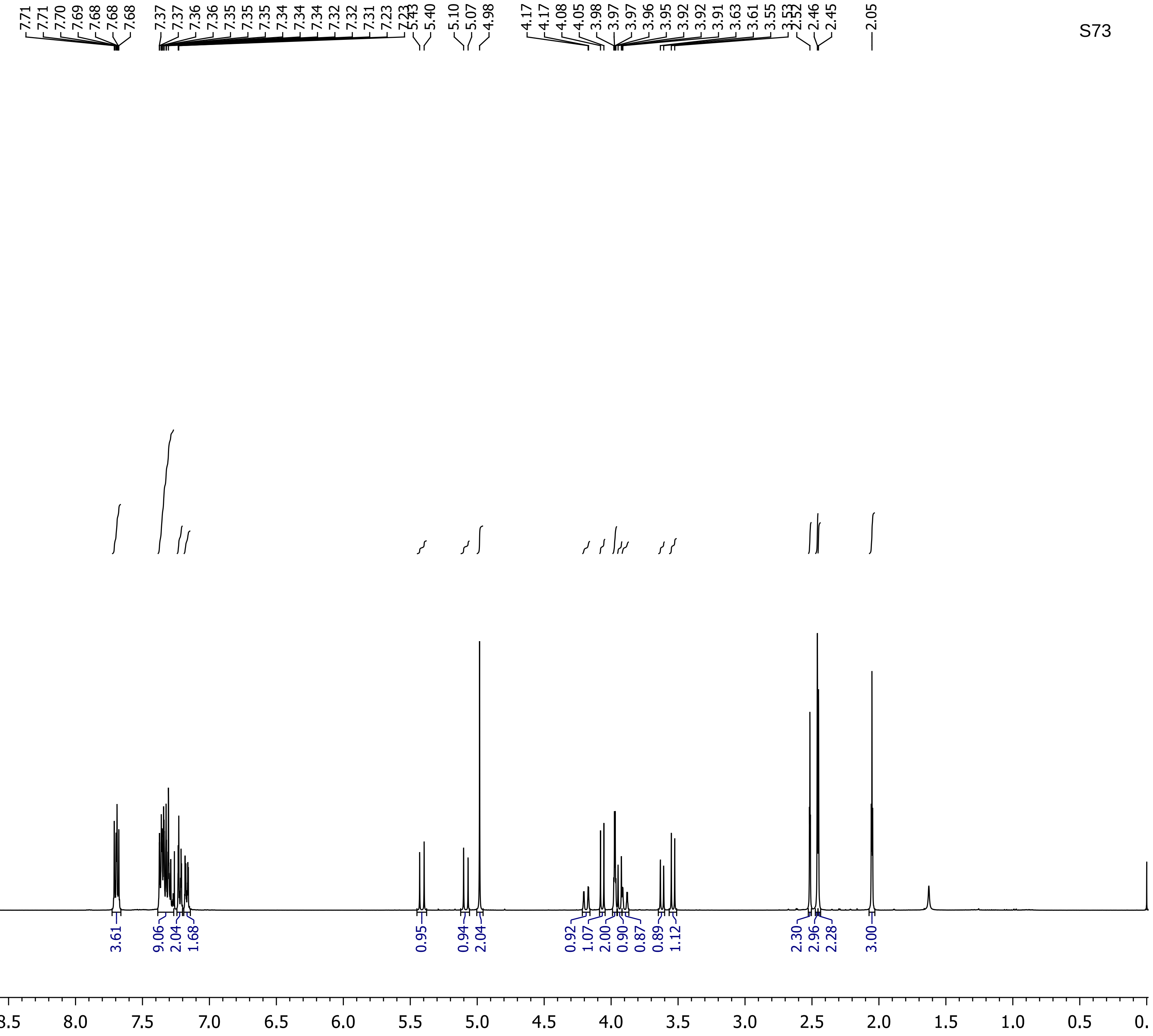

Solvent CDCl3
Spectrometer Frequency 100.45
Nucleus $^{13}C\{^1H\}$

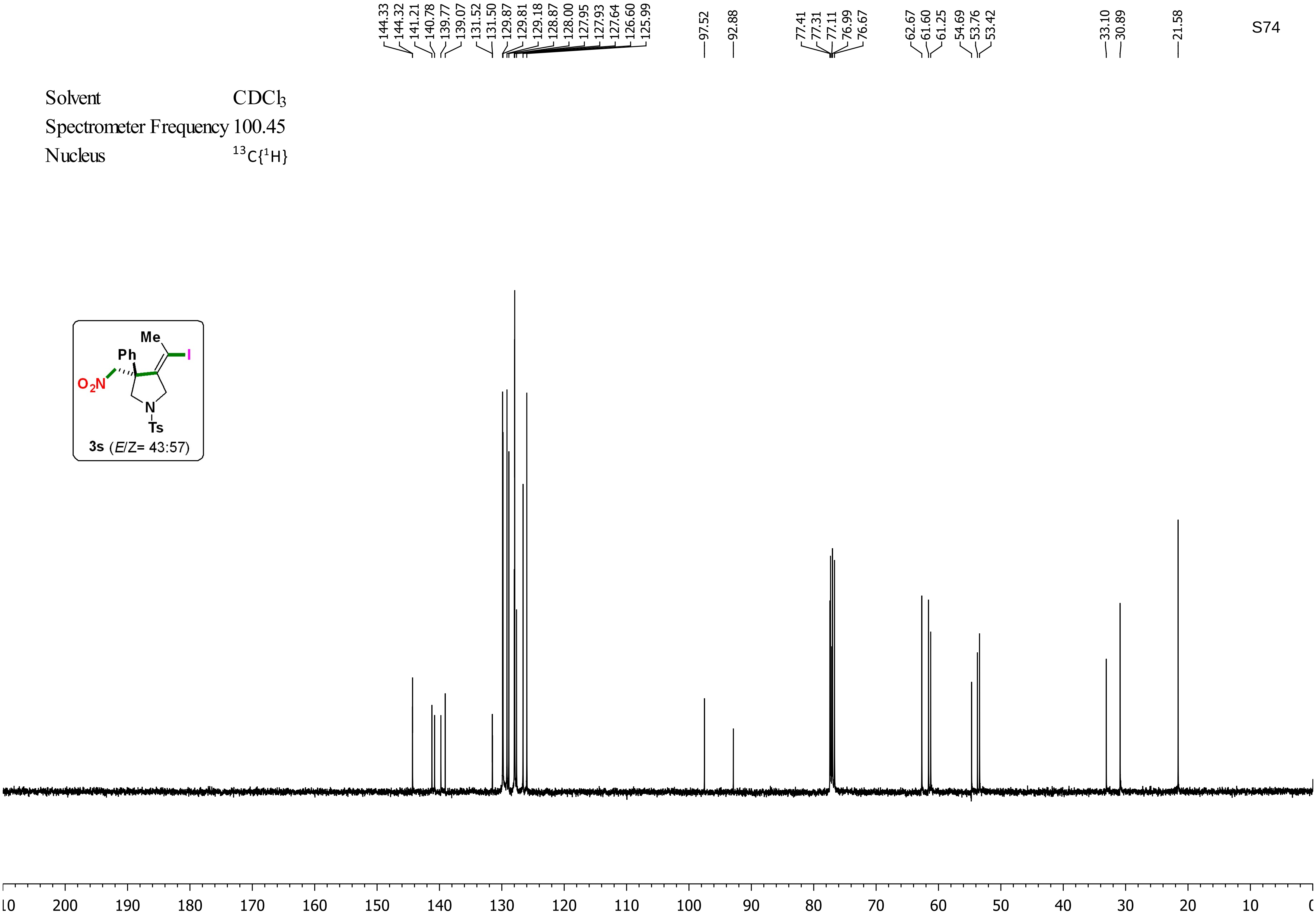

3s (*E*/*Z*= 43:57)

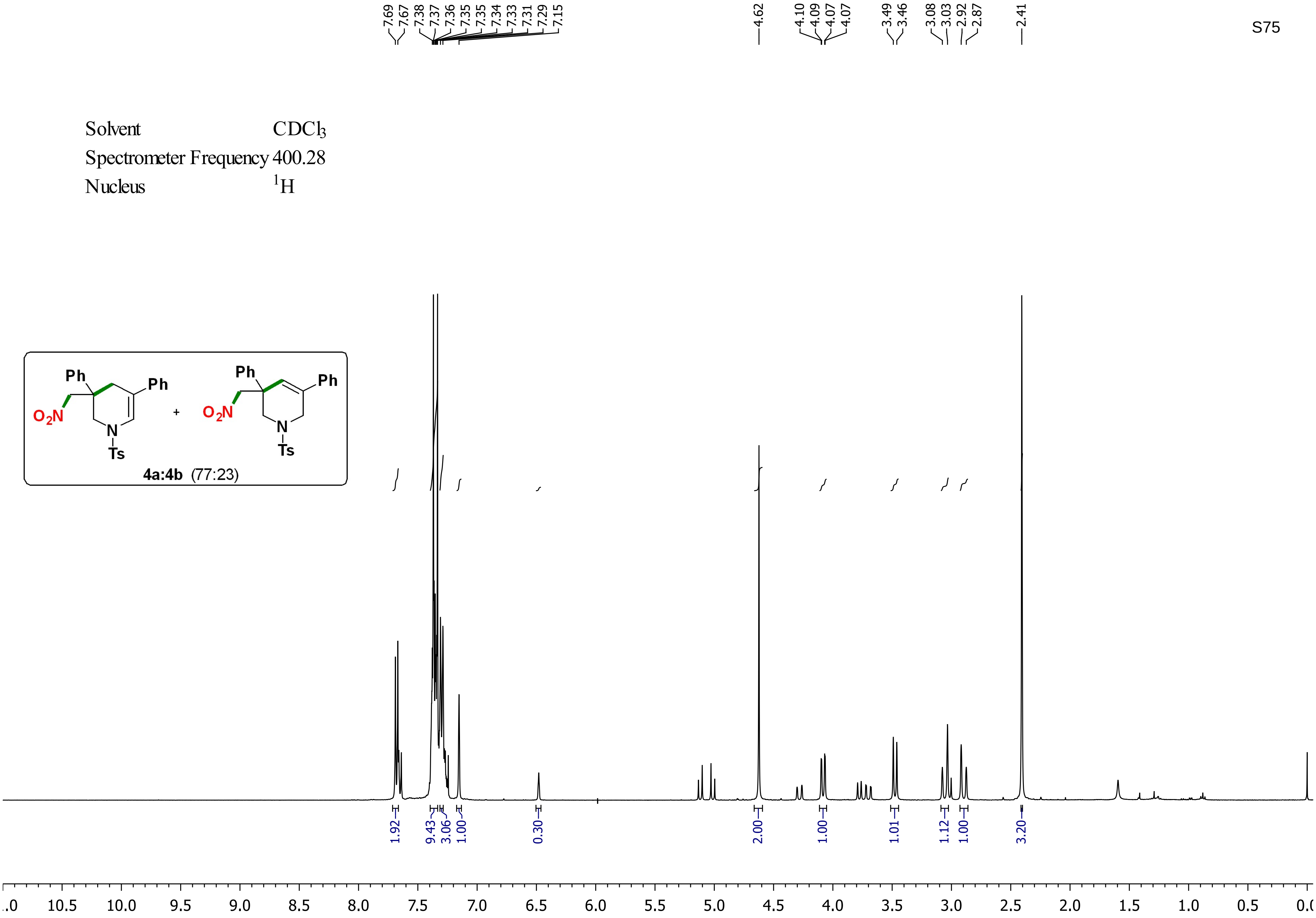

Solvent CDCl3
Spectrometer Frequency 400.28
Nucleus 1H
Ph
Ph
O2N
N
Ts
+
Ph
Ph
O2N
N
Ts
4a:4b (77:23)
7.69 7.67 7.38 7.37 7.36 7.35 7.35 7.34 7.33 7.31 7.29 7.15
4.62
4.10 4.09 4.07 4.07
3.49 3.46
3.08 3.03 2.92 2.87
2.41
1.92 9.43 3.06 1.00 0.30 2.00 1.00 1.01 1.12 1.00 3.20
10.5 10.0 9.5 9.0 8.5 8.0 7.5 7.0 6.5 6.0 5.5 5.0 4.5 4.0 3.5 3.0 2.5 2.0 1.5 1.0 0.5

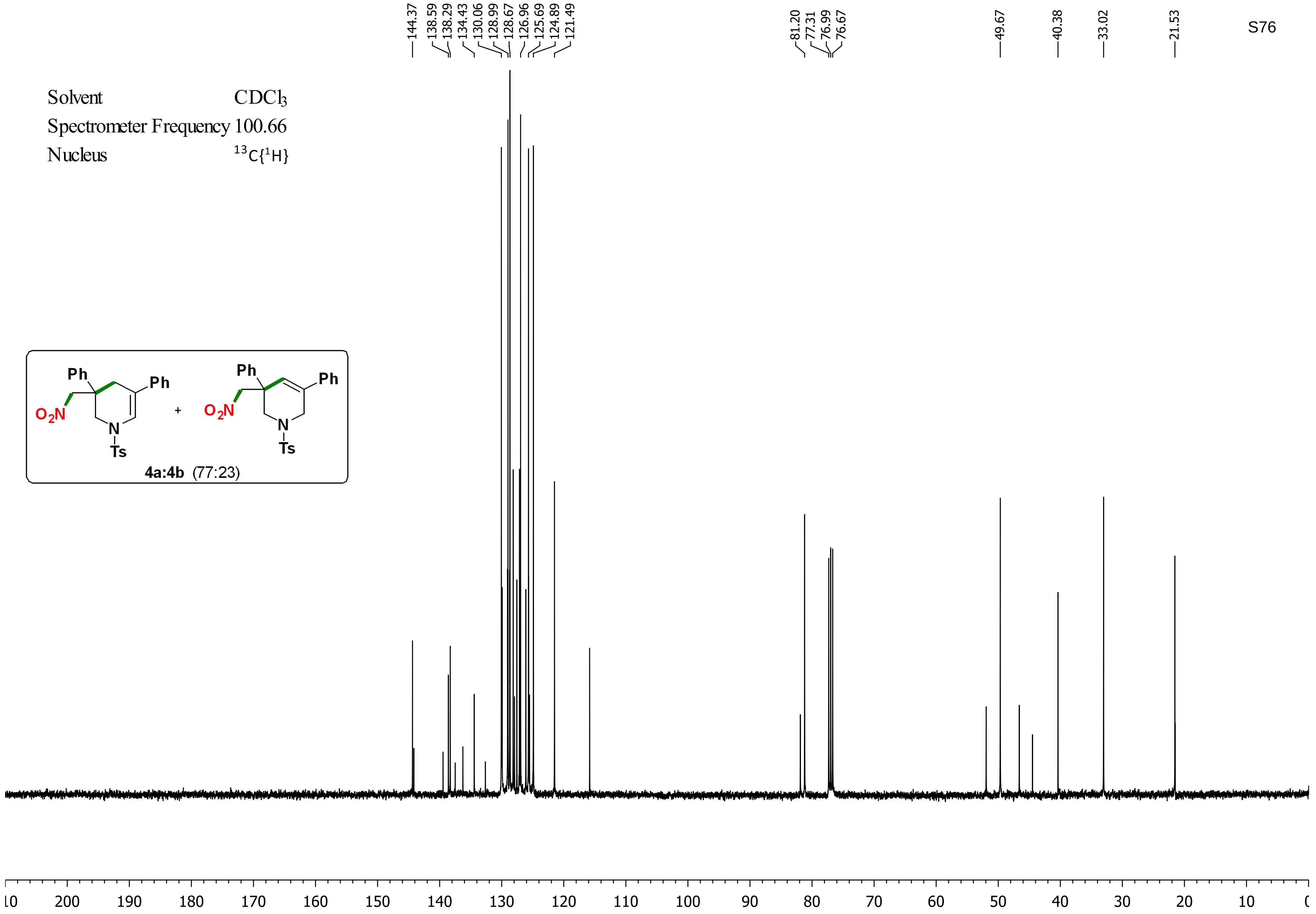
144.37
138.59
138.29
134.43
130.06
128.99
128.67
126.96
125.69
124.89
121.49
81.20
77.31
76.99
76.67
49.67
40.38
33.02
21.53
Solvent CDCl3
Spectrometer Frequency 100.66
Nucleus 13C{1H}
Ph
Ph
O2N
N
Ts
+
4a:4b (77:23)
200
190
180
170
160
150
140
130
120
110
100
90
80
70
60
50
40
30
20
10
